# Elucidating the role of N-myristoylation in the excessive membrane localization of PD-L1 in hypoxic cancers and developing a novel NMT1 inhibitor for combination with immune checkpoint blockade therapy

**DOI:** 10.1186/s13046-025-03438-z

**Published:** 2025-07-02

**Authors:** Haoming Zhao, Zhen Zhang, Chaojun Zhang, Hexin Ma, Qingqing Wan, Xinran Zhao, Xu Wang, Ming Yan, Haiyan Guo, Jianjun Zhang, Wantao Chen

**Affiliations:** 1 Department of Oral and Maxillofacial-Head and Neck Oncology, Shanghai Ninth People’s Hospital, Shanghai Jiao Tong University School of Medicine, College of Stomatology, Shanghai Jiao Tong University, Shanghai, China; 2 Shanghai Key Laboratory of Stomatology, National Center for Stomatology, National Clinical Research Center for Oral Diseases, Shanghai Research Institute of Stomatology, Shanghai, China; 3 Digital Diagnosis and Treatment Innovation Center for Cancer, Institute of Translational Medicine, Shanghai Jiao Tong University, Shanghai, China; 4 Department of Clinical Laboratory, Shanghai Ninth People’s Hospital, Shanghai Jiao Tong University School of Medicine, College of Stomatology, Shanghai Jiao Tong University, Shanghai, China

**Keywords:** Hypoxia, PD-L1, NMT1, Immune checkpoint blockade, N-myristoylation

## Abstract

**Background:**

Most cancers, including head and neck squamous cell carcinoma (HNSCC), frequently exhibit an approximately 80% lack of response to immune checkpoint blockade (ICB) therapy, largely attributed to hypoxia-induced tumor immune suppression. Although hypoxia is known to upregulate PD-L1 expression, the key mechanisms by which it enhances PD-L1 membrane localization and high expression remain elusive.

**Methods:**

We investigated the molecular mechanisms by which hypoxia enhances PD-L1 membrane localization in HNSCC cells. Additionally, we tested the efficacy of combining an anti-PD-1 antibody with the NMT1 inhibitor PCLX-001 in HNSCC xenograft mice and conducted a retrospective clinical study to assess NMT1 as a prognostic biomarker.

**Results:**

Our study revealed that hypoxia-inducible factor-1α (HIF1α) upregulates N-myristoyltransferase 1 (NMT1), which mediates the myristoylation of calcineurin B homologous protein 1 (CHP1). Myristoylated CHP1 binds to PD-L1, facilitating its rapid translocation to the cell membrane and increasing PD-L1-mediated immune evasion. The NMT1 inhibitor low-dose PCLX-001 blocks CHP1 myristoylation, disrupting excessive PD-L1 membrane localization and attenuating cancer immune suppression. In HNSCC xenograft mice, administering an anti-PD-1 antibody combined with low-dose PCLX-001 via intratumoral injection significantly improved the treatment response rate and produced synergistic anticancer effects with no significant weight loss. Furthermore, our retrospective clinical study demonstrated that NMT1 protein levels can serve as an independent prognostic biomarker for HNSCC.

**Conclusion:**

These findings provide robust theoretical support for the translational application of combining NMT1 inhibitors and ICB therapy in cancers under hypoxic conditions. This study introduces a combined cancer therapy strategy named "spatial blockade plus signaling inhibition of PD-L1."

**Supplementary Information:**

The online version contains supplementary material available at 10.1186/s13046-025-03438-z.

## Introduction

Head and neck squamous cell carcinoma (HNSCC), a type of malignant solid tumor derived from epithelial cells, is a formidable global health challenge, ranking as the seventh most common cancer worldwide, with significant implications beyond mortality statistics [1, 2]. Alarmingly, survivors of HNSCC face the second-highest suicide rate among cancer survivors, surpassed only those with pancreatic cancer, with rates of 63.4 and 86.4 cases per 100,000 individuals [3]. This stark reality underscores the profound psychological and physical toll of the disease [1]. Despite employing a range of comprehensive treatments, including surgery, radiotherapy, and chemotherapy, and the emerging field of immunotherapy, the 5-year survival rate is approximately 50%, indicating an urgent demand for innovative therapeutic approaches [4].

The use of programmed death ligand 1 (PD-L1) monoclonal antibodies, such as pembrolizumab, has emerged as a potential immunotherapy for HNSCCs, with a 20%-30% response rate. Compared with other immune checkpoint inhibitors (ICIs), such as CTLA-4 or LAG-3, they have higher clinical response rates [5–7]. This advancement has prompted a closer examination of the tumor microenvironment (TME) of HNSCCs, an intricate ecosystem composed of immune cells, cancer cells, fibroblasts, endothelial cells, and the extracellular matrix [8, 9]. A defining characteristic of this environment is hypoxia, which not only promotes tumor progression and treatment resistance but also contributes to immunosuppression( [10, 11]. Under hypoxic conditions, the expression of programmed death ligand 1 (PD-L1), a critical immune checkpoint molecule, is notably upregulated, facilitating tumor immune evasion in some cancers, especially head and neck cancer, cervical cancer and lung cancer [12, 13]. The mechanism of ICI treatment for cancers involves merely neutralizing PD-L1 on the surface of the cancer cell membrane through PD-1 antibodies and then eliminating immune escape from T cells. Can the immune escape of cancer cells be effectively eliminated by directly inhibiting the membrane translocation of PD-L1? Thus, designing treatments that inhibit the membrane translocation of PD-L1 combined with an anti-PD-1 antibody improves the response rate and effectiveness through two completely different treatment strategies. This is becoming a cutting-edge and key topic in the global ICI treatment field.

Transmembrane proteins frequently shuttle between the endomembrane system and the cell membrane, with the endomembrane system comprising the endoplasmic reticulum (ER), Golgi apparatus, and lysosomes [14, 15]. The spatiotemporal distribution and dynamics of PD-L1 in the plasma membrane are strictly regulated. Newly synthesized PD-L1 proteins in the rough endoplasmic reticulum undergo posttranslational modifications (PTMs) [16]. STT3A and STT3B mediate N-glycosylation of PD-L1 on the ER, stabilizing and upregulating intracellular PD-L1, whereas B3GNT3 mediates PD-L1 glycosylation to facilitate its interaction with PD-L1 on the cell surface [17]. Thyroid adenoma-associated gene (THADA) regulates PD-L1-specific ER-to-Golgi export by coupling PD-L1 with Sec24A-mediated COP2 vesicles, thereby maintaining PD-L1 residence in the Golgi and expression in tumor cells [18]. Hsc70 promotes antitumor immunity by targeting PD-L1 for lysosomal degradation, suggesting a novel therapeutic strategy for cancer immunotherapy [19]. DHHC3/ZDHHC9-mediated palmitoylation blocks PD-L1 ubiquitination and endosomal sorting complex assembly, which are necessary for transport-mediated sorting to multivesicular bodies/lysosomes [20]. The membrane localization of PD-L1 is critical for its function in mediating immune suppression. When PD-L1 is correctly positioned on the cell membrane, it can effectively bind to PD-1 on activated T cells, leading to T-cell inactivation and promoting an immunosuppressive tumor environment [13].

Turning our focus to a less explored but equally intriguing aspect, N-myristoylation, a type of protein fatty acid acylation, has been linked to critical cellular functions, including membrane binding, protein transport, and interaction [21, 22]. Recent findings suggest that N-myristoylation, particularly through the upregulation of N-myristoyltransferase 1 (NMT1) in hypoxic tumor environments, could play a pivotal role in cancer progression, given its involvement in cell proliferation, the cell cycle, and death pathways [23]. Our research revealed that hypoxia not only increased NMT1 expression but also enhanced overall protein N-myristoylation, altering the distribution of PD-L1 to favor its presence on the cell membrane, potentially intensifying its immunosuppressive effects. These findings suggest that the modulation of N-myristoylation under hypoxic conditions could be a key factor in the therapeutic landscape of HNSCC, especially considering the critical role of N-myristoylation and the design of novel strategies involving PD-L1 membrane localization in immune suppression.

## Materials and methods

### Patients and specimens

All samples were collected from the Department of Oral and Maxillofacial Head and Neck Oncology, Ninth People's Hospital, Shanghai Jiao Tong University School of Medicine (Shanghai, China). Patients in this study were diagnosed and underwent surgical treatment between April 16, 2018, and July 17, 2018. None of the patients had received chemotherapy or immunotherapy. The age of the patients ranged from 44 to 72 years, with a male-to-female ratio of [5.7:4.3]. According to medical records, the weight range of the patients was between 55 and 102 kg. All patients were followed for 60 months. The clinical stage was determined according to the 8th edition of the American Joint Committee on Cancer (AJCC) staging manual [24].

### Cell culture

The human oral squamous cell carcinoma cell lines SCC25 (RRID:CVCL_1682), SCC9 (RRID:CVCL_1685), and CAL27 (RRID:CVCL_1107) and the mouse oral squamous cell carcinoma cell line SCC7 (RRID:CVCL_V412) were purchased from the American Type Culture Collection (ATCC, USA). KYSE-510 (RRID: CVCL_1354) and SIHa (RRID: CVCL_0032) HEK-293T (RRID: CVCL_0063) cells were purchased from Shanghai Fuheng Biotechnology Co., Ltd. CAL27, SCC7, SIHa, HEK-293T, and KYSE-510 cells were cultured in DMEM (BasalMedia, China) supplemented with 10% fetal bovine serum (FBS; Gibco-BRL, USA), penicillin (100 units/mL), and streptomycin (100 μg/mL) at 37°C in a humidified 5% CO_2_/95% air incubator, whereas SCC25 and SCC9 cells were maintained in DMEM/F‐12 (BasalMedia, China) supplemented with the same additives. Hypoxic treatment of the cells was performed in a tri‐gas incubator (Thermo Fisher Scientific, USA) flushed with a gas mixture of 1% O_2_, 5% CO_2_ and balanced N_2_.

### Lentivirus packaging shRNA and plasmid transfection

To effectively knock down and overexpress NMT1, we used lentivirus-packaged shRNAs and plasmids with synonymous mutations to infect cells and screened the cells with puromycin or blasticidin to establish stable knockdown or overexpression cell lines. Related lentivirus products were purchased from Genomeditech Co., Ltd. (Shanghai, China). For the site mutation of the N-terminal myristoylation modification signal of CHP1, we designed the CHP1-NmyrMT (N-myristoylation modification sequence mutation) plasmid containing the 3xFlag sequence and the 3xFlag-CHP1-WT plasmid. For the fluorescence colocalization experiment, we designed CHP1-NmyrMT-mCherry, CHP1-WT-mCherry, and PD-L1-eGFP (RRID:Addgene_120933) plasmids, all of which were purchased from Genomeditech Co., Ltd. (Shanghai, China). Lipofectamine 3000 was used as a plasmid transfection reagent (Thermo Fisher Scientific).

### Reagents and antibody generation

The chemicals and their sources were myristic acid alkyne (YnMyr) (Cayman Chemical, cat. no. 13267), AzT 6b (Click Chemistry Tools; cat. no. AZ109-5), EDTA (UltraPure 0.5 M, pH 8.0; Invitrogen, cat. no. 15575020), and copper sulfate (CuSO4) (Sigma‒Aldrich, cat. no. 451657-50G), tris (2-carboxyethyl)phosphine (TCEP; Sigma‒Aldrich; cat. no. C4706-2 g), and TBTA (Sigma‒Aldrich, cat. no. 678937-50 MG), analytical-grade methanol (VWR, cat. no. 20847.307), analytical grade (Sigma‒Aldrich, cat. no. C2432-1 L), (4-(2-Hydroxyethyl)piperazine-1-ethanesulfonic acid (HEPES) (Sigma‒Aldrich; cat. no. H4034-500G), and NaOH pellets (Sigma‒Aldrich, cat. no. S8045-500G), and azide-biotin (Click Chemistry Tools, cat. no. 1265-25), (ditothreitol DTT (;) (Sigma‒Aldrich, cat. no. 43815), NeutrAvidin-coupled agarose beads (Pierce; cat. no. 29201), streptavidin-coupled magnetic beads (New England Biolabs; cat. no. S1420S), N-myristoyltransferase inhibitor PCLX-001(Topscience; cat. no. 1215011-08-7) and PEG3000(MedChemExpress, cat. no. HY-Y0873N) and Tween 80 (MedChemExpress, cat. no. HY-Y1891). The in vivo antibodies used for the mouse models were as follows: control antibody against the InVivoMab rat IgG2b isotype (#BE0090; 25 mg; Bioxcell) and anti-PD-1 Ab (#BE0146; 25 mg; Bioxcell). The following antibodies were used for flow cytometry analysis: PE/Cyanine7 anti-human CD274 (MIH3) antibody (#374506, 1:200, BioLegend). The following antibodies were used for protein coimmunoprecipitation: anti-PD-L1 (Abcam, 2.5 µg/IP, ab228415), anti-CHP1 (Genetex, 2.5 µg/IP, GTX113936), and anti-FLAG M2 (Sigma, 5 µg/IP, F1804) antibodies. The following antibodies were used for protein chromatin immunoprecipitation: anti-HIF-1 alpha antibody (Novus Biologicals, 2.5 µg/IP, NB100-105). The primary antibodies used for immunoblotting were as follows: anti-panmyristoylation antibody (LifeSpan Biosciences, 1:5000, LS-C813601), anti-NMT1 antibody (Proteintech, 1:1000, 11546-1-AP), anti-NMT2 antibody (Genetex, 1:1000, GTX115976), anti-CHP1 antibody (GeneTex, 1:2000, GTX113936), anti-PD-L1 antibody (Proteintech, 1:1000, 66248-1-Ig), anti-DYKDDDDK tag antibody (Proteintech, 1:1000, 20543-1-AP), and anti-Na^+^/K^+^-ATPase α1 antibody (abbkine, 1:1000, ABL1141). The secondary antibodies used for immunoblotting were as follows: goat anti-rabbit IgG HRP antibody (Affinity Biosciences, 1:10000, S0001) and goat anti-mouse IgG HRP antibody (Affinity Biosciences, 1:10000, S0002). The primary antibodies used for immunofluorescence were as follows: anti-PD-L1 (clone 5H1) antibody (Sigma‒Aldrich, 1:50, MABC1115), anti-HIF1α antibody (Novus Biologicals, 1:50, NB100-105), anti-CD8a antibody (Santa Cruz Biotechnology, 1:50, sc-7970), anti-GZMB antibody (Santa Cruz Biotechnology, 1:50, sc-8022), anti-PDCD1 antibody (eBiosciences, 1:100, 14-2798-82), anti-CD3 antibody (eBiosciences, 1:200, 14-0037-82), and anti-NMT1 antibody (Proteintech, 1:100, 11546-1-AP). The fluorescent secondary antibodies used for immunofluorescence were goat anti-mouse-488 (Proteintech, 1:200; CTK0103), goat anti-rabbit-555 (Proteintech, 1:200; RAGR003), and donkey anti-mouse-647 (Thermo Fisher Scientific, 1:200; A-31571) antibodies. The antibody used for immunohistochemistry (IHC) was an anti-NMT1 antibody (Proteintech, 1:100, 11546-1-AP).

### Bioinformatics analysis

To perform immune cell infiltration deconvolution via CIBERSORT, we first prepared gene expression data from The Cancer Genome Atlas (TCGA) head and neck squamous cell carcinoma (HNSC) samples and normalized the mRNA counts to meet the input requirements of the algorithm. The analysis utilized the pretrained LM22 signature matrix, which contains expression profiles of 22 human immune cell subsets, including effector regulatory T cells, M0/M1/M2 macrophages, exhausted T cells, naive/memory B/T cells, γδ T cells, activated mast cells, plasma cells, activated myeloid dendritic cells, activated natural killer cells, CD8 + T cells, and follicular helper T cells. The input gene expression matrix (samples × genes) was formatted with Entrez gene IDs and log2-transformed to ensure compatibility with the signature matrix. We then executed the CIBERSORT algorithm via its web interface (https://cibersort.stanford.edu/) or command-line tool, setting parameters: perm = 1000 for permutation testing to assess the statistical significance of cell type estimates and QN = TRUE to apply quantile normalization between the query dataset and the signature matrix, minimizing technical variation. The algorithm calculates the relative proportion of each immune cell subset in individual tumor samples by solving a least-squares problem that optimizes the match between the query expression matrix and the linear combination of signature cell profiles.

Following deconvolution, we extracted the estimated infiltration fractions for the 22 immune cell types. These fractions were then subjected to correlation analysis with hypoxic signature scores derived from TCGA mRNA data, and Spearman’s rank correlation coefficient was used to evaluate associations between immune cell infiltration and hypoxia levels in the TME. This workflow enabled systematic estimation of immune cell composition and its relationship with hypoxic stress in HNSC samples.

### Protein extraction and immunoblotting

The cells were lysed in radioimmunoprecipitation assay (RIPA) buffer (Biosharp, China) containing protease and phosphatase inhibitors. The lysates were cleared via centrifugation, and the protein concentrations were determined via v. The proteins were separated via SDS‒PAGE and transferred to PVDF membranes (Millipore, Merck KGaA, Germany). The membranes were blocked with 5% nonfat milk in Tris-buffered saline with 0.1% Tween-20 (TBST) and probed with primary antibodies. Secondary antibodies conjugated to horseradish peroxidase were used for detection with an enhanced chemiluminescence (ECL) substrate (Millipore, Merck KGaA, Germany).

### Immunofluorescence

SCC9 and HEK-293T cells were cultured on glass slides, fixed with 4% paraformaldehyde for 20 min, and permeabilized with PBS containing 0.1% Triton X-100 for 1 h at room temperature. The tissue sections were dried in an oven at 60°C for at least 2 h, followed by dewaxing and hydration. The sections were subjected to antigen retrieval in citrate buffer and blocked with 5% BSA. The primary antibody was incubated overnight at 4°C, and the secondary antibody was incubated at room temperature for 1 h. The nuclei were stained with DAPI, and the sections were mounted with anti-fade mounting medium (Beyotime). All steps were performed under light-protected conditions to preserve the activity of the fluorescent dyes. Images were obtained via a Zeiss LSM900 microscope. During the assessment of pathology slides via the H-Score and IRS methods, the evaluators were blinded to the sample group information to minimize the impact of subjective bias on the results. Immunofluorescence intensity analysis was performed via ImageJ Fiji (RRID:SCR_003070), and statistical analysis was conducted via Prism 10.0 (RRID:SCR_002798).

### Dynamic fluorescence images

To capture dynamic fluorescence images via a confocal microscope, the sample was first prepared by culturing the target cells in appropriate media or preparing tissue sections and labeling the molecules of interest with fluorescent probes through genetic modification, immunofluorescence, or dyes, followed by thorough washing to remove unbound probes. Then, a confocal microscope with lasers and detectors matching the probes was selected, and the objective magnification, pinhole size, laser power, and detector parameters were set for optimal imaging. Next, we define the time-lapse imaging parameters (interval, duration, and z-slices), use autofocus to maintain focus, start the imaging sequence, and save the images in a suitable format. Afterward, the images were processed (background subtraction, noise reduction, alignment), and software such as ImageJ was used for quantitative analysis, such as measuring fluorescence intensity or tracking object movement. Finally, we assessed photobleaching, adjusted the parameters, if necessary, and conducted replicate experiments to ensure the reproducibility of the results.

### Immunohistochemistry

The tumor tissues were rapidly excised, fixed with 4% paraformaldehyde, and embedded in paraffin for tissue sectioning and immunohistochemical staining. Visualization of the cell nuclei was performed with hematoxylin, and analysis was performed via an Olympus BX61 light microscope. Statistical analysis was performed via ImageJ. For the pathological tissue sections of the same patient, three tumor edge areas and three tumor areas were selected, and the evaluation was based on the average H score of the three areas. The H score (∑(pi × i) = (percentage of weak-intensity cells × 1) + (percentage of moderate-intensity cells × 2) + (percentage of strong-intensity cells × 3)), and the H score is between 0 and 300. The larger the value is, the more intense the comprehensive positive expression is in terms of both depth and quantity.

### Chromatin immunoprecipitation

The cells were cross-linked with formaldehyde and lysed, and the chromatin was sheared via sonication. All key reagents used in the experiments were obtained from a ChIP kit (Cell Signaling Technology, #9005). Immunoprecipitation was performed overnight with specific antibodies, followed by washing and elution. The cross-links were reversed, and the DNA was purified via phenol‒chloroform extraction and ethanol precipitation. The enriched DNA fragments were analyzed via PCR and agarose gel electrophoresis. All procedures were conducted at appropriate temperatures, and hazardous chemicals were handled according to safety guidelines.

### Flow cytometry analysis of membrane PD-L1

For the flow cytometric analysis of tumor cell membranes, SCC9 cells were seeded in a 6-well plate, with each group being analyzed at least four times and treated accordingly. The cells were then collected by centrifugation at 1000 × g for 5 min. The tumor cells were not permeabilized and were incubated with PBS (0.5% BSA) at room temperature for 10 min. PE/Cyanine7 anti-human CD274 (MIH3) antibody (#374506, 1:200, BioLegend) and a matched isotype control were used to detect the cells in the dark at 4°C for 30 min. After washing three times with PBS, the cells were analyzed via a flow cytometer (NovoCyte Penteon), and the data were analyzed via NovoExpress v1.6.2 (RRID:SCR_017788) and FlowJo v10.8.1 (RRID:SCR_008520) software.

### Labeling proteins with lipid probes and performing in vitro click reactions

Myristic acid alkyne (Ynmyr) was dissolved in DMSO and added to the culture medium at a final concentration of 20 μM [25]. SCC9 or SCC7 cells were cultured in 10 cm dishes with a medium volume of 12 ml and incubated in an incubator for at least 18 h. During this process, different concentrations of the protein myristoylation inhibitor PCLX-001 were added to the medium. After the protein lysis buffer was added, the dish was placed on ice, the cells were scraped off, and the mixture was subsequently centrifuged at 17000 × g for 10 min at 4°C to collect the supernatant. The protein concentration was measured via a BCA assay kit, and the final lysate concentration was adjusted to 1–2 μg/ml. Every 100 µl of cell lysate is a unit of click reaction sample, into which 6 µl of click reaction solution was added (1 µl-Azb, 2 µl-CuSO4, 2 µl-TCEP).

### Whole-proteome profiling of protein lipidation via chemical proteomics and target validation by immunoblotting

After the click reaction, the protein samples were subjected to protein washing, and the washed protein precipitate was solubilized with ultrasound assistance. NeutrAvidin-coupled agarose beads (Pierce; cat. no. 29201) or streptavidin-coupled magnetic beads (New England Biolabs, cat. no. S1420S) were used to enrich the modified proteins. The enriched proteins were eluted by heating (1/3 volume of 3 × loading buffer, 2 μM Azb). After elution, protein mass spectrometry was performed via a mass spectrometer-compatible HPLC system (EASY-nLC 1000 liquid chromatograph, LC120, Thermo Scientific). After elution, the quantified proteins were subjected to protein electrophoresis, followed by transfer of the proteins from the gel to a PVDF membrane, where the expression levels of the modified proteins were detected via specific antibodies.

### Isolation of membrane proteins and cytoplasmic proteins

The cells were collected, and cytoplasmic and membrane proteins were extracted according to the instructions provided by the Membrane and Cytoplasmic Protein Extraction Kit (BL671B, Biosharp, China).

### In vivo tumor models and treatment

For tumor formation in BALB/c nude mice (RRID:IMSR_CRL:211) injected with SCC9-LishNC or SCC9-LishNMT1 cells, 1 × 10^6^ cells were injected at each injection site. For tumor formation in C3H/He mice (RRID:IMSR_JAX:000659) with SCC7 cells, 1 × 10^6^ cells were injected into each injection site. PCLX-001 was administered by intratumoral injection, with the injection site located at the junction between the tumor and skin. The tumor volume was measured before each drug injection, and PD-1 and IgG2a mAbs were administered via intraperitoneal injection. The tumor size was measured and calculated via the following formula: 1/2 × length × width^2. The mice were randomly divided into 4 groups via either a random number table method or a computer-generated random sequence to ensure the comparability of baseline characteristics across groups. On the day of sacrifice, the final tumor weight was measured, and the tumor tissues were fixed, sectioned, and subjected to immunofluorescence staining.

### Clinical endpoints and statistical analysis

In this retrospective clinical study, the sole clinical endpoint was overall survival (OS). This study focused on patients pathologically diagnosed with head and neck squamous cell carcinoma (HNSC) between April 16, 2018, and July 17, 2018. The calculation of OS commences from the date of diagnosis and is concluded on August 1, 2023. Patient death was defined as the end point. In cases in which patients are still alive at the end of the study, their data are censored.

On the basis of preliminary pilot study results and relevant research data, we estimated the required sample size to be 72 cases to ensure the detection of significant intergroup differences at the predetermined power (1-β = 0.8) and significance level (α = 0.05). The power analysis was conducted via Prism 10.0.

For statistical analysis, the Kaplan‒Meier method was used to analyze the survival data of patients and plot survival curves, which vividly depict the variation in patient survival probabilities over time since diagnosis. The OS trend can be intuitively presented by computing the survival probabilities at each time point. The log-rank test was used for the univariate analysis of potential factors influencing OS. If there are distinct subgroups in the study (e.g., those classified by age, sex, and tumor stage), this test is used to compare the differences in survival curves among subgroups, thereby determining whether these factors significantly impact patient survival times. The test hypothesis presumes that the survival curves of all the subgroups are identical. If the P value was lower than the predetermined significance level (commonly 0.05), the null hypothesis was rejected, suggesting a significant correlation between the factor and patient survival. If multiple factors potentially affecting OS were identified in the univariate analysis, multivariate Cox proportional hazards regression analysis was conducted. This analysis aimed to determine the independent effect of each factor on survival outcomes while controlling for other factors. Each factor was incorporated into the Cox model to calculate the hazard ratio (HR) and its 95% confidence interval (CI) to evaluate the extent to which each factor influences the patients' risk of death. All data processing adhered strictly to data quality management standards to ensure data accuracy and integrity. Statistical analysis was performed via professional statistical software (e.g., Prism or R). During the analysis, missing data were managed appropriately (e.g., by explaining the method for imputing missing values or analyzing the potential impact of missing values on the results) to guarantee the reliability of the analysis results.

## Results

### Hypoxia upregulates protein N-myristoylation in tumor cells via NMT1

**Fig. 1:**
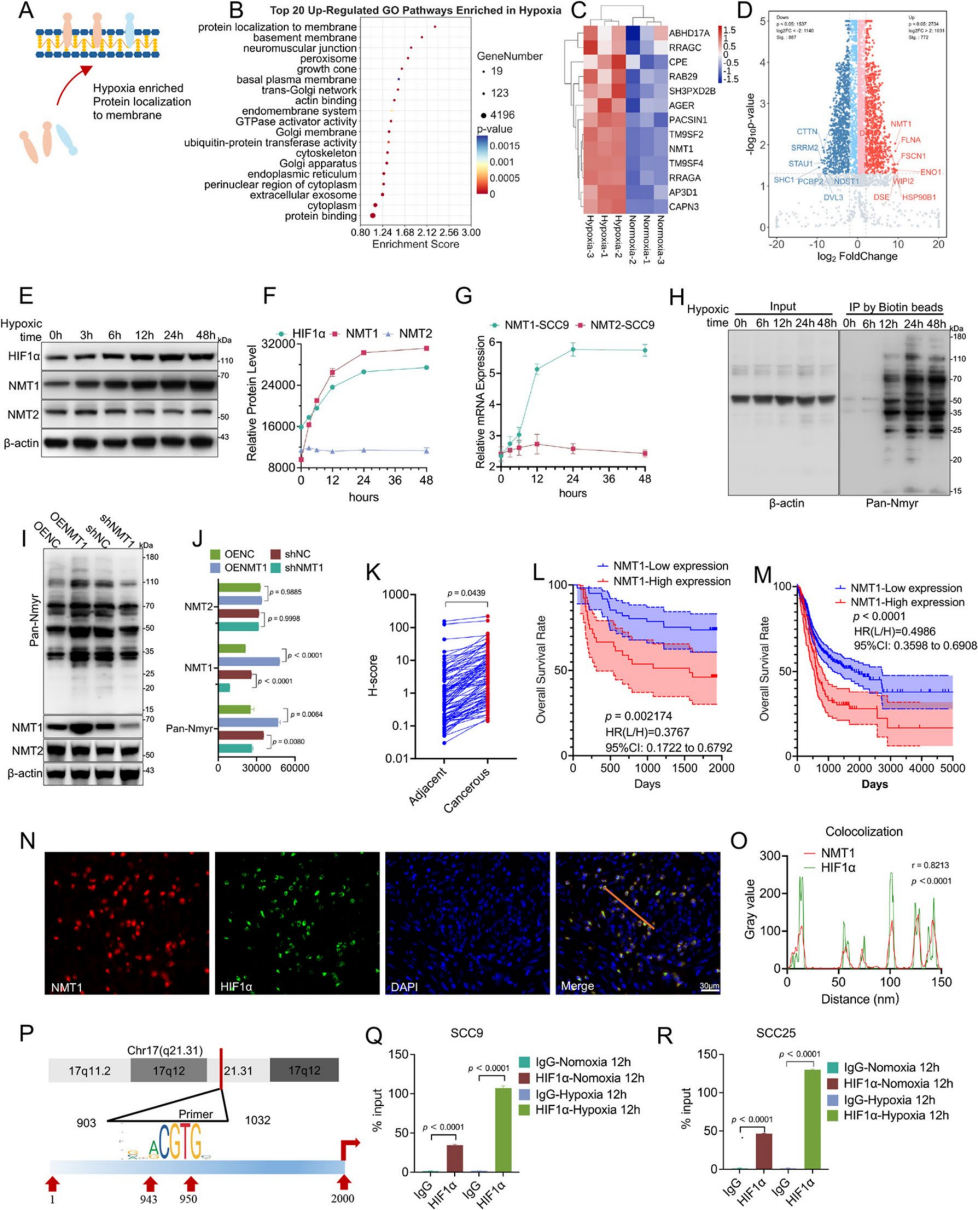
Hypoxic stress increases protein N-myristoylation in tumor cells via NMT1. **A** Schematic representation of protein localization enriched under hypoxia, illustrating the activation of membrane protein transport pathways. **B** The top 20 enriched GO pathways under hypoxic conditions. The "protein localization to membrane" pathway is specifically induced under hypoxia (cell line: SCC9; enrichment score = 2.28; *p* = 5.87 × 10⁻^5^). **C** Hierarchical clustering analysis confirmed that 13 genes related to membrane localization were coupregulated in a hypoxic environment. **D** Gene expression analysis revealed that NMT1 was the most significantly differentially expressed gene (log2FC = 6.67, *p* = 6.9 × 10⁻.^52^). **E** SCC9 cells were treated under hypoxic conditions at 0 h, 3 h, 6 h, 12 h, 24 h, and 48 h, and the expression trends of HIF1α, NMT1 and NMT2 were examined via protein immunoblotting. **F** Quantitative analysis was performed on the protein bands of HIF1α, NMT1, and NMT2 after treatment over a hypoxic time course. **G** Expression of NMT1 and NMT2 at the mRNA level in the SCC9 cell line under hypoxic conditions. **H** Changes in N-myristoylation levels in tumor cells over time under hypoxic conditions (0 h/6 h/12 h/24 h/48 h) were detected via an alkynyl-myristic acid probe (Ynmyr) and a pan-N-myristoylation antibody. **I** Changes in N-myristoylation, NMT1, and isoenzyme NMT2 protein levels after NMT1 was knocked down or overexpressed in tumor cells were detected. **J** Quantitative analysis of changes in N-myristoylation, NMT1, and isoenzyme NMT2 protein levels after NMT1 was knocked down or overexpressed. **K** Immunohistochemical analysis of 72 OSCC samples revealed significantly greater expression of NMT1 in tumor tissue than in adjacent normal tissue (*p* = 0.0439). **L** Statistical analysis of immunohistochemical staining results from 100 patient tumor pathology slices from our hospital, followed by plotting a Kaplan‒Meier survival curve. Patients were divided into high NMT1 expression and low NMT1 expression groups (*p* = 0.002174, hazard ratio (low/high) = 0.3767, 95% confidence interval: 0.1722–0.6792). **M** Kaplan‒Meier survival analysis after stratifying the TCGA cohort of 519 patients on the basis of high or low NMT1 expression levels. Hypoxic status was assessed via the "HALLMARK_HYPOXIA" gene set from the Molecular Signatures Database (MSigDB) on the GSEA website, which includes genes upregulated due to hypoxia in human cancers (*p* < 0.0001, hazard ratio (low/high) = 0.4986, 95% confidence interval: 0.3598–0.6908). **N** Immunofluorescence staining of tumor tissues to detect the tissue distribution of HIF1α and NMT1. **O** Quantitative analysis of the expression of HIF1α and NMT1 in the linear regions of the merged immunofluorescence images (r = 0.8213, *p* < 0.0001; scale bar: 30 μm). **P** Identification of a functional hypoxia response element in the NMT1 promoter region via ChIP‒qPCR technology. **Q** Further experiments confirmed that HIF1α activated promoter activity in a dose-dependent manner in SCC9 cell lines (*p* < 0.0001). **R** Further experiments confirmed that HIF1α activated promoter activity in a dose-dependent manner in SCC25 cell lines (*p* < 0.0001)

Transcriptomic analysis of head and neck squamous cell carcinoma (HNSCC) cell lines under normoxic and hypoxic conditions revealed significant activation of the membrane protein transport pathway. Gene Ontology (GO) enrichment analysis revealed that the "protein localization to membrane" pathway was specifically induced under hypoxia (enrichment score = 2.28, *p* = 5.87 × 10⁻^5^; Fig. 1A-B). Thirteen genes within this pathway were upregulated under hypoxic conditions (Fig. 1C), with NMT1 exhibiting the most significant differential expression (log2-fold change = 6.67, *p* = 6.9 × 10⁻^52^; Fig. 1D). NMT1, a key enzyme that catalyzes N-terminal protein myristoylation, regulates membrane protein localization and function through posttranslational modifications [21, 26].

Hypoxia regulates NMT1 expression in a time-dependent manner. Western blotting analysis revealed that with prolonged hypoxic exposure, the expression of hypoxia-inducible factor 1α (HIF1α) and NMT1 gradually increased and peaked after 24 h. In contrast, the isoenzyme NMT2 did not respond to hypoxia (Fig. 1E-F). Real-time quantitative PCR (qPCR) revealed that NMT1 mRNA levels were significantly elevated under hypoxia in three hypoxia-responsive squamous cell carcinoma cell lines (SCC9, SIHa, and KYSE-510) [10], whereas NMT2 levels remained unchanged (Fig. 1G and Fig S1A-B). These findings indicate that NMT1 upregulation enhances protein N-myristoylation in tumor cells [27]. As conventional Western blotting cannot detect lipid modifications, we employed a clickable alkyne-tagged myristic acid analog (YnMyr) to label tumor cells cultured under normoxic and hypoxic conditions (Fig S1C) [25]. YnMyr labeling revealed that N-myristoylation levels increased significantly with prolonged hypoxic exposure (Fig. 1H). Since knocking out NMT1 leads to the death of tumor cells, we designed three shRNAs (shNMT1-658, shNMT1-557, and shNMT1-267) that target the NMT1 gene and constructed lentiviral vectors. After the expression level of the NMT1 protein was detected, we determined that shNMT1-557 had the best knockdown effect (Fig S1D-E). To overexpress NMT1, we performed reverse synonymous mutation on its coding sequence (Fig S1D and F). Knockdown or overexpression of NMT1 confirmed that its expression directly regulates N-myristoylation levels in tumor cells (Fig. 1I). Notably, NMT2 expression remained unaffected, indicating no compensatory regulation by NMT2 in response to NMT1 alterations (Fig. 1J).

To validate the role of NMT1 in clinical samples, we analyzed tumor tissues and prognostic data from patients with HNSCC. We enrolled 72 patients with HNSCC who underwent surgical treatment and were followed up for 5 years (Supplementary Table 1). Immunohistochemical analysis of tumor tissues revealed that the expression level of NMT1 was greater than that in adjacent normal tissues (*p* = 0.0439; Fig. 1K). Patients were stratified into high NMT1 expression (IRS ≥ 1, 31 cases) and low NMT1 expression (IRS = 0, 41 cases) groups on the basis of the immunoreactive score (IRS) (Fig S1G). Survival analysis revealed that high NMT1 expression was associated with lower 5-year overall survival (Fig. 1L), a finding independently validated in The Cancer Genome Atlas (TCGA) cohort (Fig. 1M). Immunofluorescence staining of tumor sections revealed a positive correlation between NMT1 and HIF1α expression (r = 0.8213, *p* < 0.0001; Fig. 1N and O). Furthermore, we identified a functional hypoxia response element in the NMT1 promoter (Fig. 1P). Chromatin immunoprecipitation (ChIP)-qPCR experiments confirmed that HIF1α activated NMT1 promoter activity in a hypoxia-dependent manner (Fig. 1Q-R). These results demonstrate that hypoxia upregulates NMT1 expression, enhancing global protein N-myristoylation in tumor cells.

### NMT1-driven immune-suppressive microenvironment remodeling promotes tumor progression and poor prognosis

To investigate the specific mechanisms by which NMT1 regulates tumor progression and prognosis, we first analyzed the effects of NMT1 expression loss on the biological behavior of tumor cells via in vitro functional assays. CCK8 proliferation assays, flow cytometry-based apoptosis analysis, and cell cycle analysis revealed that compared with control cells, SCC9 cells with NMT1 knockdown (shNMT1) presented no significant differences in proliferative capacity, apoptosis rate, or cell cycle distribution (Fig S1H-J), suggesting that NMT1 deficiency does not directly affect the autonomous growth characteristics of tumor cells. Further in vivo functional validation demonstrated that xenograft tumors derived from subcutaneously inoculated shNMT1 and control cells in nude mice presented no significant differences in volume, weight, or growth curves (Fig S1K-P), which is consistent with the in vitro results. This paradoxical observation—where clinically high NMT1 expression is correlated with poor prognosis but lacks direct cell-autonomous tumor-promoting effects—prompts us to focus on noncell-autonomous mechanisms. Given the consistency of in vivo and in vitro functional data, we preliminarily infer that NMT1 may indirectly drive tumor progression by remodeling the tumor microenvironment (e.g., immunoregulatory networks), laying a logical foundation for subsequent exploration of its regulatory roles in the tumor microenvironment [28–30].

**Fig. 2:**
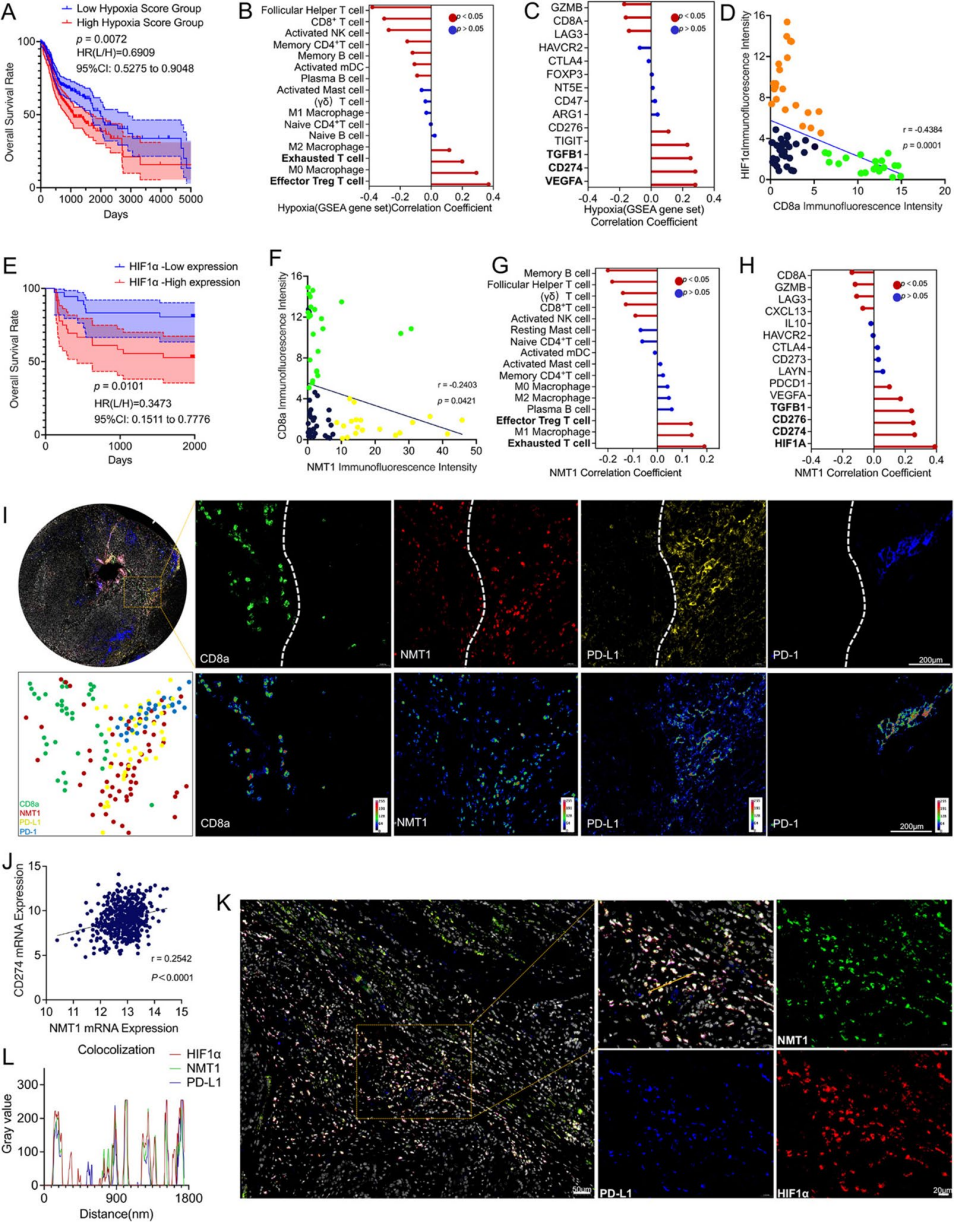
Exploring the role of NMT1 in tumor immune evasion and prognosis. **A** Bioinformatics analysis of TCGA data revealed that a high hypoxia score was associated with poor patient prognosis (*p* = 0.0072, hazard ratio (low/high) = 0.6909, 95% CI: 0.5275–0.9048). **B** Bioinformatics analysis of TCGA data revealed that the hypoxia score was positively correlated with effector Treg cells and exhausted T cells and negatively correlated with CD8⁺ T cells. **C** Bioinformatics analysis of TCGA data revealed that the hypoxia score was positively correlated with the expression of immunosuppressive molecules such as VEGFA, CD274 and TGFB1. **D** Analysis of 72 samples revealed that HIF1α was negatively correlated with CD8a (r = -0.4384, *p* = 0.0001). **E** Analysis of 72 samples revealed that high expression of HIF1α was associated with poor prognosis (*p* = 0.0101, hazard ratio (low/high) = 0.3473, 95% CI: 0.1511–0.7776). **F** Analysis of 72 samples indicated that NMT1 was negatively correlated with CD8A (r = 0.2403, *p* = 0.0421). **G** Bioinformatics analysis of TCGA data revealed that NMT1 was positively correlated with effector Treg cells and exhausted T cells and negatively correlated with cytotoxic T cells such as CD8⁺ T cells and γδT cells. **H** Bioinformatics analysis of TCGA data revealed that NMT1 was positively correlated with immunosuppressive molecules such as HIF1A, CD274, CD276 and TGFB1. **I** Immunofluorescence staining and regional expression intensity analysis were performed on tumor tissue slices from OSCC patients. The localization of CD8a, NMT1, PD-L1, and PD-1 within cellular or tissue regions was visually presented through different colored fluorescence channels. Below each fluorescence channel, an analysis of regional expression intensity was conducted. The phenotype map indicates the positional relationships of the four markers. (Scale bar: 200 μm). **J** Bioinformatics analysis of TCGA data revealed that NMT1 was positively correlated with PD-L1 (r = 0.2542, *P* < 0.0001). **K** Triple-staining of NMT1, HIF1α, and PD-L1 revealed obvious colocalization (scale bars: 50 μm and 20 μm). **L** Quantitative analysis of the coexpression relationships among NMT1, HIF1α, and PD-L1 (NMT1 vs. HIF1α: r = 0.898, *p* < 0.0001; PD-L1 vs. HIF1α: r = 0.933,* p* < p0.0001; NMT1 vs. PD-L1: r = 0.846,* p* < 0.0001)

To validate the role of hypoxia-induced NMT1 activation in immunosuppression, we utilized a hypoxia gene set to score the TCGA cohort. The results demonstrated that patients with higher hypoxia scores had significantly poorer prognoses (Fig. 2A). Leveraging the CIBERSORT deconvolution algorithm, we inferred the infiltration proportions of 16 immune cell subsets from TCGA transcriptomic data. To further explore the underlying mechanisms of hypoxia-mediated immune regulation, we selected representative immune activation- and immunosuppression-related molecules in the tumor microenvironment and analyzed their correlations with hypoxia scores and NMT1 expression. These analyses elucidated the roles of hypoxia and NMT1 in modulating the immune microenvironment from the perspectives of immune cell subset infiltration proportions and immunosuppressive molecules. Specifically, analysis revealed that hypoxia scores were positively correlated with the infiltration of immunosuppressive cell types, such as effector regulatory T cells (Treg cells) and exhausted CD8⁺ T cells (Fig. 2B), alongside the increased expression of immunosuppressive molecules, such as PD-L1 and vascular endothelial growth factor A (VEGFA) (Fig. 2C).

Immunofluorescence analysis of samples from a retrospective clinical study cohort (Fig S2A and B) revealed a significant negative correlation between the expression of HIF1α and the degree of CD8⁺ T-cell infiltration (r = -0.4384, *p* = 0.0001; Fig. 2D). Additionally, patients with high HIF1α expression experienced markedly shorter survival times (Fig. 2E). Further examination of NMT1 and CD8a expression in clinical samples (Fig S2C) revealed that NMT1 expression was also negatively correlated with CD8⁺ T-cell infiltration (r = -0.2403, *p* = 0.0421; Fig. 2F), which is consistent with the trend observed between HIF1α and CD8a. Systematic analysis of data from the TCGA database further revealed that NMT1 expression is closely associated with tumor immune evasion. Specifically, NMT1 expression levels were positively correlated with the infiltration of effector Treg cells and exhausted CD8⁺ T cells but negatively correlated with the infiltration of cytotoxic T cells, including CD8⁺ T cells and γδ T cells (Fig. 2G).

In-depth analysis of immunosuppression-related molecules revealed that NMT1 not only was strongly correlated with HIF1α but also positively correlated with the expression of genes such as PD-L1, CD276, TGFB1, and VEGFA (Fig. 2H). Staining analysis of phenotypic molecules related to the tumor immune microenvironment revealed that high NMT1 expression was associated with reduced CD8⁺ T-cell infiltration. Moreover, regions with high NMT1 expression were accompanied by elevated PD-1 and PD-L1 expression, suggesting the presence of an immunosuppressive microenvironment (Fig. 2I). Further analysis revealed a strong positive correlation between NMT1 and PD-L1 (r = 0.2542, *p* < 0.0001; Fig. 2J). Triple immunofluorescence staining further revealed spatial colocalization of HIF1α, NMT1, and PD-L1 within the hypoxic core regions of tumors (Fig. 2K-L), thereby revealing a comprehensive "hypoxia/HIF1α/NMT1/PD-L1" pathological axis. These findings confirm that NMT1 promotes tumor progression and leads to poor prognosis by modulating the immunosuppressive molecular network.

### Impact of hypoxia and NMT1 on PD-L1 membrane localization

**Fig. 3:**
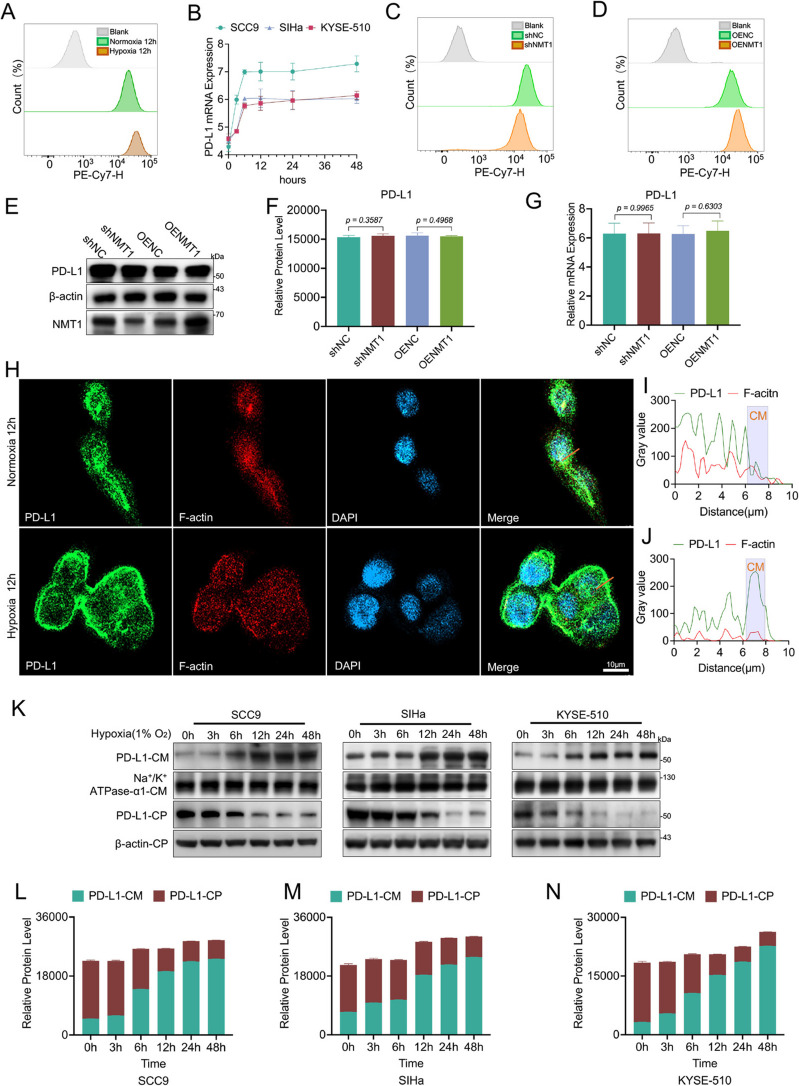
Influence of NMT1 on PD-L1 expression and localization in tumor cells. **A** Flow cytometry analysis was used to assess the expression level of PD-L1 on the cell membrane of the SCC9 cell line under both normoxic and hypoxic culture conditions. The data revealed an increased presence of PD-L1 on the cell membrane under hypoxic conditions. **B** RT‒qPCR was utilized to measure the mRNA expression levels of PD-L1 in the SCC9, SIHa and KYSE510 cell lines. The results demonstrated that hypoxia upregulated the transcriptional level of PD-L1. **C** Flow cytometry was conducted to observe changes in PD-L1 membrane expression in the SCC9 cell line following NMT1 knockdown. The knockdown of NMT1 resulted in a reduction in detectable PD-L1 on the cell membrane. **D** Flow cytometry analysis after NMT1 overexpression in the SCC9 cell line revealed an increase in PD-L1 membrane expression. **E** Western blot analysis was performed to evaluate total PD-L1 protein levels in the SCC9 cell line after NMT1 knockdown or overexpression. No significant alterations in PD-L1 expression were observed under these conditions. **F** Western blot analysis of total PD-L1 protein expression in the SCC9 cell line after NMT1 was knocked down or overexpressed. **G** RT‒qPCR analysis following NMT1 modulation in the SCC9 cell line confirmed that neither the knockdown nor the overexpression of NMT1 affected the mRNA level of PD-L1. **H** Immunofluorescence microscopy was used to visualize PD-L1 expression in the SCC9 cell line under normoxic versus hypoxic conditions (scale bar: 10 μm). **I**-**J** Quantitative analysis of the immunofluorescence images in panel G revealed a tendency for PD-L1 to aggregate toward the cell membrane under hypoxia (CM = cell membrane). **K** Western blotting of membrane and cytoplasmic protein fractions from the SCC9, SIHa, and KYSE510 cell lines exposed to hypoxia over time revealed an increase in PD-L1 in the membrane fraction and a corresponding decrease in the cytoplasmic fraction with prolonged hypoxic exposure (CM = cell membrane, CP = cytoplasm). **L** Quantitative analysis of PD-L1 content in the membrane protein fraction and cytoplasmic protein fraction of SCC9 cells (CM = cell membrane, CP = cytoplasm). **M** Quantitative analysis of PD-L1 content in the membrane protein fraction and cytoplasmic protein fraction of SiHa cells (CM = cell membrane, CP = cytoplasm). **N** Quantitative analysis of PD-L1 content in the membrane protein fraction and cytoplasmic protein fraction of KYSE510 cells (CM = cell membrane, CP = cytoplasm)

The regulatory effects of hypoxia and NMT1 on PD-L1 expression and localization were systematically investigated. Flow cytometry analysis of SCC9 cells under normoxic and hypoxic conditions revealed a significant hypoxia-induced increase in PD-L1 membrane levels (Fig. 3A; Fig S3A-B). Concurrent RT‒qPCR measurements confirmed elevated PD-L1 mRNA levels in SCC9, SIHa, and KYSE510 cells under hypoxia (Fig. 3B), demonstrating the dual role of hypoxia in enhancing PD-L1 transcription and membrane localization. Subsequent exploration of the involvement of NMT1 in PD-L1 regulation revealed that NMT1 knockdown reduced PD-L1 membrane levels (Fig. 3C; Fig S3C-D), whereas NMT1 overexpression increased PD-L1 levels (Fig. 3D; Fig S3E-F). Western blot (Fig. 3E-F) and RT-qPCR (Fig. 3G) analyses confirmed that these manipulations did not alter total PD-L1 protein or mRNA levels, confirming the specific role of NMT1 in PD-L1 membrane trafficking rather than overall expression.

Spatial immunofluorescence imaging (Fig. 3H) provided further evidence of hypoxia-dependent PD-L1 redistribution. Quantitative fluorescence intensity profiling revealed marked PD-L1 accumulation near the cell membrane (CM) under hypoxia, in contrast with the diffuse cytoplasmic distribution under normoxia (Fig. 3I). The isolation of plasma membrane proteins from SCC9, SIHa, and KYSE-510 cells (Fig. 3K) further corroborated these findings, revealing time-dependent PD-L1 enrichment in membrane fractions during prolonged hypoxia (Fig. 3L). These experiments collectively establish hypoxia as a critical driver of PD-L1 membrane accumulation in tumor cells.

**Fig. 4:**
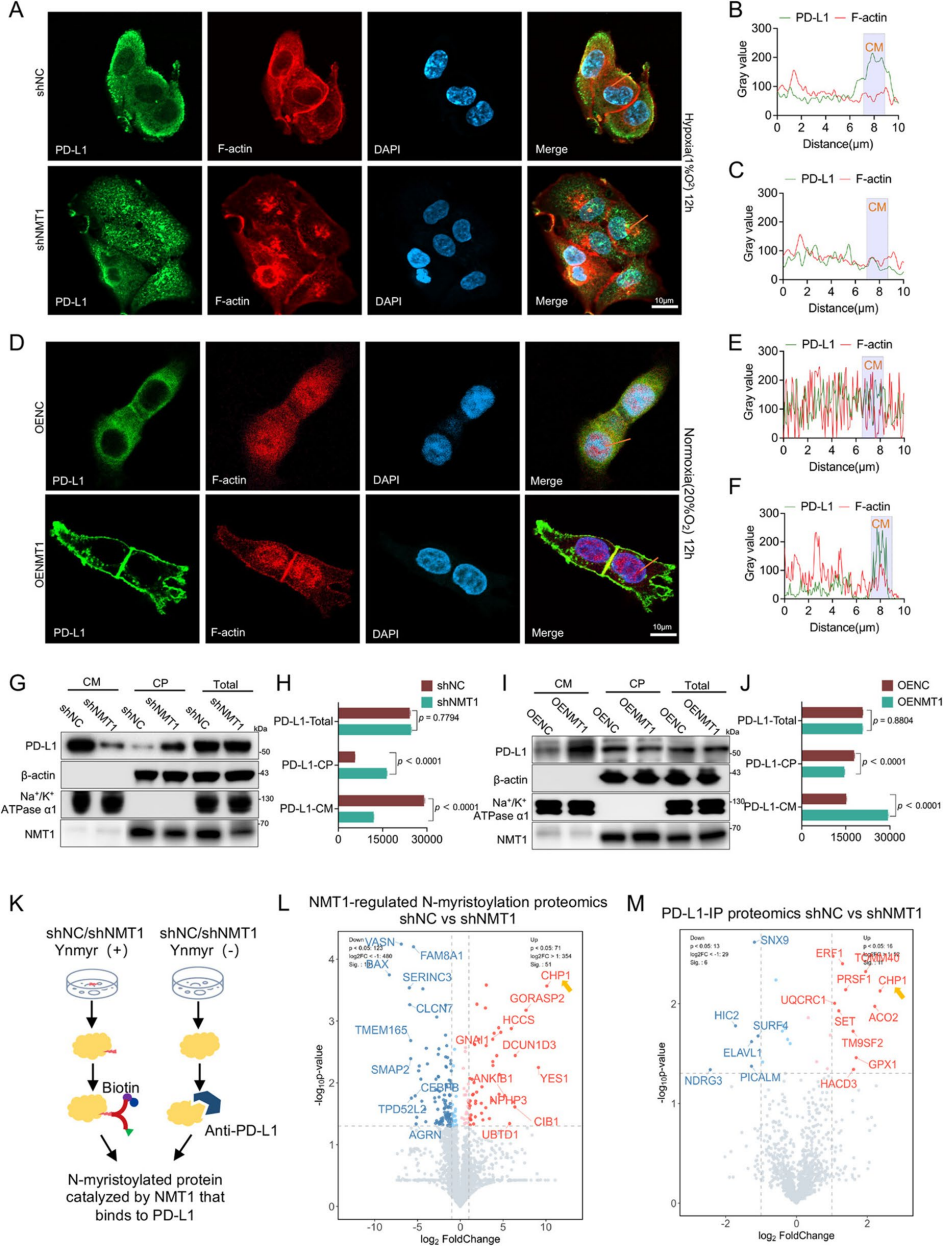
NMT1-regulated proteomics and the interaction between CHP1 and PD-L1. **A** Immunofluorescence was used to assess PD-L1 expression in the SCC9 cell line after NMT1 knockdown (scale bar: 10 μm). **B**-**C** The spatial distribution of PD-L1 in SCC9 cells with stable control lentivirus and SCC9 cells with NMT1 knockdown under hypoxic conditions was quantitatively evaluated. The quantitative region is the linear region of the merged image in panel A (CM = Cell Membrane). **D** Immunofluorescence was used to assess PD-L1 expression in the SCC9 cell line after NMT1 overexpression (scale bar: 10 μm). **E**-**F** Quantitative evaluation of the spatial distribution of PD-L1 in SCC9 cells with stable control lentivirus and SCC9 cells with NMT1 overexpression under hypoxic conditions. The quantitative region is the linear region of the merged image in panel A (CM = Cell Membrane). **G** Western blot analysis of membrane and cytoplasmic protein fractions from the SCC9 cell line with NMT1 knockdown revealed alterations in the subcellular localization of PD-L1 (CM = cell membrane, CP = cytoplasm). **H** Quantitative analysis of Western blots for PD-L1 in membrane and cytoplasmic protein fractions from the SCC9 cell line with NMT1 knockdown (CM = cell membrane, CP = cytoplasm). **I **Western blot analysis of membrane and cytoplasmic protein fractions from the SCC9 cell line with NMT1 overexpression revealed alterations in the localization of PD-L1 (CM = cell membrane, CP = cytoplasm). **J** Quantitative analysis of Western blots for PD-L1 in membrane and cytoplasmic protein fractions from the SCC9 cell line with NMT1 overexpression (CM = cell membrane, CP = cytoplasm). **K** In the SCC9 cell line, after NMT1 was knocked down, proteins whose modification levels changed were screened via Ynmyr labeling, and proteins whose binding to PD-L1 changed were screened via an anti-PD-L1 antibody. **L** Volcano plot of differentially expressed proteins identified via NMT1-related myristoylation proteomics. **M** Volcano plot of differentially expressed proteins identified via PD-L1 binding-related proteomics

Our findings indicate that hypoxia induces the translocation of PD-L1 to the cell membrane. To further explore this, we cultured SCC9 cells with NMT1 knockdown and control SCC9 cells under hypoxic conditions and assessed PD-L1 distribution on the plasma membrane via immunofluorescence. The results showed that in NMT1-knockdown SCC9 cells, PD-L1 was predominantly dispersed in the cytoplasm, reversing the hypoxia-induced enrichment of PD-L1 at the cell membrane (Fig. 4A). This suppression of PD-L1 membrane translocation was further corroborated by quantitative analysis of linear regions (Fig. 4B-C). Conversely, under normoxic conditions, NMT1 overexpression increased PD-L1 accumulation on the cell membrane (Fig. 4D-F). Notably, this rapid redistribution of PD-L1 to the plasma membrane cannot be fully explained by transcriptional upregulation alone. NMT1 may modify certain proteins, influencing the interaction between PD-L1 and transporter proteins or chaperones and thereby regulating its plasma membrane distribution [31]. Additionally, PD-L1 can undergo palmitoylation, a posttranslational fatty acid acylation modification, which may alter its conformation and facilitate its enrichment on the plasma membrane [32]. To confirm these observations, we isolated membrane and cytoplasmic proteins from SCC9 cells with NMT1 knockdown or overexpression (Fig. 4G-H). Western blot analysis revealed that PD-L1 levels in the membrane protein fraction decreased upon NMT1 knockdown but increased with NMT1 overexpression (Fig. 4I-J).

To determine whether PD-L1 is a direct substrate of NMT1, we used an anti-PD-L1 antibody to probe for YnMyr-labeled proteins. Notably, PD-L1 was not detected among the labeled proteins (Fig S3G). This result is attributed to the absence of an N-myristoylation recognition sequence (MGXXXX) at the N-terminus of the PD-L1 protein. These findings rule out direct N-myristoylation of PD-L1, suggesting an indirect mechanism, potentially involving NMT1-mediated modification of adaptor proteins, that facilitates PD-L1 enrichment on the cell membrane.

## NMT1 mediates the membrane translocation of PD-L1 through the myristoylation of CHP1

**Fig. 5:**
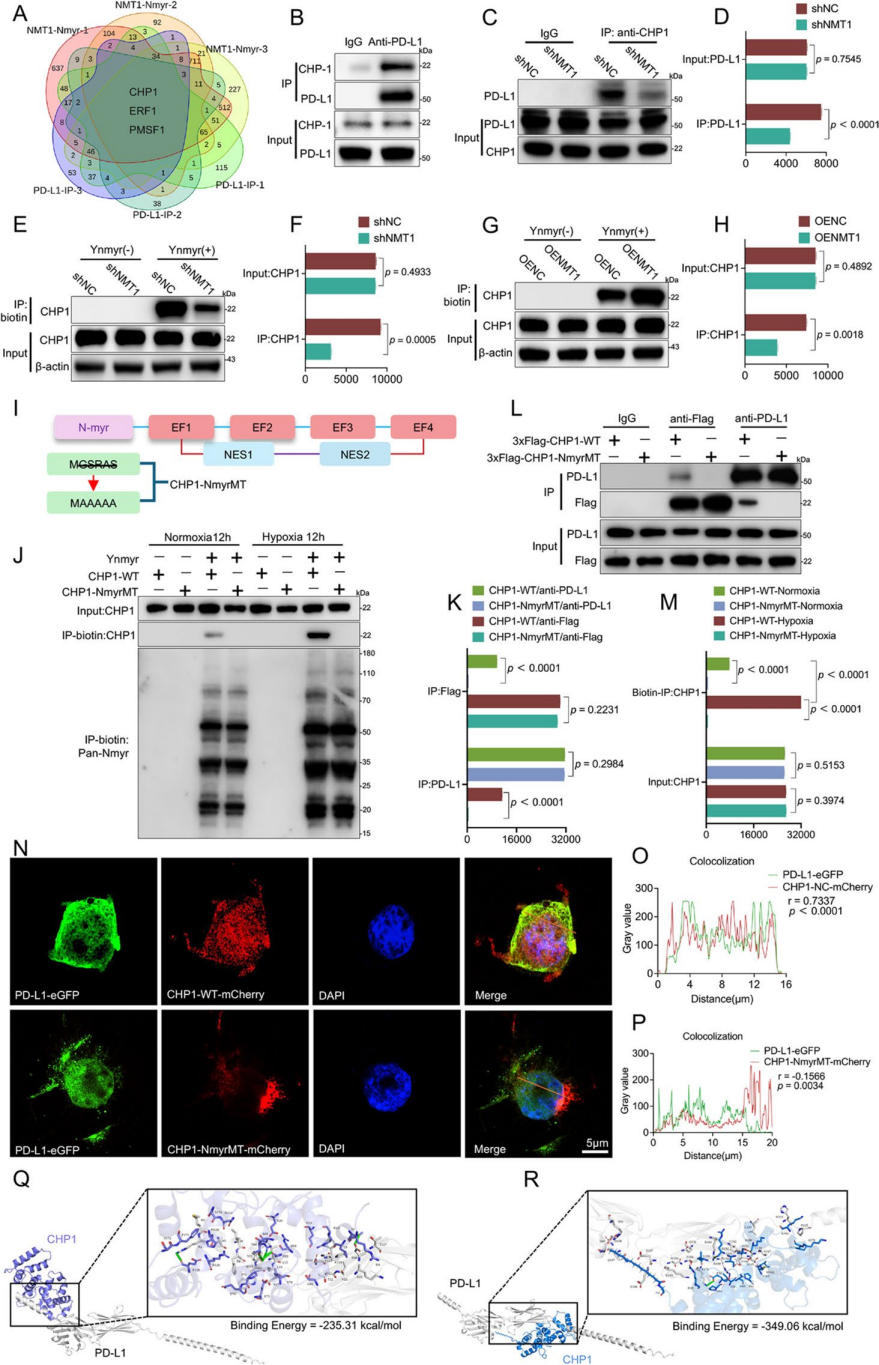
Distribution of CHP1 and PD-L1 and simulation of their interaction. **A** The differentially expressed genes screened by dual-omics were fitted, and a Venn diagram was used to display the genes with differential expression in all the samples. **B** The binding between CHP1 and PD-L1 was detected by co-IP, indicating direct binding between CHP1 and PD-L1. **C** In SCC9 cells, after NMT1 (shNMT1) was knocked down, co-IP was carried out with an anti-CHP1 antibody, and PD-L1 binding was detected via Western blotting. **D** Quantitative analysis of the binding of PD-L1 after performing co-IP with an anti-CHP1 antibody following NMT1 knockdown. **E** The modification level of CHP1 after NMT1 knockdown was detected via Ynmyr labeling. **F** Quantitative analysis of the modification level of CHP1 after NMT1 was knocked down. G. The modification level of CHP1 after overexpressing NMT1 was detected via Ynmyr labeling. **H** Quantitative analysis of the modification level of CHP1 after NMT1 was overexpressed. **I** Schematic diagram of the three types of domains of CHP1, including the EF-hand domain, the NES (nuclear export signal) domain, and the N-myr (N-myristoylation signal) domain. **J** Changes in the N-myristoylated modification level of CHP1 after mutation of the N-terminal myristoylated modification sequence of CHP1 under normoxic and hypoxic conditions were detected via Ynmyr labeling. The results revealed that hypoxia did not affect the expression level of CHP1 but altered its N-myristoylation level. Mutation of the N-terminal myristoylated modification sequence of CHP1 led to the inability of CHP1 to be labeled by Ynmyr; that is, it could not be modified by NMT1. **K** The resultsResults from the quantitative analysis of the immunoblotting bands of CHP1 in the IP group and the Input group in Panel J. **L** Mutated CHP1 with the Flag sequence and wild-type (WT) plasmids were transfected into tumor cells, and a Flag antibody and PD-L1 antibody were used to detect changes in the binding ability between CHP1 and PD-L1. **M** The resultsResults from the quantitative analysis of the immunoblotting bands of Flag and PD-L1 in the IP group shown in Panel L. **N** 293 T cells were transfected with CHP1-NC-mCherry/CHP1-NmyrMT-mCherry and PD-L1-eGFP plasmids. After plasmid transfection was complete, cell immunofluorescence staining was performed to observe changes in the distributions of PD-L1 and CHP1. PD-L1-eGFP is shown as green fluorescence, CHP1-NC-mCherry or CHP1-NmyrMT-mCherry is shown as red fluorescence, DAPI stains the nucleus and is shown as blue fluorescence, and merge is the merged image (scale bar: 5 μm). **O** Quantitative analysis of the colocalization of CHP1-NC-mCherry and PD-L1-eGFP in panel A. By analyzing the change in fluorescence intensity along a certain distance, the colocalization trend of the two proteins in the intracellular space is shown. The horizontal axis represents the distance (μm), and the vertical axis represents the gray value to evaluate the correlation of their distributions in the cell. **P** Quantitative analysis of the colocalization of CHP1-NmyrMT-mCherry and PD-L1-eGFP in panel A. Similarly, by analyzing the change in fluorescence intensity along a certain distance, the colocalization trend of the two proteins in the intracellular space is shown. The horizontal axis represents the distance (μm), and the vertical axis represents the gray value to evaluate the correlation of the distribution between the N-myristoylated CHP1 and PD-L1 in the cell. **Q**-**R** Simulation of the binding between nonmyristoylated and myristoylated CHP1 and PD-L1 proteins. The purple symbol represents the non-N-myristoylated CHP1 protein, the blue symbol represents the N-myristoylated CHP1 protein, and the white symbol represents the PD-L1 protein. The residues involved in the interaction are shown on the right, and the green lines represent the formed hydrogen bonds. Panel Q shows the simulation of the binding between nonmyristoylated CHP1 and PD-L1 with a binding energy of -235.31 kcal/mol. Panel R shows the simulation of the binding between myristoylated CHP1 and PD-L1 with a binding energy of - 349.06 kcal/mol, indicating that the myristoylation modification enhances the binding ability between CHP1 and PD-L1

We elucidated the molecular mechanisms underlying the interplay between NMT1 and PD-L1 membrane dynamics via a dual-omics approach (Fig. 4K). First, N-myristoylation studies were conducted in NMT1-knockdown cells via an alkyne-tagged myristic acid (YnMyr) probe. Proteomic analysis of N-myristoylated proteins revealed distinct differential expression patterns of NMT1-modified proteins (Fig. 4L). A clustering heatmap highlighted potential NMT1-modified proteins (Fig S3H). Gene Ontology (GO) enrichment analysis demonstrated that significant changes in myristoylation levels profoundly influenced protein transport regulation (Fig S3I). We subsequently mapped the PD-L1 interactome and its expression changes under NMT1-knockdown conditions (Figs. 4M and S3J). GO enrichment analysis indicated that these differentially expressed proteins were associated primarily with biological processes such as ubiquitin-like protein binding and calmodulin binding (Fig S3K). Cross-analysis of these datasets identified a candidate protein set, including CHP1, ERF1, and PMSF1, potentially linked to PD-L1 membrane transport (Fig. 5A). However, sequence analysis revealed that only CHP1 possesses an N-terminal myristoylation recognition sequence. Notably, previous studies have reported that CHP1 relies on N-terminal myristoylation to activate downstream GPAT4 [31].

Further coimmunoprecipitation (co-IP) experiments confirmed the interaction between CHP1 and PD-L1 (Fig. 5B). Additional analysis revealed that NMT1 knockdown significantly reduced the binding affinity between CHP1 and PD-L1, suggesting that NMT1 modulates this interaction by regulating CHP1 N-myristoylation (Fig. 5C). Critically, YnMyr labeling experiments verified that NMT1 knockdown or overexpression altered CHP1 N-terminal myristoylation levels (Fig. 5E).

The CHP1 protein structure comprises an N-terminal myristoylation site, four EF-hand domains, and two nuclear export signals. Mutation of the N-terminal myristoylation sequence MGSRAS to MAAAAA disrupted the myristoylation signal (Fig. 5I). Blocking CHP1’s N-terminal myristoylation prevented myristic acid attachment under both normoxic and hypoxic conditions. Moreover, in hypoxic tumor cells, the CHP1 N-myristoylation level increased without significant changes in its expression, which was consistent with the overall increase in the cellular N-myristoylation level (Fig. 5J). These findings underscore that CHP1 function is determined by posttranslational modification rather than transcriptional regulation.

To substantiate these observations, we transfected tumor cell lines with 3xFlag-CHP1-NmyrMT or control plasmids. Following mutation of CHP1’s N-terminal myristoylation site, Flag-tagged CHP1 failed to coprecipitate PD-L1, and FLAG antibodies similarly failed to pull down PD-L1 (Fig. 5L). This bidirectional evidence establishes that NMT1-mediated CHP1 N-myristoylation is essential for PD-L1 membrane transport and strongly supports a physical interaction between CHP1 and PD-L1 in an N-myristoylation-dependent manner.

Additionally, we investigated the impact of myristoylation signal alterations on coexpression patterns by cotransfecting CHP1-NmyrMT/WT-mCherry and PD-L1-eGFP plasmids into engineered 293T cells (Fig. 5N). In 293 T cells expressing wild-type CHP1-mCherry, PD-L1 localized to the cell membrane and colocalized with CHP1 (r = 0.7337, *p* < 0.0001; Fig. 5O). In contrast, cells with a mutated myristoylation signal presented distinct patterns of CHP1 and PD-L1 localization (r = -0.1566, *p* = 0.0034; Fig. 5P). Protein binding simulations corroborated these findings, showing that unmodified CHP1 had significantly reduced binding affinity to PD-L1, with intermolecular binding energies of -235.31 kcal/mol before modification and -349.06 kcal/mol after modification (Fig. 5Q-R). This enhanced affinity primarily results from N-terminal myristoylation, which markedly strengthens hydrophobic intermolecular interactions.

## Targeting NMT1 enhances PD-1 antibody efficacy and reshapes the antitumor immune response

**Fig. 6:**
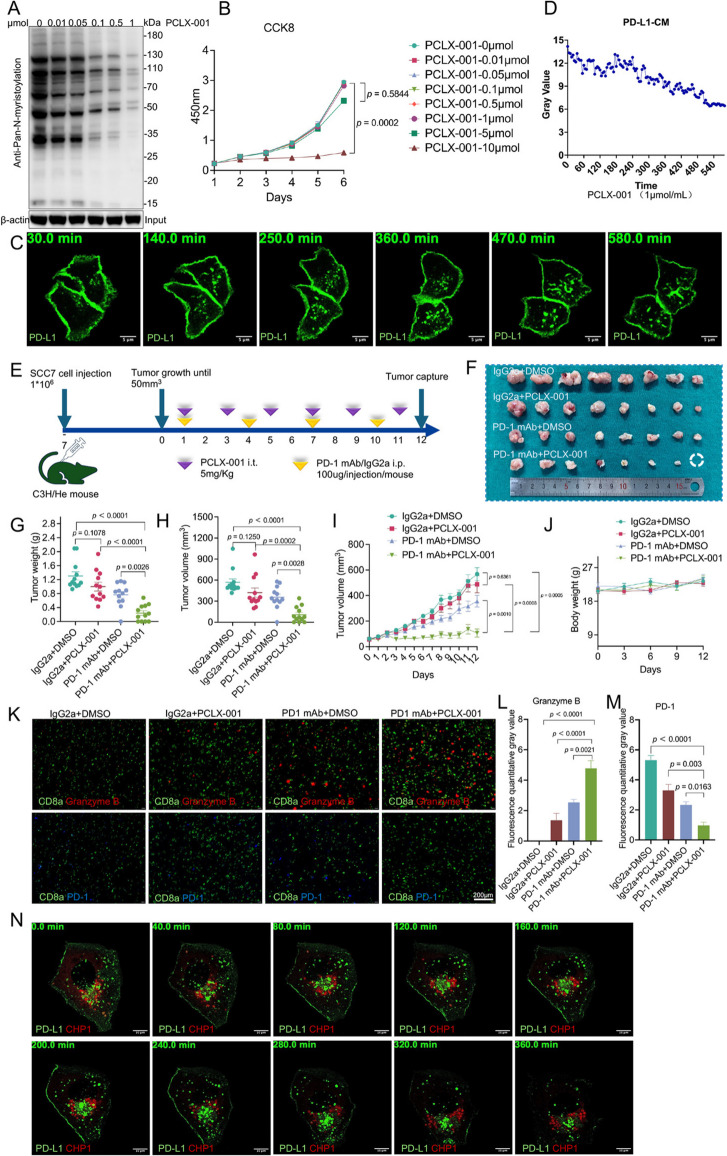
Impact of NMT1 inhibition and PD-1 blockade on tumor growth and the immune response in a subcutaneous tumor model. **A** Effects of different concentrations of PCLX-001 on N-myristoylation in tumor cells. Concentrations tested: 0/0.01/0.05/0.1/0.5/1 μmol (cell line: SCC7). **B** CCK8 assay to assess the impact of various concentrations of PCLX-001 on tumor cell proliferation. Concentrations tested: 0/0.01/0.05/0.1/0.5/1/5/10 μmol (cell line: SCC7). **C** Distribution of PD-L1 in SCC9 cells transfected with the PD-L1-eGFP plasmid after treatment with 1 μmol PCLX-001, as observed under an immunofluorescence microscope for 0–580 min (scale bar: 5 μm). **D** Quantitative analysis of PD-L1 expression intensity on the cell membrane from dynamic fluorescence videos at different time points after treatment (CM = cell membrane). **E** Subcutaneous tumor model in C3H/He mice with SCC7 cells. Treatment with the inhibitor and monoclonal antibodies started after tumor formation, with PD-1 mAb (100 μg/mouse) or control IgG2a administered intraperitoneally every three days and intratumoral injections of PCLX-001 (5 mg/kg) or DMSO every two days. The mice were sacrificed on day 12 when significant interindividual differences were observed. The tumor volume was measured daily, and the mouse weight was measured every three days. The final tumor weight and volume were measured postmortem. **F** Tumors were excised on day 12 posttumor formation and photographed to compare tumor growth. **G** Tumor weights were compared after excision. **H** Tumor volumes were compared after excision. **I** Daily tumor volume growth curves after tumor formation. **J** Mouse weight gain curves posttumor formation. **K** Immunofluorescence staining of tumor sections for CD8, PD-1, and Granzyme B (scale bar: 80 μm). **L** Quantitative analysis of Granzyme B fluorescence intensity. **M** Quantitative analysis of PD-1 fluorescence intensity. **N** Distribution of PD-L1 and CHP1 in SCC9 cells transfected with PD-L1-eGFP and CHP1-mCherry plasmids after treatment with 1 μmol PCLX-001, as observed via immunofluorescence microscopy over a time frame of 0–360 min (scale bar: 10 μm)

To evaluate the therapeutic potential of the NMT1 inhibitor PCLX-001, its effects on N-myristoylation and cell proliferation in SCC9 cells were investigated. At a concentration of 0.1 μmol/L, PCLX-001 significantly reduced the overall N-myristoylation level, demonstrating its ability to selectively target the catalytic function of NMT1 at low doses without relying on cytotoxicity (Fig. 6A). However, the inhibition of cell proliferation required a higher concentration, exceeding 10 μmol/L, indicating a clear distinction between its enzymatic inhibition and cytotoxic effects (Fig. 6B). This selective action at the subtoxic level provides a foundation for its therapeutic application.

Further exploration of the mechanism of PCLX-001 involved the use of PD-L1-eGFP fluorescence tracking to observe changes in PD-L1 distribution in treated SCC9 cells. With the addition of 1 μmol of PCLX-001, PD-L1 was notably retained in the cytoplasm, and after 580 min, the fluorescence intensity on the cell membrane significantly decreased, indicating a time-dependent effect (Fig. 6C and Mov 1). Experiments showing reduced colocalization of PD-L1 with CHP1-mCherry over time further confirmed that PCLX-001 disrupted the myristoylation of CHP1, thereby impeding PD-L1 membrane localization (Fig. 6N and Mov 2). This dynamic reversal highlights the potential of PCLX-001 to modulate PD-L1 localization.

In a preclinical study using a subcutaneous tumor model in C3H/He mice, the synergistic effect of PCLX-001 and a PD-1 monoclonal antibody was explored (Fig. 6E). The administration of PD-1 antibody or control IgG2a every three days, along with intratumoral injections of PCLX-001 or DMSO every two days, produced significant results (Fig. 6F). Importantly, no significant weight loss was observed in the mice, indicating good treatment tolerability (Fig. 6J). These findings underscore the enhanced efficacy of combining PCLX-001 with PD-1 blockade therapy.

The impact of this combination therapy on the tumor immune microenvironment was assessed via immunofluorescence staining of tumor sections for CD8, PD-1, and Granzyme B (Fig. 6K). In the combination treatment group, there was a marked increase in the expression of Granzyme B, a marker of cytotoxic T-cell activity, indicating restored effector function (Fig. 6L). Moreover, PD-1 expression was significantly lower than that in the PD-1 monotherapy group, suggesting alleviation of T-cell exhaustion (Fig. 6M). This reshaping of the immune landscape indicates a stronger antitumor immune response.

The precision of PCLX-001 in targeting NMT1 at subtoxic concentrations (0.1-1 μmol/L) ensures minimal off-target effects, enhancing its therapeutic profile. By blocking PD-L1 membrane localization, PCLX-001 prevented reactivation of the PD-1/PD-L1 axis, complementing the signal inhibition by the PD-1 antibody with spatial blockade (Fig. 6C, N and Mov 2). This dual mechanism not only enhances the immediate antitumor effects but also potentially fosters the formation of immune memory, as evidenced by the significant increase in effector T-cell function and infiltration. These findings highlight the critical role of N-myristoylation in regulating PD-L1 membrane localization and function under hypoxic conditions, providing new mechanistic insights into tumor immune evasion.

## Discussion

In the treatment of head and neck squamous cell carcinoma (HNSCC), the lower-than-ideal response rates to immunotherapy can be attributed, in part, to immunosuppressive mechanisms triggered by the highly hypoxic tumor microenvironment (TME) [1, 33–36]. This study revealed that NMT1 serves as a core regulator of spatial proteome remodeling under hypoxic stress, providing new perspectives for the exploration of such regulatory mechanisms. In contrast to the classical theory reported by Semenza et al. that hypoxia affects mainly transcription factors [35], this study expands the scope of hypoxia regulation to include protein membrane anchoring, a crucial step in oncogenic signal transduction.

In terms of mechanism innovation, the conventional understanding is that hypoxia promotes immune evasion primarily by upregulating PD-L1 transcription in cancer cells via HIF1α [12, 35–37]. However, this theory does not fully account for the rapid accumulation of PD-L1 in the cancer cell membrane. This study reveals that hypoxia controls PD-L1 function through a dual mechanism. On the one hand, there is transcriptional regulation, where hypoxia leads to an increase in PD-L1 mRNA levels, whereas spatial regulation occurs where NMT1 mediates the myristoylation of CHP1, resulting in the formation of a "CHP1‒PD-L1" complex in which excessive anchors PD-L1 to the cell membrane. This discovery is significant, as it shifts the focus from the transcription-centric paradigm of hypoxia research to the dynamic regulation of posttranslational modifications (PTMs) in tumor immunology evasion [38, 39]. Further investigation revealed that CHP1, which serves as a linker molecule, depends on N-terminal myristoylation for PD-L1 membrane localization. Structural simulations indicated that myristoylation strengthens the hydrophobic interactions between CHP1 and PD-L1, increasing their binding affinity by 50%. This mechanism provides a clear explanation for how PD-L1, which does not possess a myristoylation motif, efficiently localizes to the membrane under hypoxic conditions, offering a novel perspective on the membrane localization of proteins without classical modification motifs [19, 40–43].

For therapeutic translation, this study suggests a cancer therapy strategy through a combined treatment approach of "spatial blockade + signaling inhibition." Specifically, the PD-1 antibody targets the PD-1 receptor on CD8^+^ T cells, blocking the immunosuppressive signal mediated by the PD-1/PD-L1 interaction and thereby reversing T-cell exhaustion. Concurrently, the NMT1 inhibitor PCLX-001, at a concentration of 0.1 μmol/L, reduces PD-L1 membrane localization on tumor cells by inhibiting CHP1 myristoylation, decreasing PD-L1 availability to interact with PD-1 on T cells [44]. This dual mechanism—signal inhibition of CD8^+^ T cells and spatial blockade of tumor cells—works synergistically to maximize the elimination of PD-1/PD-L1-mediated immune evasion [45]. The results from the animal experiments were striking; combination therapy significantly inhibited tumor growth and reshaped the immune microenvironment with CD8^+^ T-cell infiltration [46]. Notably, PCLX-001 at subtoxic concentrations (0.1-1 μmol/L) selectively inhibited NMT1, avoiding the nonspecific toxicity of traditional chemotherapeutic drugs. This feature makes PCLX-001 a promising immunotherapeutic sensitizer and offers new hope for therapeutic advancements [47].

However, recognizing the clinical significance and limitations of this study is crucial. Clinically, high NMT1 expression is significantly associated with poor prognosis in patients with HNSCC, and NMT1 colocalizes with HIF1α/PD-L1, suggesting its potential as a combined biomarker. Additionally, the synergistic effect demonstrated by combining PCLX-001 with an anti-PD-L1 antibody provides a robust theoretical basis for clinical trials, especially in patients resistant to high PD-L1 membrane expression [48, 49]. However, these limitations must be considered. The study primarily utilized cell lines and mouse models; future validation in patient-derived organoid (PDO) or humanized mouse models is needed to ensure the reliability and clinical relevance of the findings [10]. In terms of mechanism, whether the CHP1-PD-L1 complex recruits other auxiliary proteins, such as membrane scaffold proteins, remains to be explored. Furthermore, although similar phenomena have been observed in cervical cancer (SIHa) and esophageal cancer (KYSE510) cells, broader validation across various cancer types is necessary to confirm the universality of this mechanism [50].

Clearly, clear research directions have emerged. For clinical translation, phase I clinical trials combining PCLX-001 with PD-1 antibodies should be pursued, with a focus on assessing their therapeutic effect on patients with PD-L1 membrane positivity [51]. In terms of mechanism expansion, a deeper analysis of the spatiotemporal dynamics of CHP1 myristoylation and its impact on immunological synapse formation is warranted to enhance the mechanistic understanding. For combination strategies, exploring the synergistic effects of NMT1 inhibitors with oncolytic viruses or CAR-T-cell therapy could be promising, with the aim of further activating antitumor immune responses and broadening the treatment landscape for cancer [52, 53].

In summary, this study successfully revealed a novel mechanism by which hypoxia regulates PD-L1 membrane localization via the NMT1-CHP1 axis and validated the feasibility of enhancing PD-1 blockade treatment efficacy by targeting this pathway. By combining an anti-PD-1 antibody to block immunosuppressive signaling on CD8^+^ T cells with PCLX-001 to reduce PD-L1 membrane localization on tumor cells, this approach maximizes the disruption of PD-1/PD-L1-mediated immune evasion. This discovery not only deepens the understanding of dynamic regulation within the tumor immune microenvironment but also offers an innovative approach to circumvent resistance to ICI treatment. Future research should focus on clinical translation, actively promoting precision immunotherapy on the basis of protein spatial regulation, to offer a more effective strategy for patients with cancers under hypoxic conditions.

## Conclusions

**Fig. 7:**
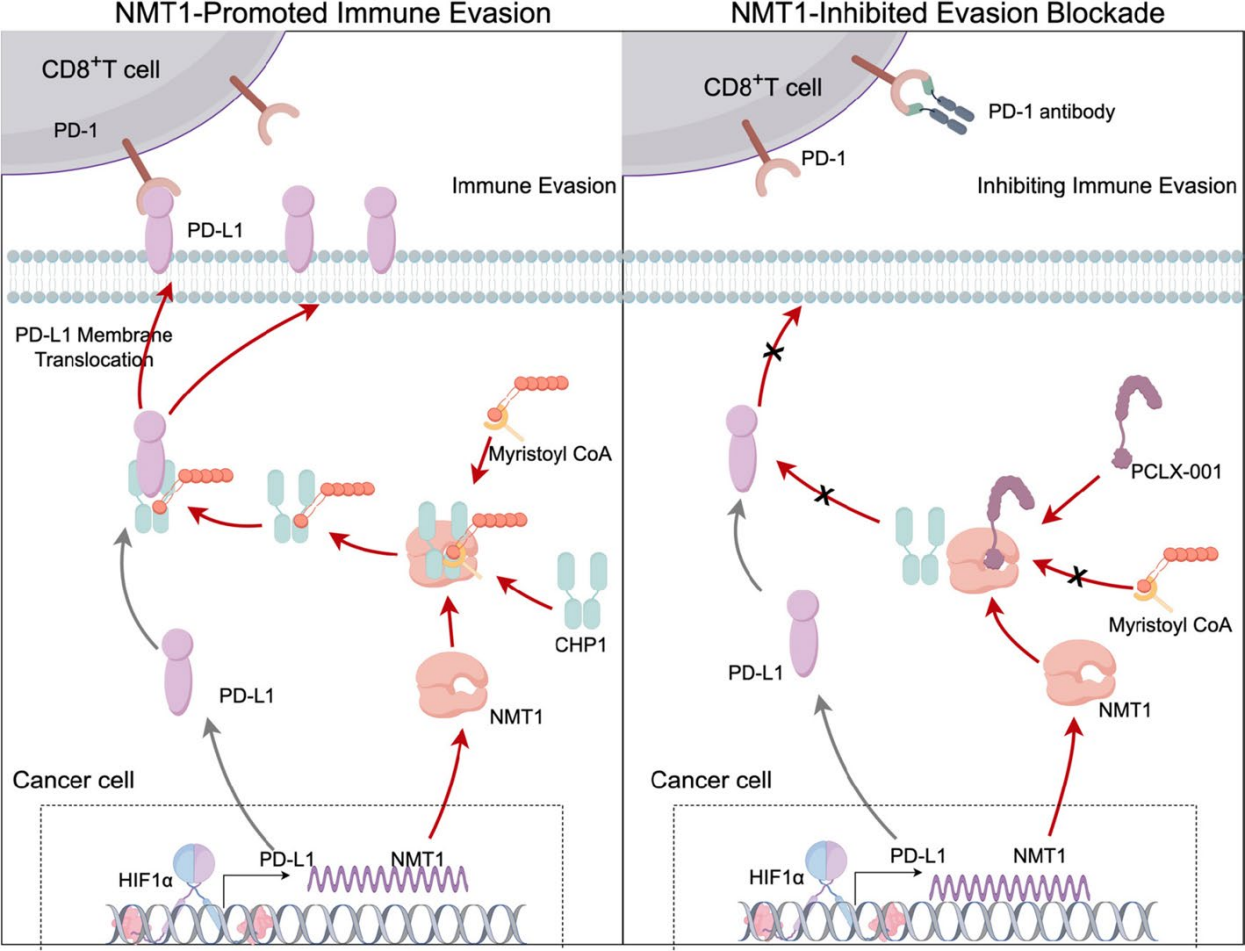
NMT1-mediated immune evasion mechanism in cancer cells and its inhibition principle. NMT1 Promotes Immune Evasion: Under hypoxic conditions, the transcription factor HIF1α in cancer cells upregulates the expression of both PD-L1 and NMT1. NMT1, in collaboration with the CHP1 protein and myristoyl CoA, catalyzes the myristoylation of CHP1. This posttranslational modification enhances the translocation of PD-L1 to the cancer cell membrane. Once expressed on the cell surface, PD-L1 interacts with PD-1 receptors on CD8^+^ T cells, leading to the suppression of T-cell-mediated immune responses and enabling cancer cells to evade immune detection and destruction. NMT1-Inhibited Evasion Blockade: The pharmacological agent PCLX-001 inhibits NMT1 activity, thereby preventing the myristoylation of PD-L1 and its subsequent trafficking to the cancer cell membrane. Simultaneously, an anti-PD-1 antibody binds to PD-1 on CD8^+^ T cells, disrupting the interaction between PD-1 and PD-L1. This dual therapeutic strategy—combining NMT1 inhibition with PD-1/PD-L1 pathway blockade—effectively abrogates the immune evasion mechanism, thereby restoring the cytotoxic capacity of T cells to target and eliminate cancer cells

In conclusion, our study demonstrates that the PD-1 antibody and the NMT1 inhibitor PCLX-001 employ complementary mechanisms to counteract the PD-1/PD-L1-mediated immune evasion. The PD-1 antibody specifically targets the PD-1 receptor on CD8^+^ T cells, effectively blocking the immunosuppressive signals transmitted through the PD-1/PD-L1 interaction. Meanwhile, PCLX-001 reduces the membrane localization of PD-L1 on tumor cells, thereby weakening the interaction between PD-L1 on tumor cells and PD-1 on T cells. The synergistic action of these two approaches, involving signal inhibition of CD8^+^ T cells and spatial blockade of tumor cells, maximizes the disruption of the immune evasion pathway. These findings not only deepen our understanding of the regulatory mechanisms underlying tumor immune escape but also provide solid theoretical evidence for the translational application of the combination of NMT1 inhibition and PD-1 blockade in cancer therapy. This combined strategy represents a promising approach to enhance antitumor immunity, offering new hope for cancer treatment and future research directions (Fig. 7).

## Supplementary Information

Supplementary Material 1.

Supplementary Material 2.

Supplementary Material 3.

Supplementary Material 4.

Supplementary Material 5.

Supplementary Material 6: Figure S1. A. Expression of NMT1 and NMT2 at the mRNA level in the KYSE-510 cell line under hypoxic conditions. B. Expression of NMT1 and NMT2 at the mRNA level in the SiHa cell line under hypoxic conditions. C. Schematic diagram of the functional assessment using an alkyne-tagged myristic acid (YnMyr) probe. D. Verification of NMT1 knockdown in SCC9 and CAL27 cell lines via lentiviral transfection and verification of synonymous mutation overexpression of NMT1 in the SCC9 cell line via lentiviral transfection. E. Quantitative analysis of NMT1 knockdown levels in SCC9 and CAL27 cell lines. F. Quantitative analysis of NMT1 overexpression in the SCC9 cell line. G. Example of high NMT1 expression detected via immunohistochemical staining of tumor tissue slices with an IRS ≥ 1 (scale bars: 2 mm and 400 μm). H: Detection of apoptosis in shNMT1-SCC9 and shNC-SCC9 cells. I: Cell cycle analysis of shNMT1-SCC9 and shNC-SCC9 cells. J. CCK8 assay was used to evaluate the proliferation capacity of shNMT1-SCC9 and shNC-SCC9 cells (*P* value = 0.8808). K. Subcutaneous injection of lentivirus-transfected shNMT1-SCC9 cells and control lentivirus-transfected SCC9 cells into the backs of nude mice. Observations began after tumor formation, and the mice were sacrificed 15 days after injection, which was 8 days after tumor formation. L. Tumor images showing the tumorigenic effect. M. Final tumor weight. N. Final tumor volume. O. Tumor growth volume. O. Tumor growth volume.

Supplementary Material 7: Figure S2. A. Thumbnail images of immunohistochemical staining of 72 OSCC tumor tissues. B. Schematic representation of the relationship between HIF1α and CD8a expression via immunohistochemical staining of 72 OSCC tumor tissues (scale bars: 500 μm, 200 μm, and 1 cm). C. Schematic representation of the relationship between NMT1 and CD8a expression via immunohistochemical staining of 72 OSCC tumor tissues (scale bars: 500 μm, 200 μm, and 1 cm).

Supplementary Material 8: Figure S3. A. Cell population with PD-L1 cell membrane staining under hypoxic and normoxic conditions; cell line: SCC9. B. Expression intensity of PD-L1 on the cell membrane under hypoxic and normoxic conditions; cell line: SCC9. C. Cell population with PD-L1 cell membrane staining after NMT1 knockdown; cell line: SCC9. D. Expression intensity of PD-L1 on the cell membrane after NMT1 knockdown; cell line: SCC9. E. Cell population with PD-L1 cell membrane staining after NMT1 overexpression; cell line: SCC9. F. Expression intensity of PD-L1 on the cell membrane after NMT1 overexpression; cell line: SCC9. G. Subsequent detection with an anti-PD-L1 antibody on proteins transferred to a PVDF membrane from the myristic acid probe labeling experiment did not reveal PD-L1 among the N-myristoylated proteins, indicating that PD-L1 is not directly modified by myristoylation. H. Heatmap of differentially expressed proteins identified via NMT1-related myristoylation proteomics. I. Pathway enrichment map of differentially expressed proteins identified via NMT1-related myristoylation proteomics. J. Heatmap of differentially expressed proteins identified via PD-L1 binding-related proteomics. H. Fitting results of the two-omics data. K. Pathway enrichment map of differentially expressed proteins identified via PD-L1 binding-related proteomics.

Supplementary Material 9.

Supplementary Material 10.

Supplementary Material 11.

Supplementary Material 12.

Supplementary Material 13.

## Data Availability

The mass spectrometry proteomics data were deposited in the ProteomeXchange Consortium (https://proteomecentral.proteomexchange.org) via the iProX partner repository [54, 55] with the dataset identifiers PXD057175 and PXD057274. Hypoxia transcriptomics data have been uploaded to the GEO database (https://www.ncbi.nlm.nih.gov/geo/query/acc.cgi?acc=GSE280308), with the dataset identifier GSE280308. The clinical data from the retrospective clinical study and the intensity of NMT1 immunohistochemical staining are presented in Supplementary Table 1. Three shRNA sequences targeting NMT1, the overexpression sequence with reverse synonymous mutation, and the sequences of the 3xFlag-CHP1-NmyrMT, mCherry-CHP1-NmyrMT, and PD-L1-eGFP plasmids, and the primers for the hypoxia-responsive elements in the promoter region of NMT1 can be found in Supplementary Table 2. The data on the expression intensities of triple immunofluorescence staining for HIF1α, NMT1, and CD8a can be found in Supplementary data 1. The data related to the animal experiments are provided in Supplementary Data 2. The quantitative Western blotting, fluorescence quantification and statistical data on animal experiments are presented in Supplementary Data 3.

## Abbreviations

CAR-T: Chimeric antigen receptor T-cell
ChIP: Chromatin immunoprecipitation
CHP1: Calcineurin B homologous protein 1
CM: Cell membrane
Co-IP: Coimmunoprecipitation
GO: Gene ontology
HIF1α: Hypoxia-inducible factor-1α
HNSCC: Head and neck squamous cell carcinoma
ICB: Immune checkpoint blockade
ICI: Immune checkpoint inhibitor
IRS: Immunoreactive score
NMT1: N-myristoyltransferase 1
NmyrMT: N-myristoylation mutant
PD-L1: Programmed death-ligand 1
PDO: Patient-derived organoid
RT-qPCR: Real-time quantitative polymerase chain reaction
TCGA: The Cancer Genome Atlas
Treg: Regulatory T cells
VEGFA: Vascular endothelial growth factor A
