# Correction: Elucidating the role of N‑myristoylation in the excessive membrane localization of PD‑L1 in hypoxic cancers and developing a novel NMT1 inhibitor for combination with immune checkpoint blockade therapy

**DOI:** 10.1186/s13046-025-03533-1

**Published:** 2025-09-04

**Authors:** Haoming Zhao, Zhen Zhang, Chaojun Zhang, Hexin Ma, Qingqing Wan, Xinran Zhao, Xu Wang, Ming Yan, Haiyan Guo, Jianjun Zhang, Wantao Chen

**Affiliations:** 1 Department of Oral and Maxillofacial-Head and Neck Oncology, Shanghai Ninth People’s Hospital, Shanghai Jiao Tong University School of Medicine, College of Stomatology, Shanghai Jiao Tong University, Shanghai, China; 2 Shanghai Key Laboratory of Stomatology, National Center for Stomatology, National Clinical Research Center for Oral Diseases, Shanghai Research Institute of Stomatology, Shanghai, China; 3 Digital Diagnosis and Treatment Innovation Center for Cancer, Institute of Translational Medicine, Shanghai Jiao Tong University, Shanghai, China; 4 Department of Clinical Laboratory, Shanghai Ninth People’s Hospital, Shanghai Jiao Tong University School of Medicine, College of Stomatology, Shanghai Jiao Tong University, Shanghai, China

Correction: *J Exp Clin Cancer Res* **44**, 181 (2025)

10.1186/s13046-025-03438-z

Following publication of the original article [1], the authors identified few minor errors in the published version of their article. They are as follows:

1. Supplementary Material 10 – incorrect efile was uploaded—MOV1 instead of MOV2
2. Under Materials and Methods:“anti-PD-L1 Ab (#BE0101; 25 mg; Bioxcell)” should be corrected to “anti-PD-1 Ab (#BE0146; 25 mg; Bioxcell)”
3. Figure 7 caption – “catalyzes the myristoylation of PD-L1” should be changed to “catalyzes the myristoylation of CHP1.”

The corrections do not compromise the validity of the conclusions and the overall content of the article. The original article [1] has been corrected.
