# Supplementary material for: Elucidating the role of N-myristoylation in the excessive membrane localization of PD-L1 in hypoxic cancers and developing a novel NMT1 inhibitor for combination with immune checkpoint blockade therapy
Source: J Exp Clin Cancer Res. 2025 Jul 2;44:181. doi: 10.1186/s13046-025-03438-z (PMC12219335; doi:10.1186/s13046-025-03438-z)
Supplement: Supplementary file 1 — Supplementary Material 1. [file 13046_2025_3438_MOESM1_ESM.docx]

| Pathological number | Tumor staging | Survival duration(Days) | NMT1 high expression(1)/NMT1 low expression(0) | Hscore-Paracancerous tissue | Hscore-Cancer nest | IRS-Paracancerous tissue | IRS-Cancer nest |
| --- | --- | --- | --- | --- | --- | --- | --- |
| 2018-04212 | I | 1933 | 1 | 8.86 | 34.84 | 1 | 2 |
| 2018-04213 | I | 1933 | 1 | 0.86 | 10.68 | 0 | 1 |
| 2018-04214 | I | 1933 | 0 | 0 | 3.03 | 0 | 0 |
| 2018-04220 | II | 1606 | 1 | 0 | 14.28 | 0 | 2 |
| 2018-04286 | III | 210 | 0 | 1.07 | 2.02 | 0 | 0 |
| 2018-04291 | II | 1932 | 0 | 0 | 2.68 | 0 | 0 |
| 2018-04293 | II | 1932 | 1 | 0 | 11.79 | 0 | 1 |
| 2018-04369 | III | 463 | 0 | 0.19 | 0.46 | 0 | 0 |
| 2018-04484 | II | 1929 | 0 | 0.05 | 0.15 | 0 | 0 |
| 2018-04486 | II | 55 | 0 | 0.3 | 0.99 | 0 | 0 |
| 2018-04535 | II | 221 | 1 | 4.46 | 13.01 | 0 | 1 |
| 2018-04536 | II | 215 | 1 | 5.26 | 21.8 | 0 | 2 |
| 2018-04537 | I | 1929 | 0 | 0.08 | 0.47 | 0 | 0 |
| 2018-04621 | II | 96 | 1 | 11.06 | 26.06 | 1 | 2 |
| 2018-04701 | II | 1925 | 0 | 0.09 | 0.22 | 0 | 0 |
| 2018-04803 | II | 1923 | 1 | 1.16 | 6.58 | 0 | 1 |
| 2018-04805 | I | 1925 | 0 | 0 | 0.14 | 0 | 0 |
| 2018-04813 | I | 1923 | 0 | 0.15 | 0.3 | 0 | 0 |
| 2018-04814 | II | 1923 | 0 | 0.23 | 0.7 | 0 | 0 |
| 2018-04816 | II | 1923 | 0 | 0.14 | 0.55 | 0 | 0 |
| 2018-04817 | II | 1923 | 0 | 0.14 | 0.62 | 0 | 0 |
| 2018-04872 | I | 1921 | 0 | 1.37 | 2.72 | 0 | 0 |
| 2018-04875 | II | 494 | 0 | 0.41 | 1.21 | 0 | 0 |
| 2018-04878 | II | 1921 | 0 | 0 | 0.35 | 0 | 0 |
| 2018-04963 | II | 190 | 1 | 3.49 | 14.68 | 0 | 1 |
| 2018-05041 | II | 164 | 1 | 5.1 | 34.39 | 0 | 2 |
| 2018-05140 | II | 139 | 1 | 44.03 | 57.64 | 4 | 5 |
| 2018-05207 | I | 992 | 0 | 0.16 | 1.4 | 0 | 0 |
| 2018-05209 | II | 311 | 1 | 8.16 | 14.55 | 1 | 1 |
| 2018-05283 | II | 1564 | 1 | 152.77 | 217.42 | 12 | 20 |
| 2018-05412 | I | 1909 | 0 | 2.74 | 5.24 | 0 | 0 |
| 2018-05415 | II | 1909 | 0 | 0.09 | 0.31 | 0 | 0 |
| 2018-05474 | II | 1078 | 0 | 0.15 | 0.18 | 0 | 0 |
| 2018-05559 | II | 1905 | 0 | 0.3 | 0.87 | 0 | 0 |
| 2018-05568 | II | 545 | 1 | 5.24 | 16.26 | 0 | 2 |
| 2018-05570 | II | 1905 | 0 | 0.5 | 2.26 | 0 | 0 |
| 2018-05644 | II | 1903 | 1 | 0.46 | 8.66 | 0 | 1 |
| 2018-05784 | II | 663 | 0 | 0.3 | 2.18 | 0 | 0 |
| 2018-05847 | II | 1897 | 1 | 1.5 | 41.48 | 0 | 2 |
| 2018-05850 | II | 1900 | 1 | 0.43 | 12.48 | 0 | 1 |
| 2018-05924 | II | 283 | 1 | 13.29 | 47.35 | 0 | 4 |
| 2018-05930 | II | 166 | 1 | 7.1 | 31.12 | 1 | 3 |
| 2018-05932 | II | 264 | 1 | 1.69 | 1.81 | 0 | 1 |
| 2018-05936 | II | 195 | 1 | 125.05 | 159.76 | 7 | 8 |
| 2018-06001 | II | 1897 | 0 | 0.15 | 0.37 | 0 | 0 |
| 2018-06003 | II | 568 | 1 | 6.46 | 66.29 | 1 | 4 |
| 2018-06007 | II | 1897 | 0 | 0.06 | 0.93 | 0 | 0 |
| 2018-06009 | II | 667 | 1 | 1.09 | 10.35 | 0 | 1 |
| 2018-06061 | II | 1890 | 0 | 0.3 | 1.52 | 0 | 0 |
| 2018-06142 | II | 1894 | 0 | 0.16 | 0.82 | 0 | 0 |
| 2018-06148 | I | 1891 | 0 | 0.07 | 0.44 | 0 | 0 |
| 2018-06310 | II | 579 | 0 | 0.78 | 4.12 | 0 | 0 |
| 2018-06312 | I | 1889 | 0 | 0.13 | 0.65 | 0 | 0 |
| 2018-06375 | II | 1889 | 1 | 1.59 | 10.1 | 0 | 1 |
| 2018-06497 | I | 1888 | 1 | 4.03 | 15.82 | 0 | 1 |
| 2018-06498 | I | 1888 | 1 | 0.63 | 6.95 | 0 | 1 |
| 2018-06499 | I | 1888 | 0 | 0.12 | 2.67 | 0 | 0 |
| 2018-06564 | II | 1887 | 1 | 2.94 | 6.23 | 0 | 1 |
| 2018-06641 | II | 1887 | 0 | 0.05 | 0.15 | 0 | 0 |
| 2018-06830 | II | 1881 | 1 | 0.36 | 10.72 | 0 | 1 |
| 2018-06846 | I | 1881 | 1 | 0.52 | 13.82 | 0 | 1 |
| 2018-06969 | II | 1877 | 0 | 1.15 | 4.54 | 0 | 0 |
| 2018-07046 | I | 1876 | 0 | 0.23 | 1.01 | 0 | 0 |
| 2018-07145 | II | 1874 | 0 | 0.03 | 0.17 | 0 | 0 |
| 2018-07230 | II | 1873 | 0 | 0.16 | 2.11 | 0 | 0 |
| 2018-07380 | II | 1869 | 1 | 2.34 | 14.7 | 0 | 1 |
| 2018-07474 | II | 1866 | 1 | 0.36 | 6.36 | 0 | 1 |
| 2018-07475 | II | 1866 | 1 | 1.03 | 10.66 | 0 | 1 |
| 2018-08015 | I | 1854 | 0 | 0.06 | 0.57 | 0 | 0 |
| 2018-08160 | II | 1849 | 0 | 0.45 | 0.86 | 0 | 0 |
| 2018-08432 | II | 1847 | 0 | 0.16 | 0.58 | 0 | 0 |
| 2018-08653 | II | 1841 | 0 | 0.79 | 0.97 | 0 | 0 |
