# Supplementary material for: Elucidating the role of N-myristoylation in the excessive membrane localization of PD-L1 in hypoxic cancers and developing a novel NMT1 inhibitor for combination with immune checkpoint blockade therapy
Source: J Exp Clin Cancer Res. 2025 Jul 2;44:181. doi: 10.1186/s13046-025-03438-z (PMC12219335; doi:10.1186/s13046-025-03438-z)
Supplement: Supplementary file 2 — Supplementary Material 2. [file 13046_2025_3438_MOESM2_ESM.docx]

|  | Sequencing results of shRNA interference vector |
| --- | --- |
| H_NMT1-shRNA267(PGMLV-Puro) | GCCAATTTGCAATACGATACAGGCTGTTAGAGAGATAATTAGAATTAATTTGACTGTAAACACAAAGATATTAGTACAAAATACGTGACGTAGAAAGTAATAATTTCTTGGGTAGTTTGCAGTTTTAAAATTATGTTTTAAAATGGACTATCATATGCTTACCGTAACTTGAAAGTATTTCGATTTCTTGGCTTTATATATCTTGTGGAAAGGACGAGGATCCGCCAGGAAATACAGAAGGCCATCTCGAGATGGCCTTCTGTATTTCCTGGTTTTTTAATTCTAGTTATTAATAGTAATCAATTACGGGGTCATTAGTTCATAGCCCATATATGGAGTTCCGCGTTACATAACTTACGGTAAATGGCCCGCCTGGCTGACCGCCCAACGACCCCCGCCCATTGACGTCAATAATGACGTATGTTCCCATAGTAACGCCAATAGGGACTTTCCATTGACGTCAATGGGTGGAGTATTTACGGTAAACTGCCCACTTGGCAGTACATCAAGTGTATCATATGCCAAGTACGCCCCCTATTGACGTCAATGACGGTAAATGGCCCGCCTGGCATTATGCCCAGTACATGACCTTATGGGACTTTCCTACTTGGCAGTACATCTACGTATTAGTCATCGCTATTACCATGGTGATGCGGTTTTGGCAGTACATCAATGGGCGTGGATAGCGGTTTGACTCACGGGGATTTCCAAGTCTCCACCCCATTGACGTCAATGGGAGTTTGTTTTGGCACCAAAATCAACGGGACTTTCCAAAATGTCGTAACAACTCCGCCCCATTGACGCAAATGGGCGGTAGGCGTGTACGGTGGGAGGTCTATATAAGCAGAGCTGGTTTAGTGAACCGTCAGATCCGCTAGCGCTACCGGTCGCCACCATGACCGAGTACAAGCCCACGGTGCGCCTCGCCACCCGCGACGACGTCCCCAGGGCCGTACGCACCCTCGCCGCCGCGTTCGCCGACTACCCCGCCACGCGCACACCGTCGATCCGGGACCGCCACATCGAGCGGGTCACCGAGCTGCAAGAACTCTTCCTCACGCGCGTCGGGCTCGACATCGCAGGTGTGGGTCGCGGACGACGGCGCCGCGTTGCGTCTGGACCACGCCGGAGAAAGGCGTTCGAAGCG |
| H_NMT1-shRNA557(PGMLV-Puro) | CATTTGCAATACGATACAAGGCTGTTAGAGAGATAATTAGAATTAATTTGACTGTAAACACAAAGATATTAGTACAAAATACGTGACGTAGAAAGTAATAATTTCTTGGGTAGTTTGCAGTTTTAAAATTATGTTTTAAAATGGACTATCATATGCTTACCGTAACTTGAAAGTATTTCGATTTCTTGGCTTTATATATCTTGTGGAAAGGACGAGGATCCACATGTTCCGATTTGATTATTCTCGAGAATAATCAAATCGGAACATGTTTTTTTAATTCTAGTTATTAATAGTAATCAATTACGGGGTCATTAGTTCATAGCCCATATATGGAGTTCCGCGTTACATAACTTACGGTAAATGGCCCGCCTGGCTGACCGCCCAACGACCCCCGCCCATTGACGTCAATAATGACGTATGTTCCCATAGTAACGCCAATAGGGACTTTCCATTGACGTCAATGGGTGGAGTATTTACGGTAAACTGCCCACTTGGCAGTACATCAAGTGTATCATATGCCAAGTACGCCCCCTATTGACGTCAATGACGGTAAATGGCCCGCCTGGCATTATGCCCAGTACATGACCTTATGGGACTTTCCTACTTGGCAGTACATCTACGTATTAGTCATCGCTATTACCATGGTGATGCGGTTTTGGCAGTACATCAATGGGCGTGGATAGCGGTTTGACTCACGGGGATTTCCAAGTCTCCACCCCATTGACGTCAATGGGAGTTTGTTTTGGCACCAAAATCAACGGGACTTTCCAAAATGTCGTAACAACTCCGCCCCATTGACGCAAATGGGCGGTAGGCGTGTACGGTGGGAGGTCTATATAAGCAGAGCTGGTTTAGTGAACCGTCAGATCCGCTAGCGCTACCGGTCGCCACCATGACCGAGTACAAGCCCACGGTGCGCCTCGCCACCCGCGACGACGTCCCCAGGGCCGTACGCACCCTCGCCGCCGCGTTCGCCGACTACCCCGCCACGCGCCACACCGTCGATCCGGACCGCCACATCGAGCGGGTCACCGAGCTGCAGAACTCTTCCTCACGCCGCGTCGGGCTCGACATCGGCAAGGTGGTGGGTCGCGGACGACGGCGCCCGCGTGCGTCTGGACCACCGCCCGAAAGCGTCGTCGGGGCGGTGTTCGCCCGAGAATCGTCCCGGCCATGCGGTTGAAGCGATCCCGCTTGCCCGCCACACCAGAATGGGGAAGGCCCTCTCTTGGGCGGC |
| H_NMT1-shRNA658(PGMLV-Puro) | CATTGCATCGATACAGGCTGTTAGAGAGATAATTAGAATTAATTTGACTGTAAACACAAAGATATTAGTACAAAATACGTGACGTAGAAAGTAATAATTTCTTGGGTAGTTTGCAGTTTTAAAATTATGTTTTAAAATGGACTATCATATGCTTACCGTAACTTGAAAGTATTTCGATTTCTTGGCTTTATATATCTTGTGGAAAGGACGAGGATCCGCGGAAATTGGTTGGGTTCATTCTCGAGAATGAACCCAACCAATTTCCGTTTTTTAATTCTAGTTATTAATAGTAATCAATTACGGGGTCATTAGTTCATAGCCCATATATGGAGTTCCGCGTTACATAACTTACGGTAAATGGCCCGCCTGGCTGACCGCCCAACGACCCCCGCCCATTGACGTCAATAATGACGTATGTTCCCATAGTAACGCCAATAGGGACTTTCCATTGACGTCAATGGGTGGAGTATTTACGGTAAACTGCCCACTTGGCAGTACATCAAGTGTATCATATGCCAAGTACGCCCCCTATTGACGTCAATGACGGTAAATGGCCCGCCTGGCATTATGCCCAGTACATGACCTTATGGGACTTTCCTACTTGGCAGTACATCTACGTATTAGTCATCGCTATTACCATGGTGATGCGGTTTTGGCAGTACATCAATGGGCGTGGATAGCGGTTTGACTCACGGGGATTTCCAAGTCTCCACCCCATTGACGTCAATGGGAGTTTGTTTTGGCACCAAAATCAACGGGACTTTCCAAAATGTCGTAACAACTCCGCCCCATTGACGCAAATGGGCGGTAGGCGTGTACGGTGGGAGGTCTATATAAGCAGAGCTGGTTTAGTGAACCGTCAGATCCGCTAGCGCTACCGGTCGCCACCATGACCGAGTACAAGCCCACGGTGCGCCTCGCCACCCGCGACGACGTCCCCAGGGCCGTACGCACCCTCGCCGCCGCGTTCGCCGACTACCCCGCCACGCGCCACACCGTCGATCCGGACCGCCACATCGAGCGGGTCACCGAGCTGCGAG |
| GMLV-CMV-H_NMT1(shMT)-3×Flag-PGK-Blasticidin | GCCACCATGGCGGACGAGAGTGAGACAGCAGTGAAGCCGCCGGCACCTCCGCTGCCGCAGATGATGGAAGGGAACGGGAACGGCCATGAGCACTGCAGCGATTGCGAGAATGAGGAGGACAACAGCTACAACCGGGGTGGTTTGAGTCCAGCCAATGACACTGGAGCCAAAAAGAAGAAAAAGAAACAAAAAAAGAAGAAAGAAAAAGGCAGTGAGACAGATTCAGCCCAGGATCAGCCTGTGAAGATGAACTCTTTGCCAGCAGAGAGGATCCAGGAAATACAGAAGGCCATTGAGCTGTTCTCAGTGGGTCAGGGACCTGCCAAAACCATGGAGGAGGCTAGCAAGCGAAGCTACCAGTTCTGGGATACGCAGCCCGTCCCCAAGCTGGGCGAAGTGGTGAACACCCATGGCCCCGTGGAGCCTGACAAGGACAATATCCGCCAGGAGCCCTACACCCTGCCCCAGGGCTTCACCTGGGATGCTTTGGACCTGGGCGATCGTGGTGTGCTAAAAGAACTGTACACCCTCCTGAATGAGAACTATGTGGAAGATGATGACAATATGTTTCGCTTCGACTACTCCCCGGAGTTTCTTTTGTGGGCTCTCCGGCCACCCGGCTGGCTCCCCCAGTGGCACTGTGGGGTTCGAGTGGTCTCAAGTCGGAAATTGGTTGGGTTCATTAGCGCCATCCCAGCAAACATCCATATCTATGACACAGAGAAGAAGATGGTAGAGATCAACTTCCTGTGTGTCCACAAGAAGCTGCGTTCCAAGAGGGTTGCTCCAGTTCTGATCCGAGAGATCACCAGGCGGGTTCACCTGGAGGGCATCTTCCAAGCAGTTTACACTGCCGGGGTGGTACTACCAAAGCCCGTTGGCACCTGCAGGTATTGGCATCGGTCCCTAAACCCACGGAAGCTGATTGAAGTGAAGTTCTCCCACCTGAGCAGAAATATGACCATGCAGCGCACCATGAAGCTCTACCGACTGCCAGAGACTCCCAAGACAGCTGGGCTGCGACCAATGGAAACAAAGGACATTCCAGTAGTGCACCAGCTCCTCACCAGGTACTTGAAGCAATTTCACCTTACGCCCGTCATGAGCCAGGAGGAGGTGGAGCACTGGTTCTACCCCCAGGAGAATATCATCGACACTTTCGTGGTGGAGAACGCAAACGGAGAGGTGACAGATTTCCTGAGCTTTTATACGCTGCCCTCCACCATCATGAACCATCCAACCCACAAGAGTCTCAAAGCTGCTTATTCTTTCTACAACGTTCACACCCAGACCCCTCTTCTAGACCTCATGAGCGACGCCCTTGTCCTCGCCAAAATGAAAGGGTTTGATGTGTTCAATGCACTGGATCTCATGGAGAACAAAACCTTCCTGGAGAAGCTCAAGTTTGGCATAGGGGACGGCAACCTGCAGTATTACCTTTACAATTGGAAATGCCCCAGCATGGGGGCAGAGAAGGTTGGACTGGTGCTACAA |
|  | Sequencing results of PD-L1 fluorescently labeled plasmid, CHP1 site mutation and control plasmid, and fluorescent plasmid |
| PGMLV-CMV-H_CD274(PDL1)-eGFP-PGK-Puro | GCCACCATGAGGATATTTGCTGTCTTTATATTCATGACCTACTGGCATTTGCTGAACGCATTTACTGTCACGGTTCCCAAGGACCTATATGTGGTAGAGTATGGTAGCAATATGACAATTGAATGCAAATTCCCAGTAGAAAAACAATTAGACCTGGCTGCACTAATTGTCTATTGGGAAATGGAGGATAAGAACATTATTCAATTTGTGCATGGAGAGGAAGACCTGAAGGTTCAGCATAGTAGCTACAGACAGAGGGCCCGGCTGTTGAAGGACCAGCTCTCCCTGGGAAATGCTGCACTTCAGATCACAGATGTGAAATTGCAGGATGCAGGGGTGTACCGCTGCATGATCAGCTATGGTGGTGCCGACTACAAGCGAATTACTGTGAAAGTCAATGCCCCATACAACAAAATCAACCAAAGAATTTTGGTTGTGGATCCAGTCACCTCTGAACATGAACTGACATGTCAGGCTGAGGGCTACCCCAAGGCCGAAGTCATCTGGACAAGCAGTGACCATCAAGTCCTGAGTGGTAAGACCACCACCACCAATTCCAAGAGAGAGGAGAAGCTTTTCAATGTGACCAGCACACTGAGAATCAACACAACAACTAATGAGATTTTCTACTGCACTTTTAGGAGATTAGATCCTGAGGAAAACCATACAGCTGAATTGGTCATCCCAGAACTACCTCTGGCACATCCTCCAAATGAAAGGACTCACTTGGTAATTCTGGGAGCCATCTTATTATGCCTTGGTGTAGCACTGACATTCATCTTCCGTTTAAGAAAAGGGAGAATGATGGATGTGAAAAAATGTGGCATCCAAGATACAAACTCAAAGAAGCAAAGTGATACACATTTGGAGGAGACG |
| PGMLV-CMV-H_CHP1-3×Flag-PGK-Puro | GCCACCATGGGTTCTCGGGCCTCCACGTTACTGCGGGACGAAGAGCTCGAGGAGATCAAGAAGGAGACCGGCTTTTCCCACAGTCAAATCACTCGCCTCTACAGCCGGTTCACCAGCCTGGACAAAGGAGAGAATGGGACTCTCAGCCGGGAAGATTTCCAGAGGATTCCAGAACTTGCCATCAACCCACTGGGGGACCGGATCATCAATGCCTTCTTTCCAGAGGGAGAGGACCAGGTAAACTTCCGTGGATTCATGCGAACTTTGGCTCATTTCCGCCCCATTGAGGATAATGAAAAGAGCAAAGATGTGAATGGACCCGAACCACTCAACAGCCGAAGCAACAAACTGCACTTTGCTTTTCGACTATATGATTTGGATAAAGATGAAAAGATCTCCCGTGATGAGCTGTTACAGGTGCTACGCATGATGGTCGGAGTAAATATCTCAGATGAGCAGCTGGGCAGCATCGCAGACAGGACCATTCAGGAGGCTGATCAGGATGGGGACAGTGCCATATCTTTCACAGAATTTGTTAAGGTTTTGGAGAAGGTGGATGTAGAACAGAAAATGAGCATCCGATTTCTTCAC |
| PGMLV-CMV-H_CHP1-mCherry-PGK-Puro | GCCACCATGGGTTCTCGGGCCTCCACGTTACTGCGGGACGAAGAGCTCGAGGAGATCAAGAAGGAGACCGGCTTTTCCCACAGTCAAATCACTCGCCTCTACAGCCGGTTCACCAGCCTGGACAAAGGAGAGAATGGGACTCTCAGCCGGGAAGATTTCCAGAGGATTCCAGAACTTGCCATCAACCCACTGGGGGACCGGATCATCAATGCCTTCTTTCCAGAGGGAGAGGACCAGGTAAACTTCCGTGGATTCATGCGAACTTTGGCTCATTTCCGCCCCATTGAGGATAATGAAAAGAGCAAAGATGTGAATGGACCCGAACCACTCAACAGCCGAAGCAACAAACTGCACTTTGCTTTTCGACTATATGATTTGGATAAAGATGAAAAGATCTCCCGTGATGAGCTGTTACAGGTGCTACGCATGATGGTCGGAGTAAATATCTCAGATGAGCAGCTGGGCAGCATCGCAGACAGGACCATTCAGGAGGCTGATCAGGATGGGGACAGTGCCATATCTTTCACAGAATTTGTTAAGGTTTTGGAGAAGGTGGATGTAGAACAGAAAATGAGCATCCGATTTCTTCAC |
| PGMLV-CMV-H_CHP1 MT-mCherry-PGK-Puro | GCCACCATGGCAGCGGCAGCAGCGACGTTACTGCGGGACGAAGAGCTCGAGGAGATCAAGAAGGAGACCGGCTTTTCCCACAGTCAAATCACTCGCCTCTACAGCCGGTTCACCAGCCTGGACAAAGGAGAGAATGGGACTCTCAGCCGGGAAGATTTCCAGAGGATTCCAGAACTTGCCATCAACCCACTGGGGGACCGGATCATCAATGCCTTCTTTCCAGAGGGAGAGGACCAGGTAAACTTCCGTGGATTCATGCGAACTTTGGCTCATTTCCGCCCCATTGAGGATAATGAAAAGAGCAAAGATGTGAATGGACCCGAACCACTCAACAGCCGAAGCAACAAACTGCACTTTGCTTTTCGACTATATGATTTGGATAAAGATGAAAAGATCTCCCGTGATGAGCTGTTACAGGTGCTACGCATGATGGTCGGAGTAAATATCTCAGATGAGCAGCTGGGCAGCATCGCAGACAGGACCATTCAGGAGGCTGATCAGGATGGGGACAGTGCCATATCTTTCACAGAATTTGTTAAGGTTTTGGAGAAGGTGGATGTAGAACAGAAAATGAGCATCCGATTTCTTCAC |
| PGMLV-CMV-H_CHP1 MT-3×Flag-PGK-Puro | GCCACCATGGCAGCGGCAGCAGCGACGTTACTGCGGGACGAAGAGCTCGAGGAGATCAAGAAGGAGACCGGCTTTTCCCACAGTCAAATCACTCGCCTCTACAGCCGGTTCACCAGCCTGGACAAAGGAGAGAATGGGACTCTCAGCCGGGAAGATTTCCAGAGGATTCCAGAACTTGCCATCAACCCACTGGGGGACCGGATCATCAATGCCTTCTTTCCAGAGGGAGAGGACCAGGTAAACTTCCGTGGATTCATGCGAACTTTGGCTCATTTCCGCCCCATTGAGGATAATGAAAAGAGCAAAGATGTGAATGGACCCGAACCACTCAACAGCCGAAGCAACAAACTGCACTTTGCTTTTCGACTATATGATTTGGATAAAGATGAAAAGATCTCCCGTGATGAGCTGTTACAGGTGCTACGCATGATGGTCGGAGTAAATATCTCAGATGAGCAGCTGGGCAGCATCGCAGACAGGACCATTCAGGAGGCTGATCAGGATGGGGACAGTGCCATATCTTTCACAGAATTTGTTAAGGTTTTGGAGAAGGTGGATGTAGAACAGAAAATGAGCATCCGATTTCTTCAC |

|  | 5’ to 3’ |
| --- | --- |
| NMT1 promoter-sense-902 | GCCTTACTACAGAGTCTGGCACTT |
| NMT1 promoter-antisense-1032 | GGTCTCGAACTCCTGGCCTCTA |
