# Supplementary figures and images for: Elucidating the role of N-myristoylation in the excessive membrane localization of PD-L1 in hypoxic cancers and developing a novel NMT1 inhibitor for combination with immune checkpoint blockade therapy
Source: J Exp Clin Cancer Res. 2025 Jul 2;44:181. doi: 10.1186/s13046-025-03438-z (PMC12219335; doi:10.1186/s13046-025-03438-z)

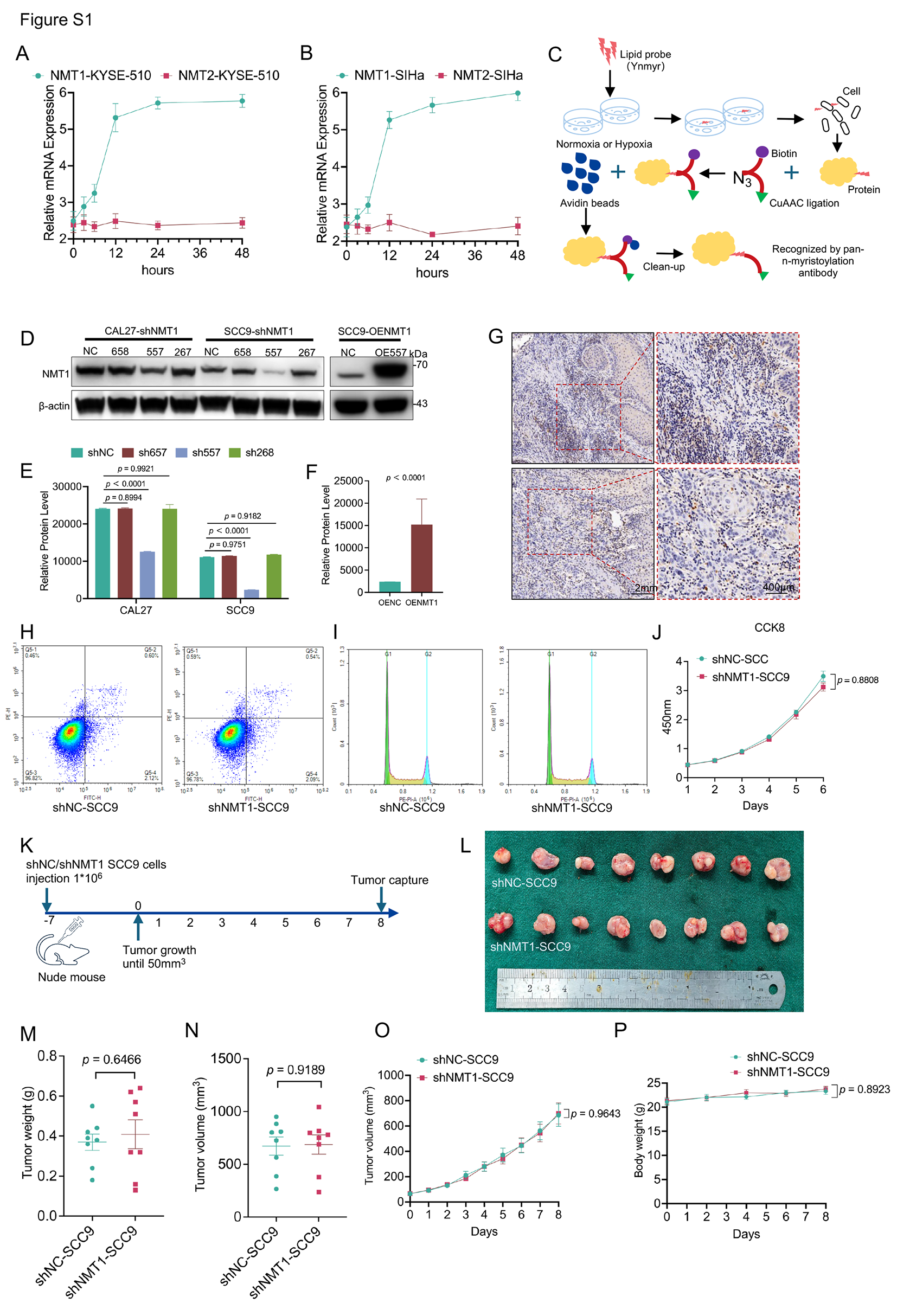

Supplement: Supplementary file 6 — Supplementary Material 6: Figure S1. A. Expression of NMT1 and NMT2 at the mRNA level in the KYSE-510 cell line under hypoxic conditions. B. Expression of NMT1 and NMT2 at the mRNA level in the SiHa cell line under hypoxic conditions. C. Schematic diagram of the functional assessment using an alkyne-tagged myristic acid (YnMyr) probe. D. Verification of NMT1 knockdown in SCC9 and CAL27 cell lines via lentiviral transfection and verification of synonymous mutation overexpression of NMT1 in the SCC9 cell line via lentiviral transfection. E. Quantitative analysis of NMT1 knockdown levels in SCC9 and CAL27 cell lines. F. Quantitative analysis of NMT1 overexpression in the SCC9 cell line. G. Example of high NMT1 expression detected via immunohistochemical staining of tumor tissue slices with an IRS ≥ 1 (scale bars: 2 mm and 400 μm). H: Detection of apoptosis in shNMT1-SCC9 and shNC-SCC9 cells. I: Cell cycle analysis of shNMT1-SCC9 and shNC-SCC9 cells. J. CCK8 assay was used to evaluate the proliferation capacity of shNMT1-SCC9 and shNC-SCC9 cells (P value = 0.8808). K. Subcutaneous injection of lentivirus-transfected shNMT1-SCC9 cells and control lentivirus-transfected SCC9 cells into the backs of nude mice. Observations began after tumor formation, and the mice were sacrificed 15 days after injection, which was 8 days after tumor formation. L. Tumor images showing the tumorigenic effect. M. Final tumor weight. N. Final tumor volume. O. Tumor growth volume. O. Tumor growth volume. [file 13046_2025_3438_MOESM6_ESM.tif]

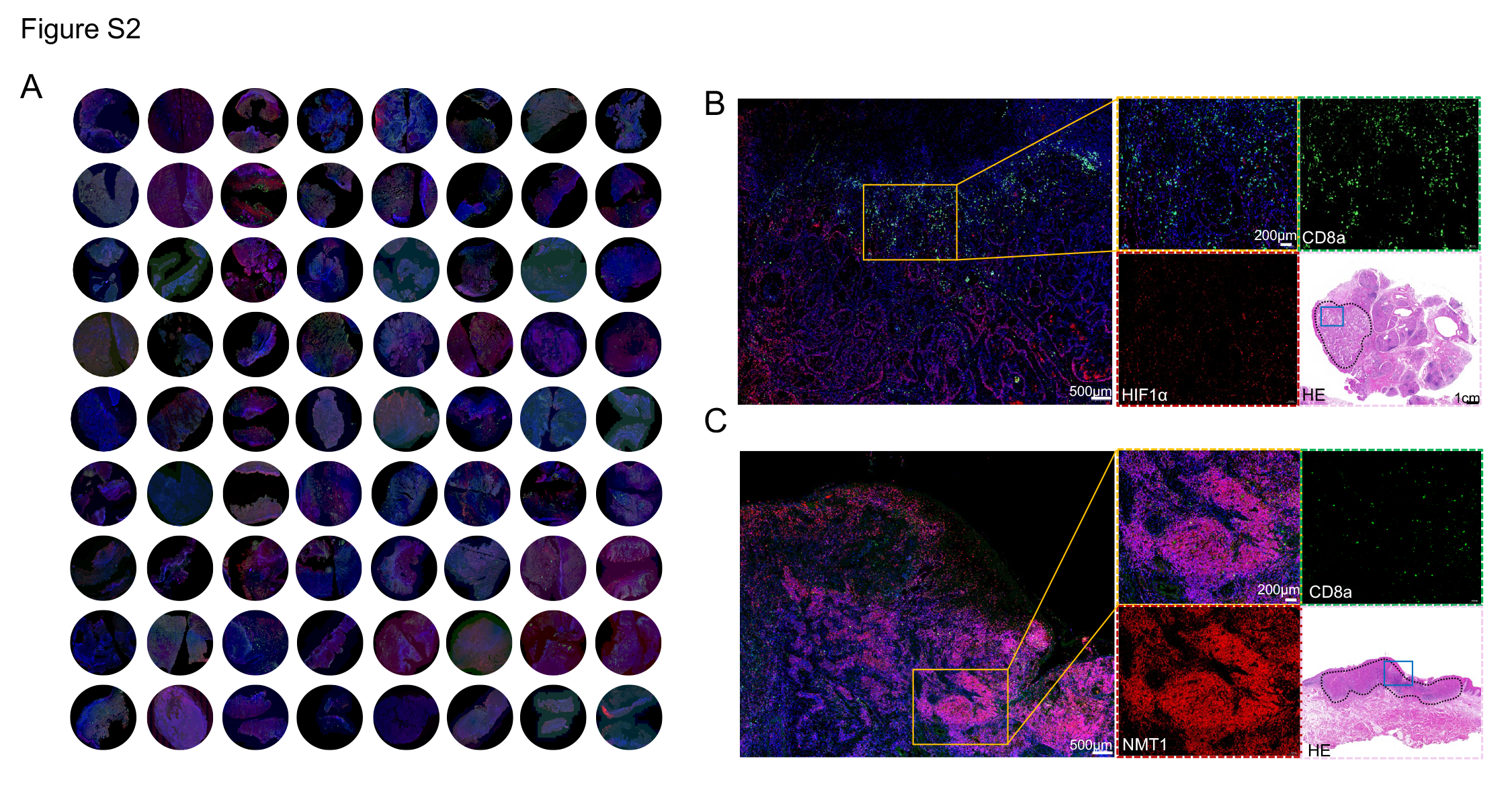

Supplement: Supplementary file 7 — Supplementary Material 7: Figure S2. A. Thumbnail images of immunohistochemical staining of 72 OSCC tumor tissues. B. Schematic representation of the relationship between HIF1α and CD8a expression via immunohistochemical staining of 72 OSCC tumor tissues (scale bars: 500 μm, 200 μm, and 1 cm). C. Schematic representation of the relationship between NMT1 and CD8a expression via immunohistochemical staining of 72 OSCC tumor tissues (scale bars: 500 μm, 200 μm, and 1 cm). [file 13046_2025_3438_MOESM7_ESM.tif]

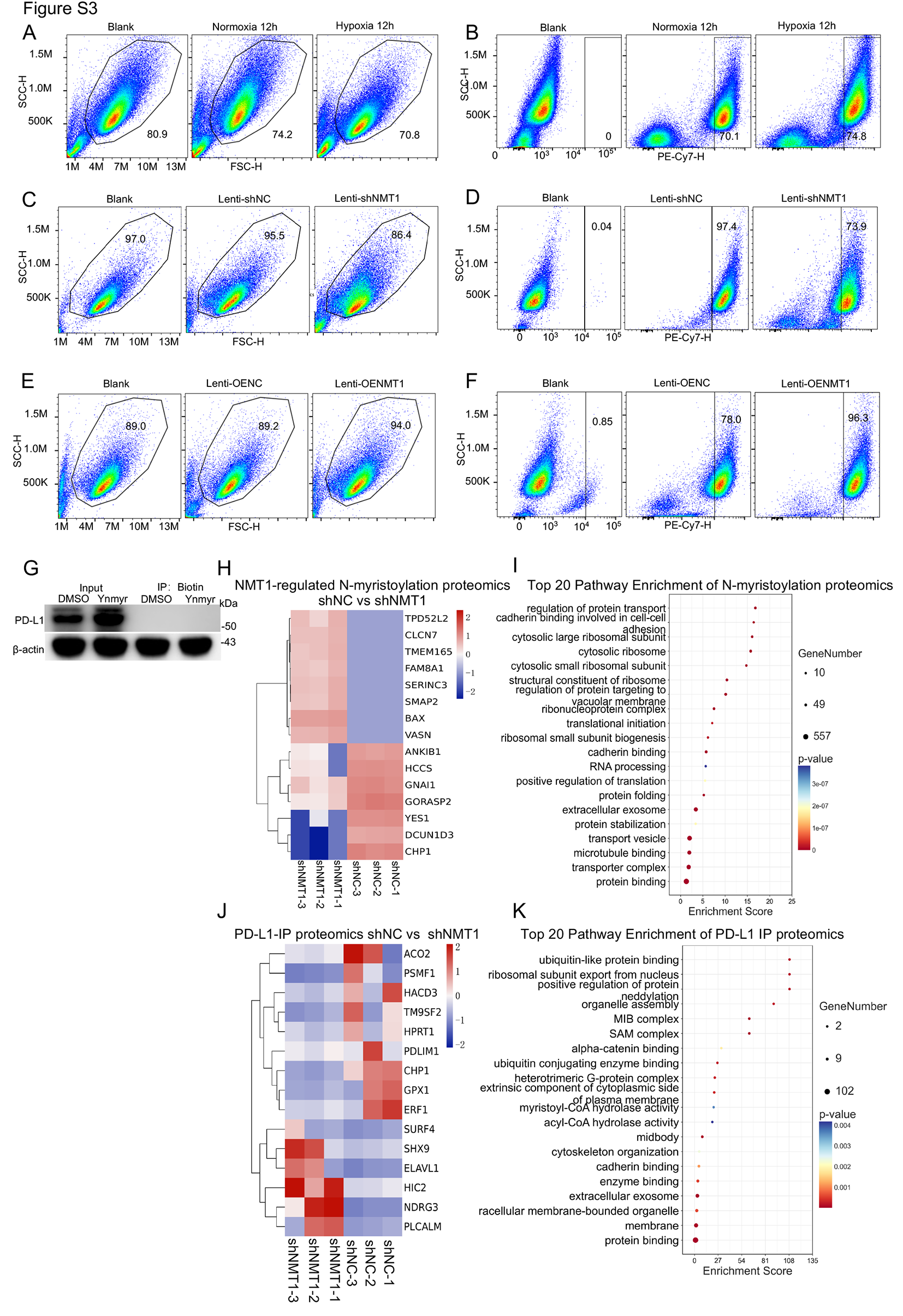

Supplement: Supplementary file 8 — Supplementary Material 8: Figure S3. A. Cell population with PD-L1 cell membrane staining under hypoxic and normoxic conditions; cell line: SCC9. B. Expression intensity of PD-L1 on the cell membrane under hypoxic and normoxic conditions; cell line: SCC9. C. Cell population with PD-L1 cell membrane staining after NMT1 knockdown; cell line: SCC9. D. Expression intensity of PD-L1 on the cell membrane after NMT1 knockdown; cell line: SCC9. E. Cell population with PD-L1 cell membrane staining after NMT1 overexpression; cell line: SCC9. F. Expression intensity of PD-L1 on the cell membrane after NMT1 overexpression; cell line: SCC9. G. Subsequent detection with an anti-PD-L1 antibody on proteins transferred to a PVDF membrane from the myristic acid probe labeling experiment did not reveal PD-L1 among the N-myristoylated proteins, indicating that PD-L1 is not directly modified by myristoylation. H. Heatmap of differentially expressed proteins identified via NMT1-related myristoylation proteomics. I. Pathway enrichment map of differentially expressed proteins identified via NMT1-related myristoylation proteomics. J. Heatmap of differentially expressed proteins identified via PD-L1 binding-related proteomics. H. Fitting results of the two-omics data. K. Pathway enrichment map of differentially expressed proteins identified via PD-L1 binding-related proteomics. [file 13046_2025_3438_MOESM8_ESM.tif]

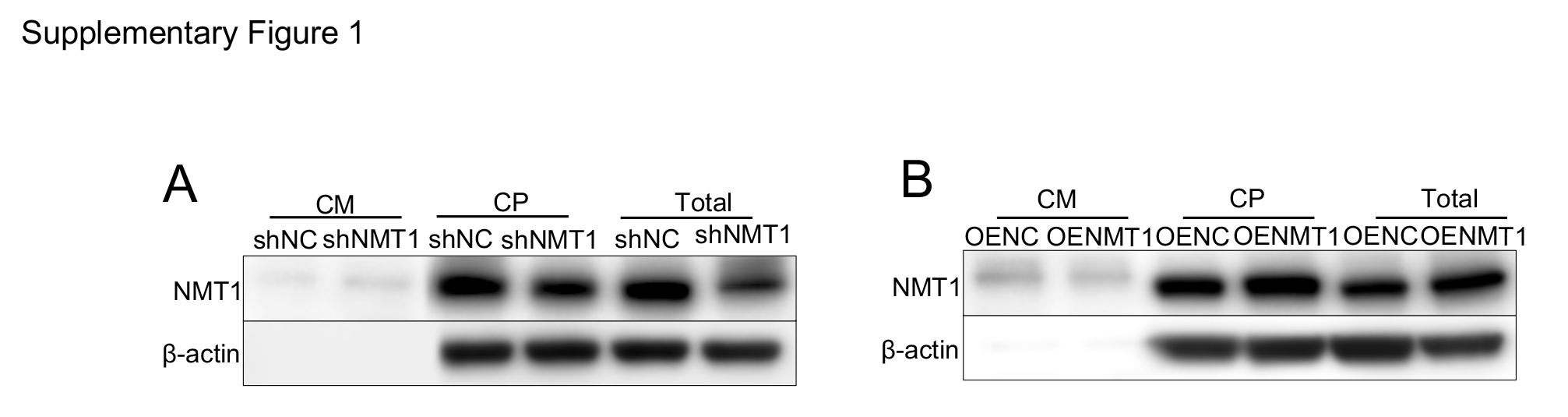

Supplement: Supplementary file 9 — Supplementary Material 9. [file 13046_2025_3438_MOESM9_ESM.tif]

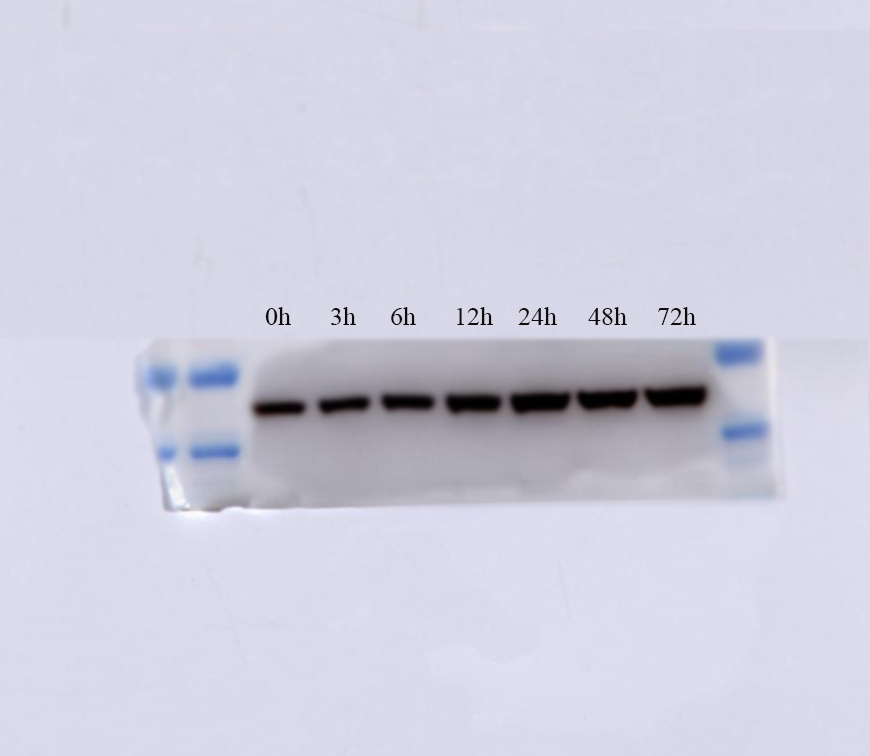

Supplement: Supplementary file 13 — Supplementary Material 13. [file 13046_2025_3438_MOESM13_ESM.zip › full uncropped Gels and Blots image/Fig 1E HIF1a-blots(1-6gel).tif]

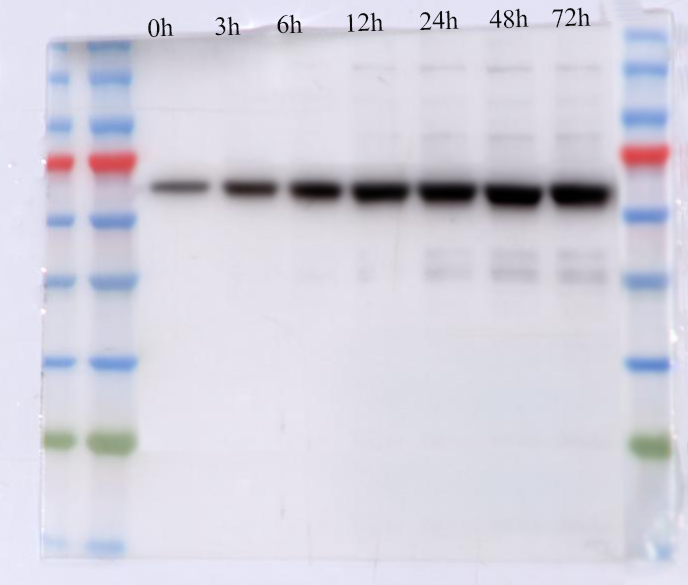

Supplement: Supplementary file 13 — Supplementary Material 13. [file 13046_2025_3438_MOESM13_ESM.zip › full uncropped Gels and Blots image/Fig1ENMT1-blots(1-6gel).tif]

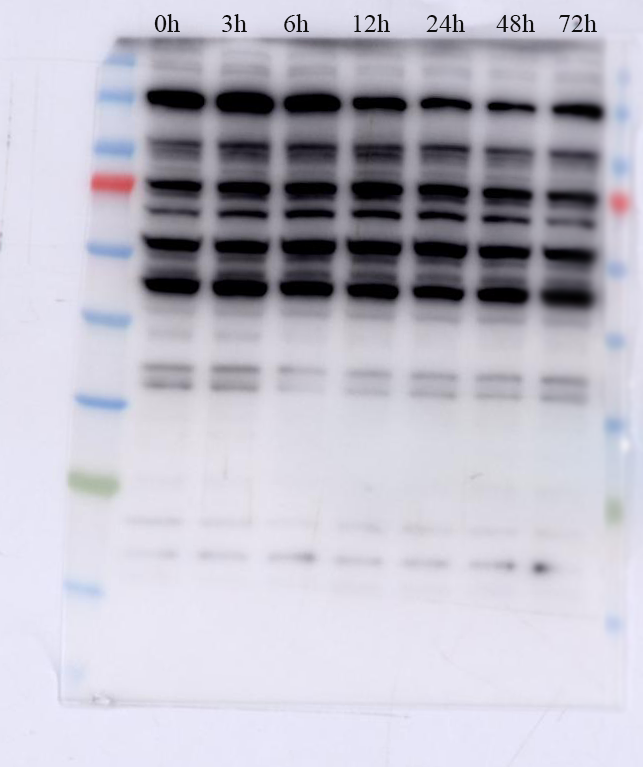

Supplement: Supplementary file 13 — Supplementary Material 13. [file 13046_2025_3438_MOESM13_ESM.zip › full uncropped Gels and Blots image/Fig1ENMT2-blots(1-6gel).tif]

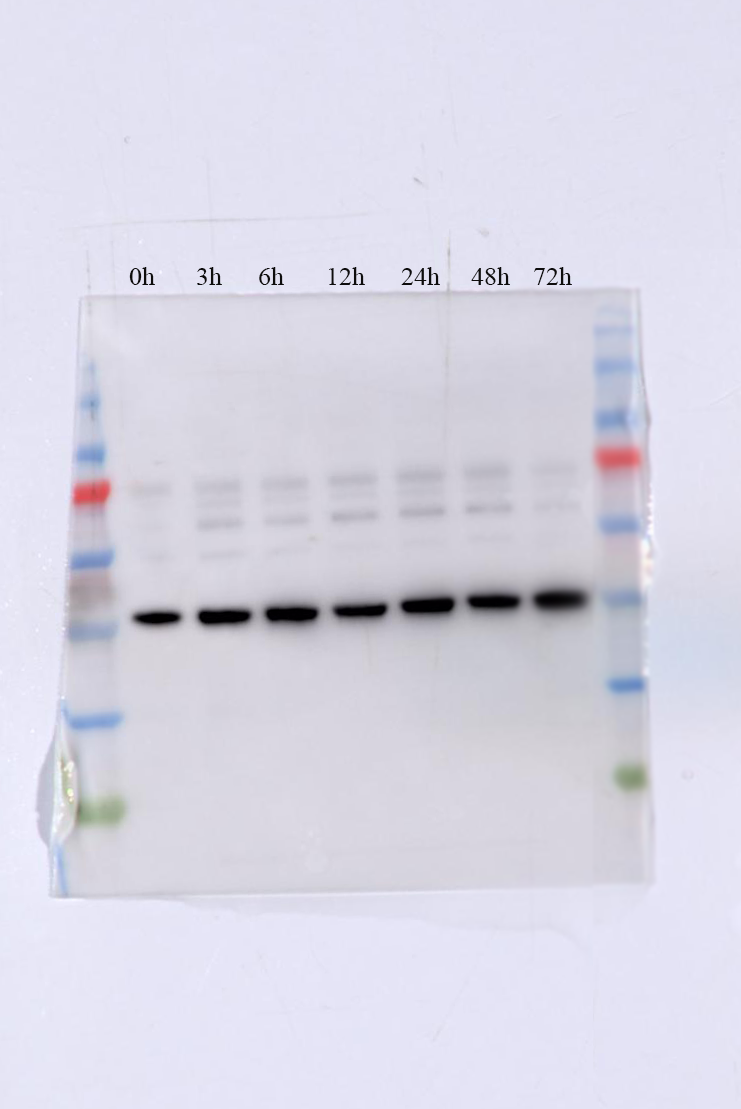

Supplement: Supplementary file 13 — Supplementary Material 13. [file 13046_2025_3438_MOESM13_ESM.zip › full uncropped Gels and Blots image/Fig1Eactin-blots(1-6gel).tif]

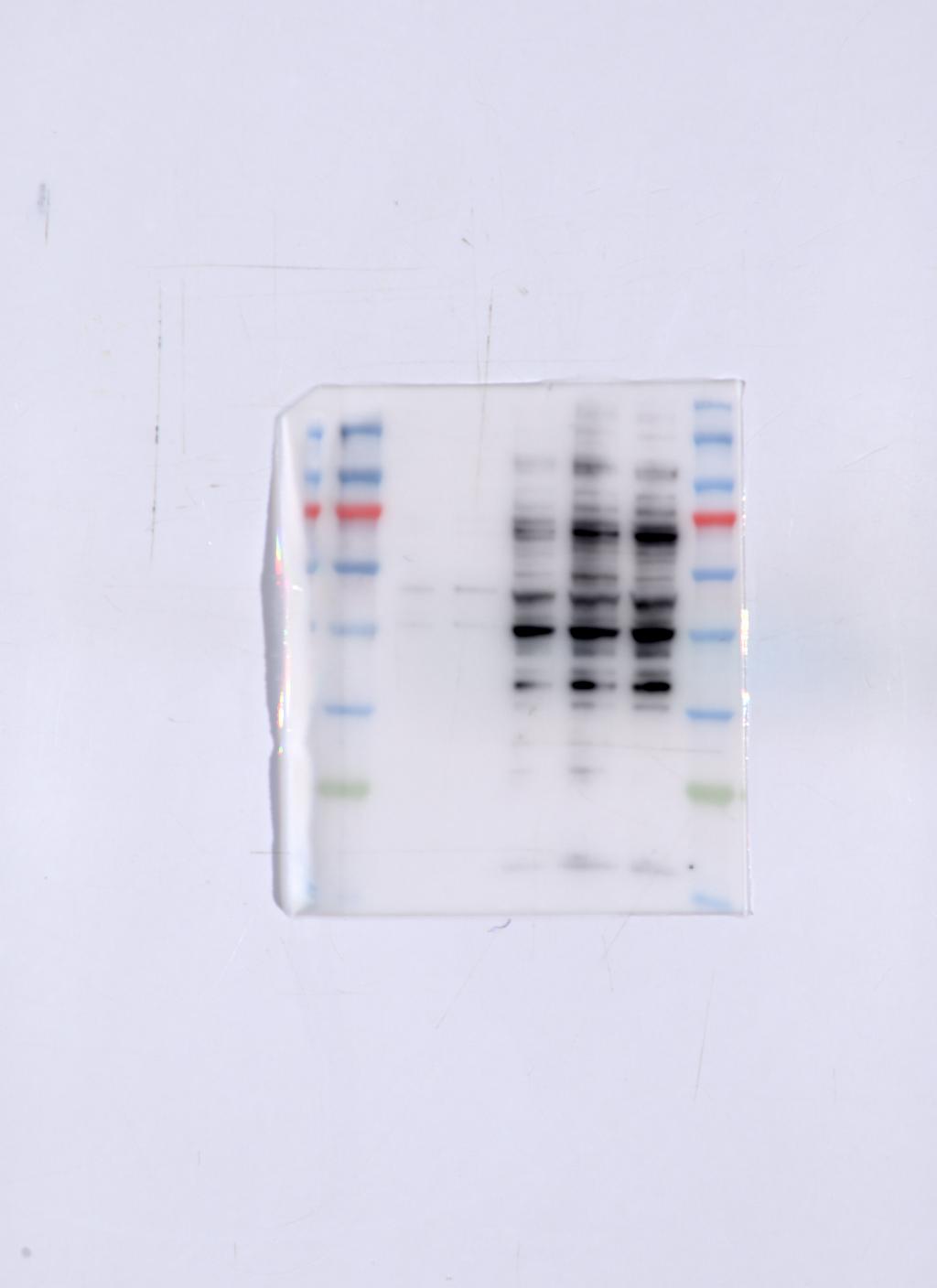

Supplement: Supplementary file 13 — Supplementary Material 13. [file 13046_2025_3438_MOESM13_ESM.zip › full uncropped Gels and Blots image/Fig1H-Pan-Nmyr-blot.jpg]

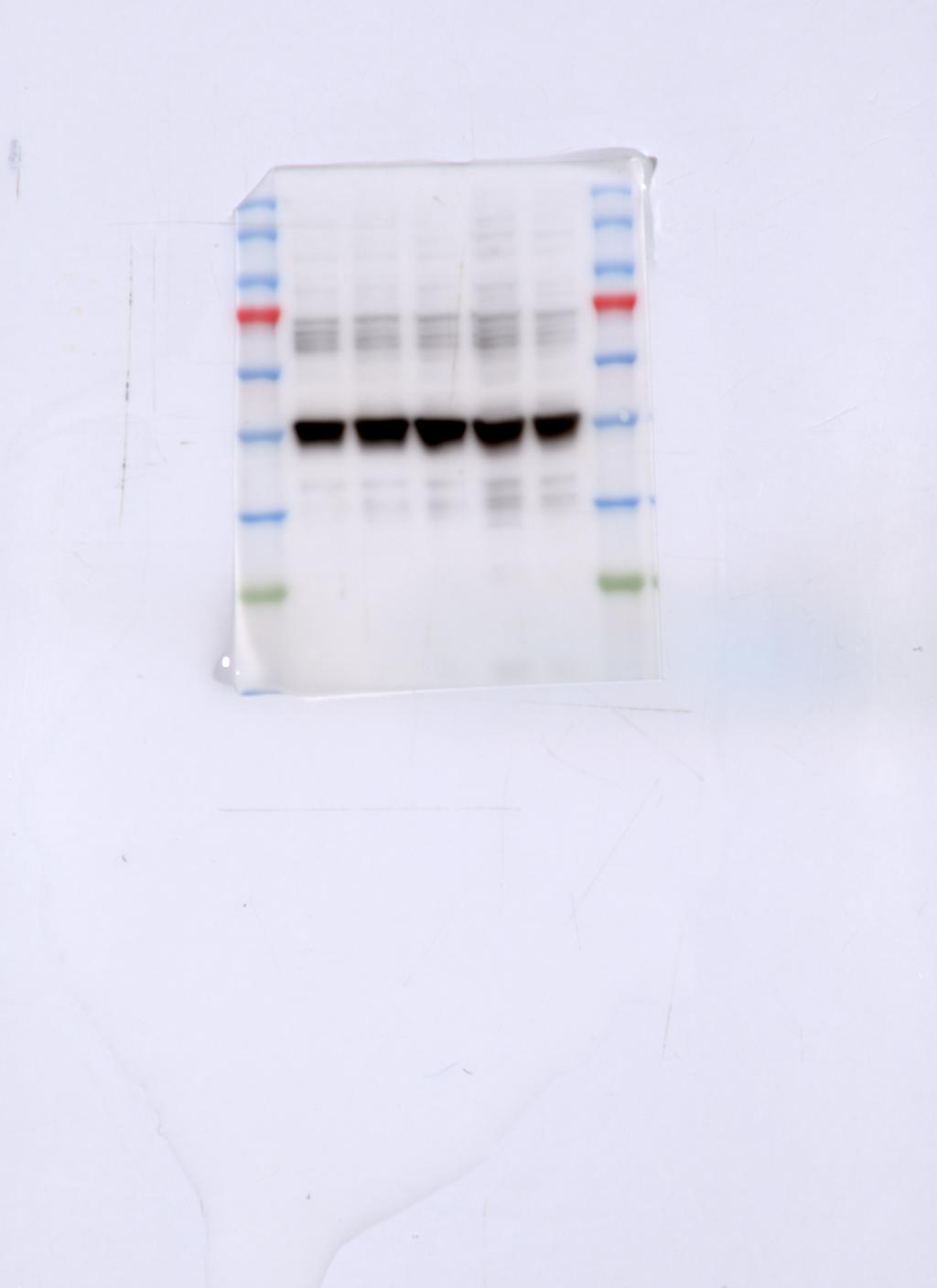

Supplement: Supplementary file 13 — Supplementary Material 13. [file 13046_2025_3438_MOESM13_ESM.zip › full uncropped Gels and Blots image/Fig1H-actin-blot.jpg]

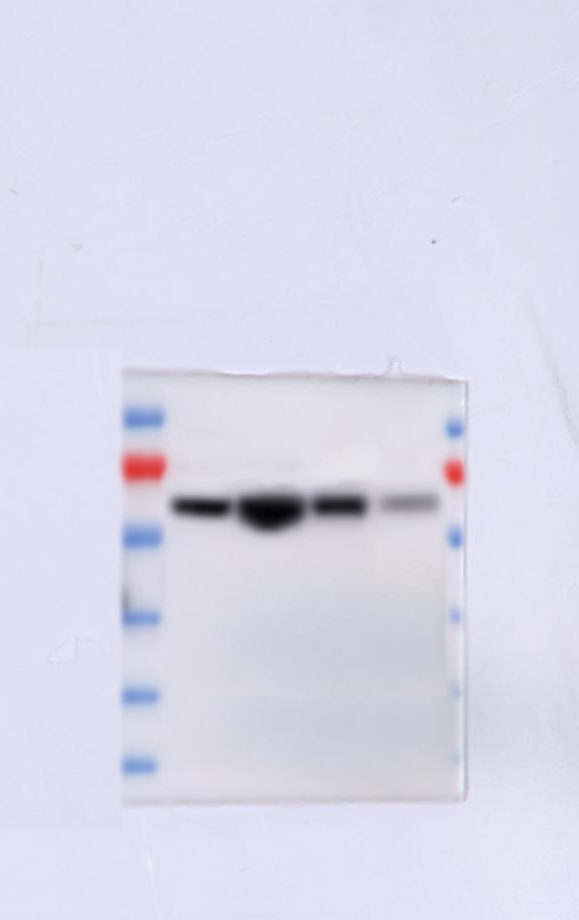

Supplement: Supplementary file 13 — Supplementary Material 13. [file 13046_2025_3438_MOESM13_ESM.zip › full uncropped Gels and Blots image/Fig1J-NMT1-blot.tif]

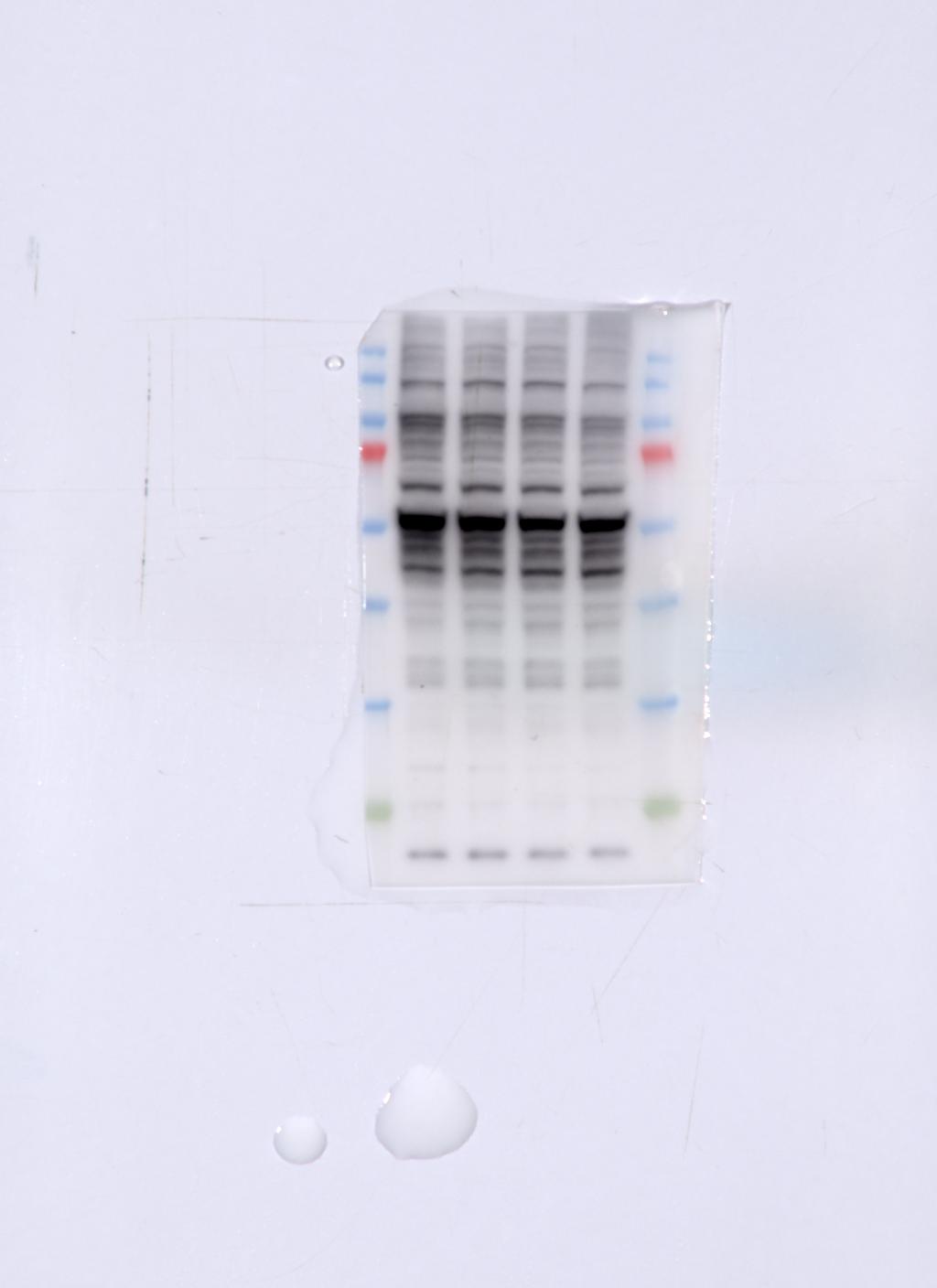

Supplement: Supplementary file 13 — Supplementary Material 13. [file 13046_2025_3438_MOESM13_ESM.zip › full uncropped Gels and Blots image/Fig1J-NMT2-blots.jpg]

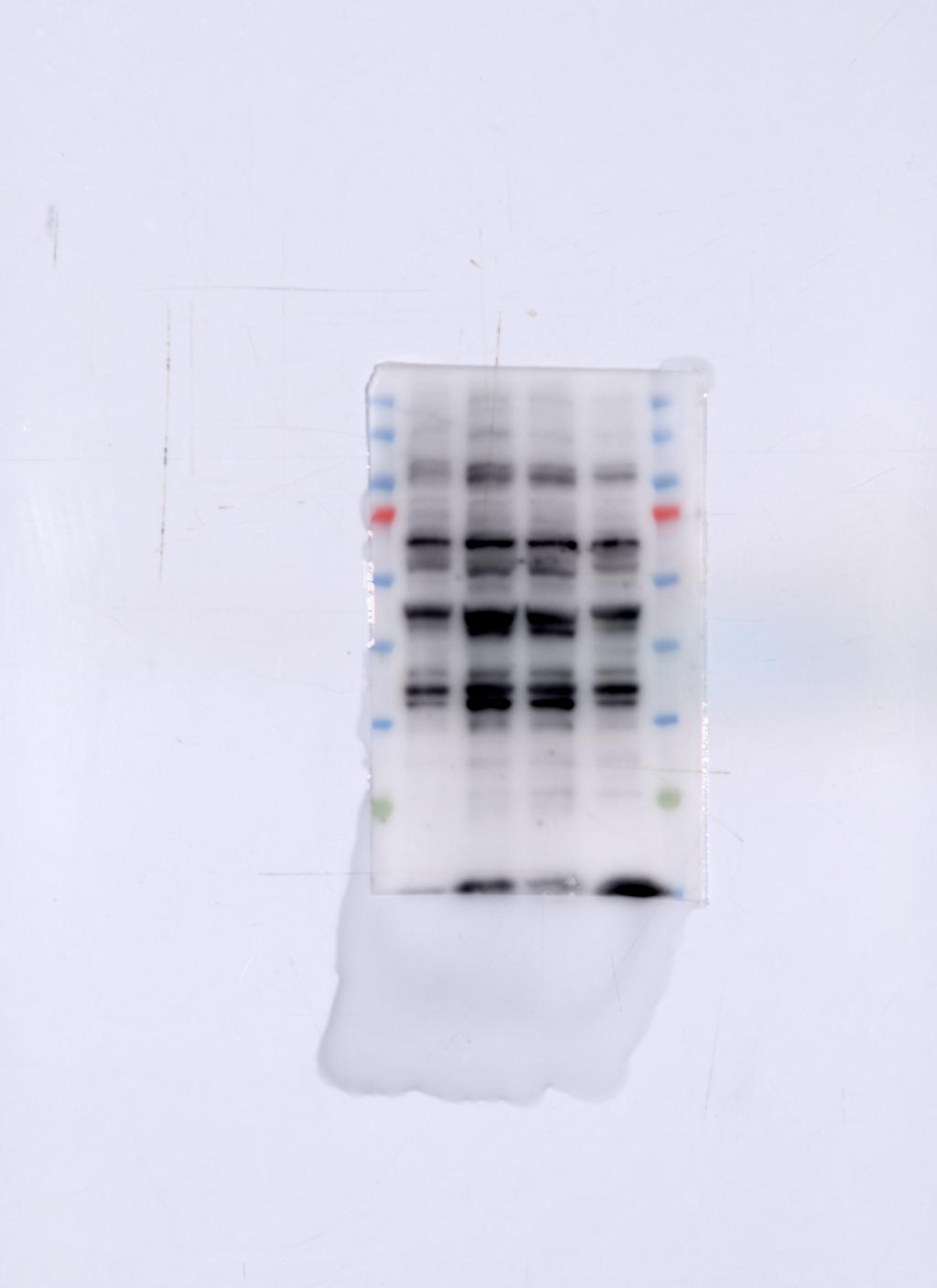

Supplement: Supplementary file 13 — Supplementary Material 13. [file 13046_2025_3438_MOESM13_ESM.zip › full uncropped Gels and Blots image/Fig1J-PanNmyr-blots.jpg]

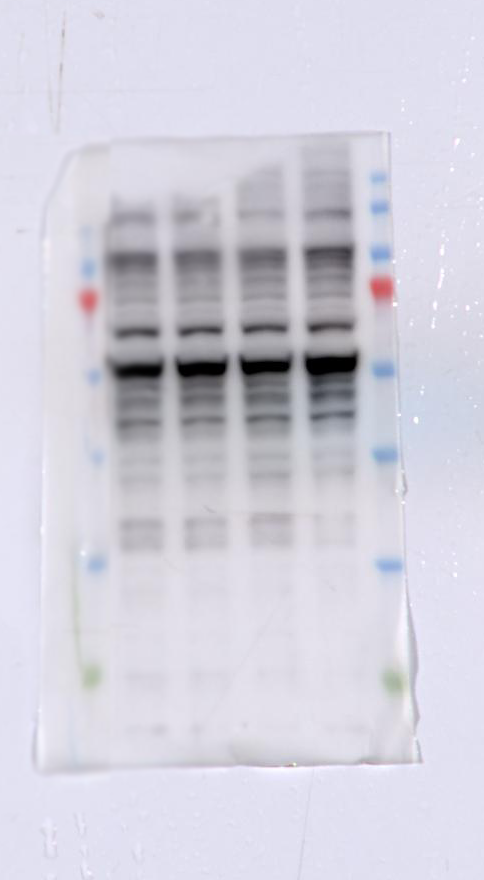

Supplement: Supplementary file 13 — Supplementary Material 13. [file 13046_2025_3438_MOESM13_ESM.zip › full uncropped Gels and Blots image/Fig1J-actin-blots.tif]

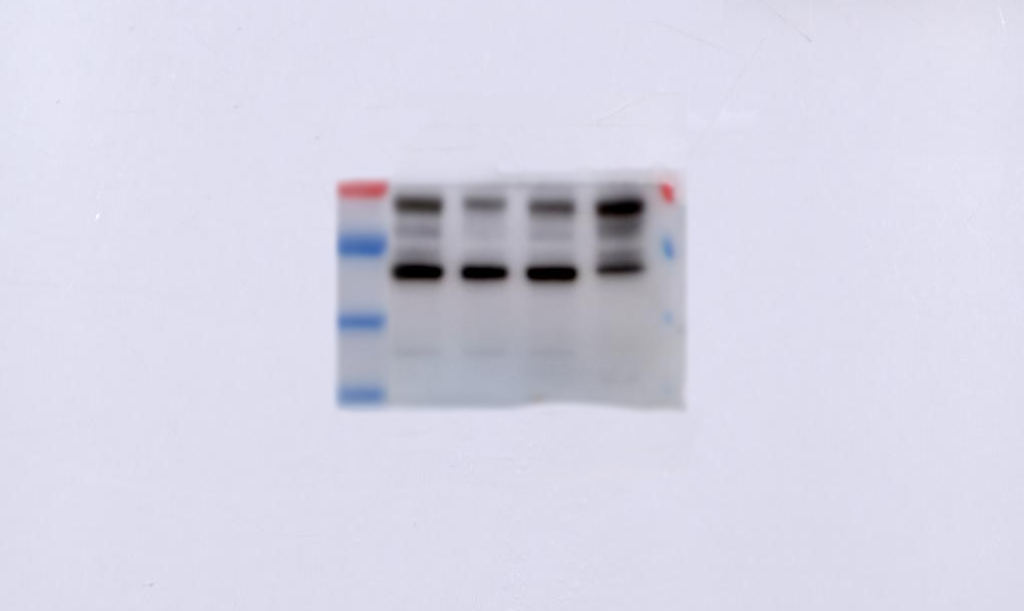

Supplement: Supplementary file 13 — Supplementary Material 13. [file 13046_2025_3438_MOESM13_ESM.zip › full uncropped Gels and Blots image/Fig3E-NMT1-blots.tif]

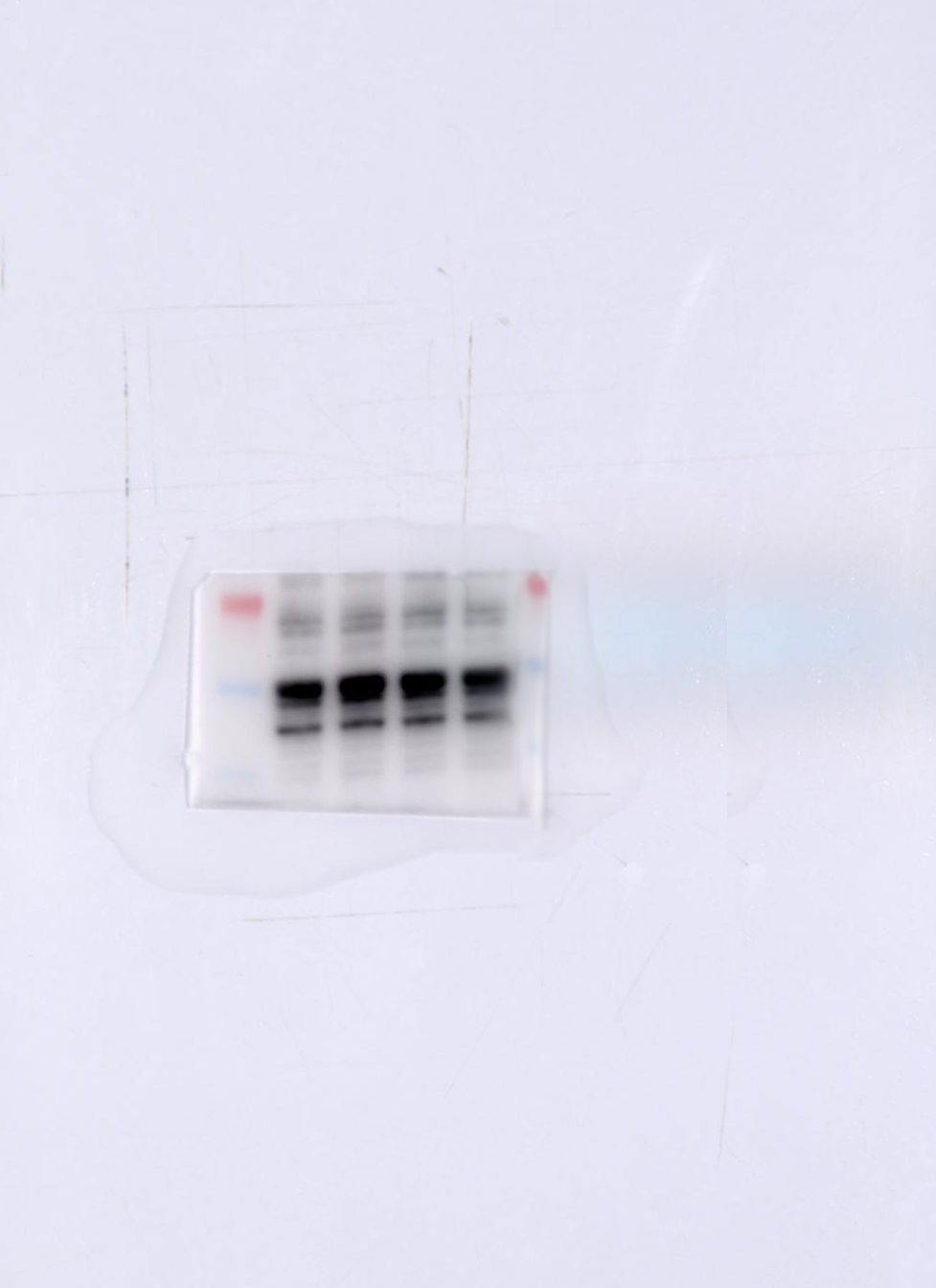

Supplement: Supplementary file 13 — Supplementary Material 13. [file 13046_2025_3438_MOESM13_ESM.zip › full uncropped Gels and Blots image/Fig3E-PD-L1-blot.tif]

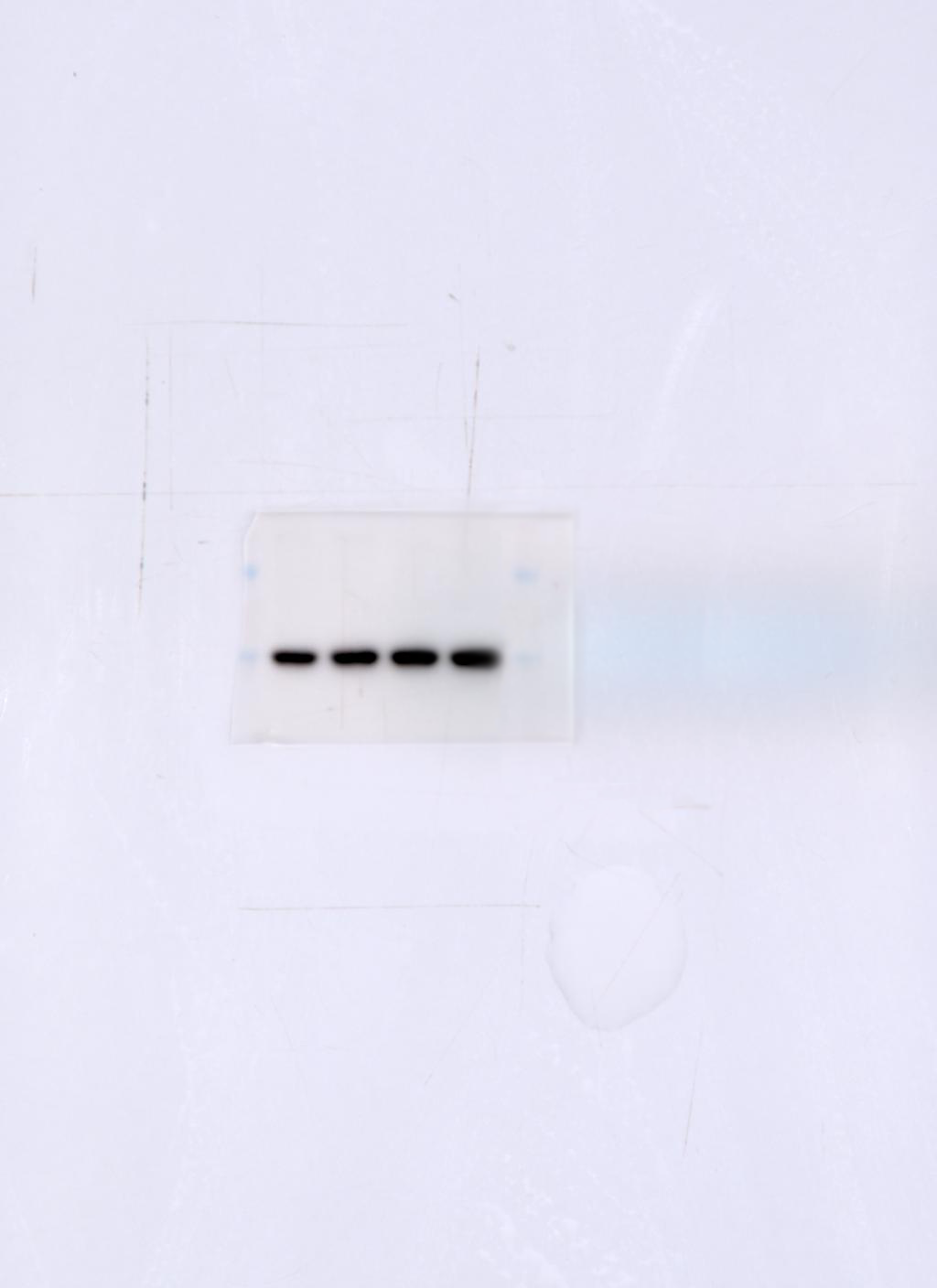

Supplement: Supplementary file 13 — Supplementary Material 13. [file 13046_2025_3438_MOESM13_ESM.zip › full uncropped Gels and Blots image/Fig3E-actin-blots.tif]

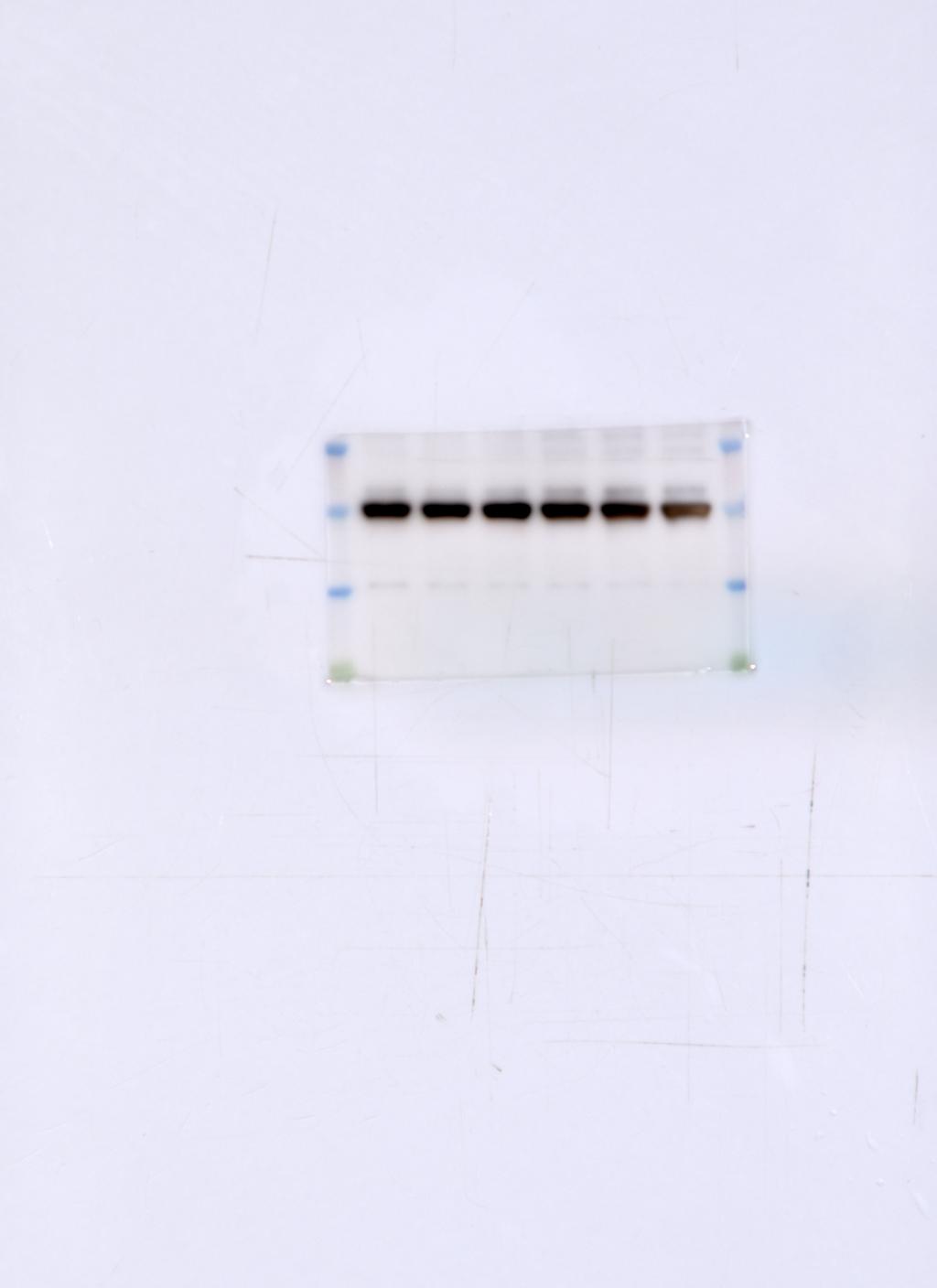

Supplement: Supplementary file 13 — Supplementary Material 13. [file 13046_2025_3438_MOESM13_ESM.zip › full uncropped Gels and Blots image/Fig3K-KYSE510-ACTIN.jpg]

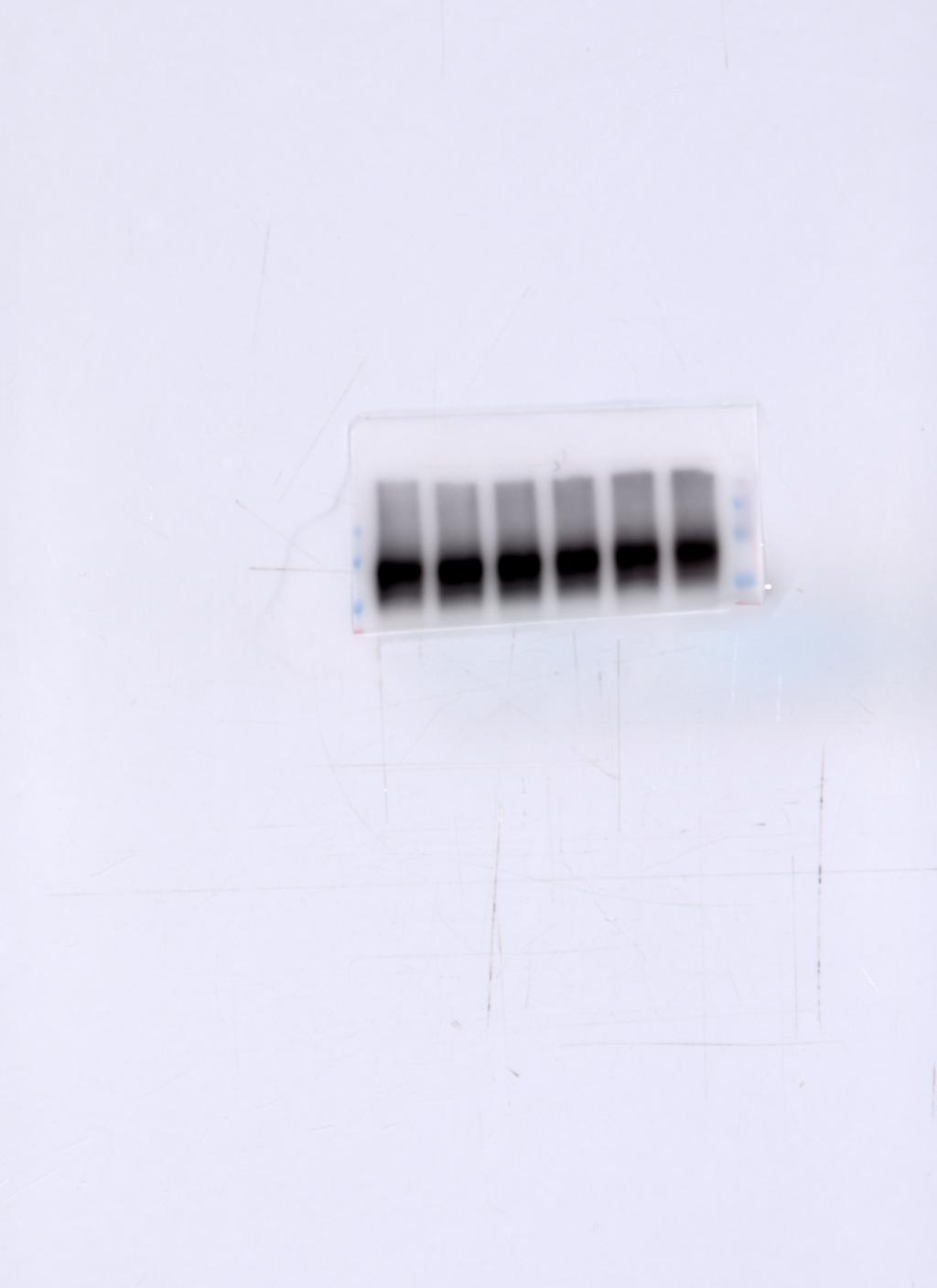

Supplement: Supplementary file 13 — Supplementary Material 13. [file 13046_2025_3438_MOESM13_ESM.zip › full uncropped Gels and Blots image/Fig3K-KYSE510-NaKatpase.jpg]

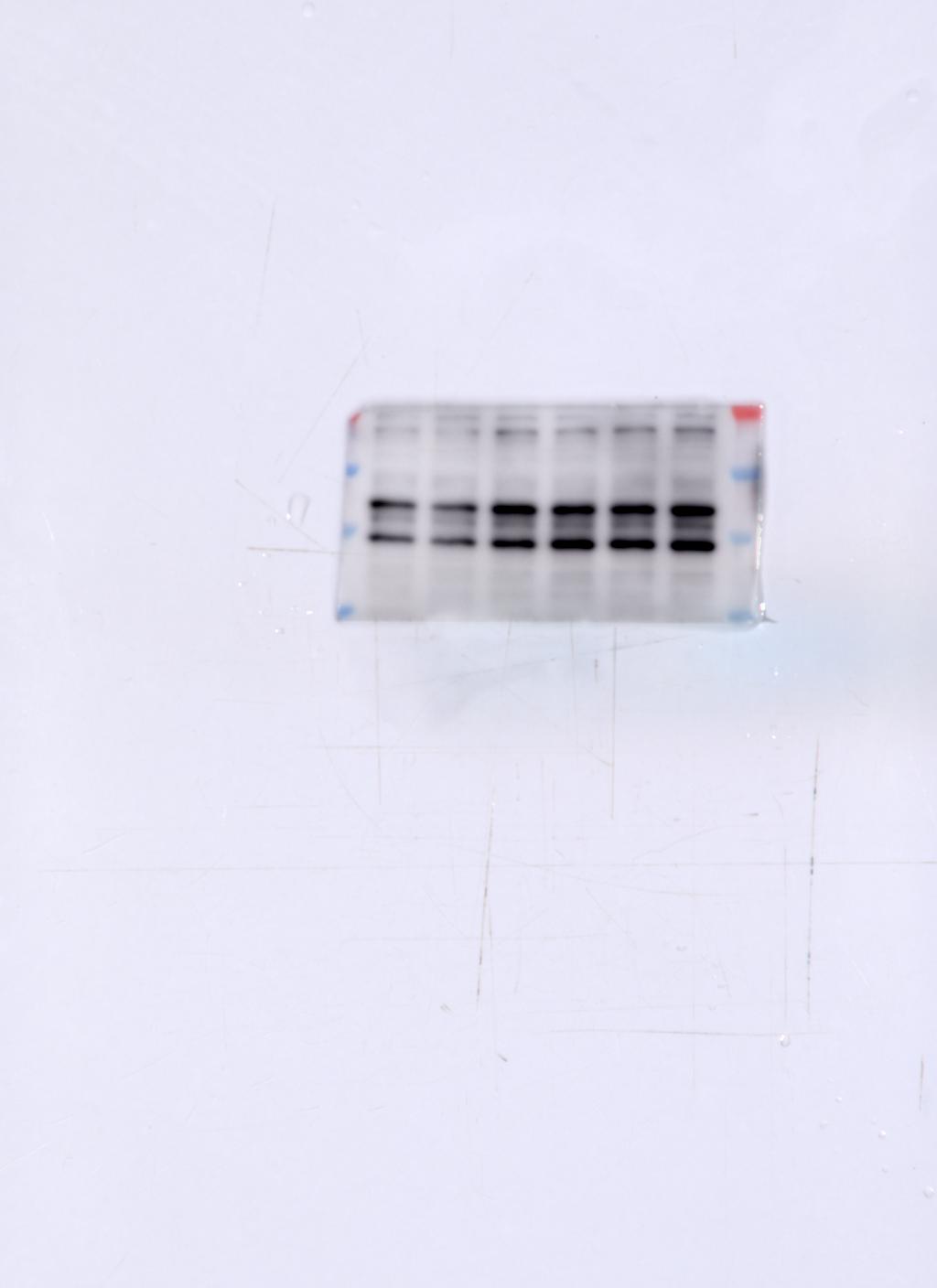

Supplement: Supplementary file 13 — Supplementary Material 13. [file 13046_2025_3438_MOESM13_ESM.zip › full uncropped Gels and Blots image/Fig3K-KYSE510-PD-L1-CM.jpg]

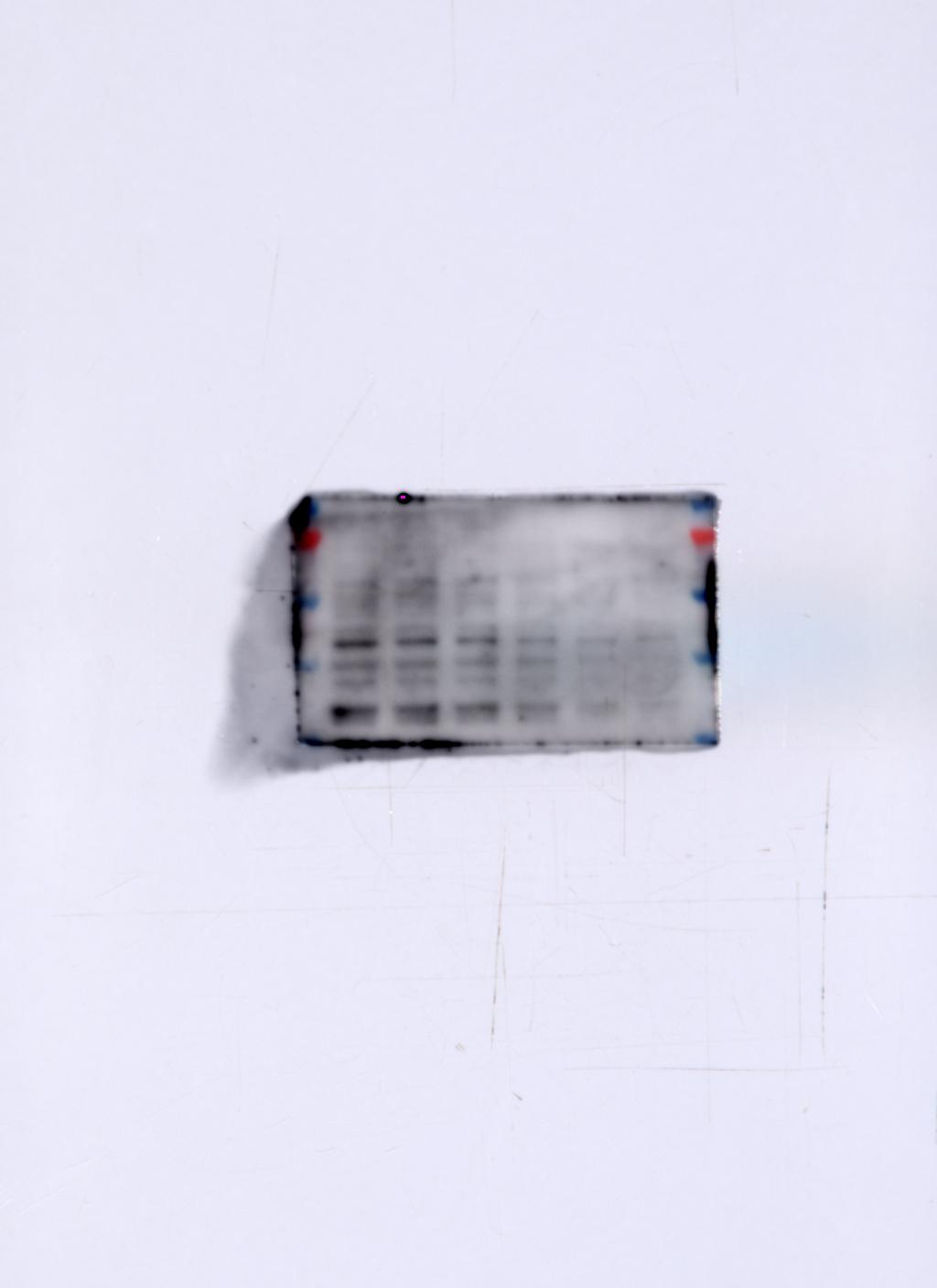

Supplement: Supplementary file 13 — Supplementary Material 13. [file 13046_2025_3438_MOESM13_ESM.zip › full uncropped Gels and Blots image/Fig3K-KYSE510-PD-L1-CP.jpg]

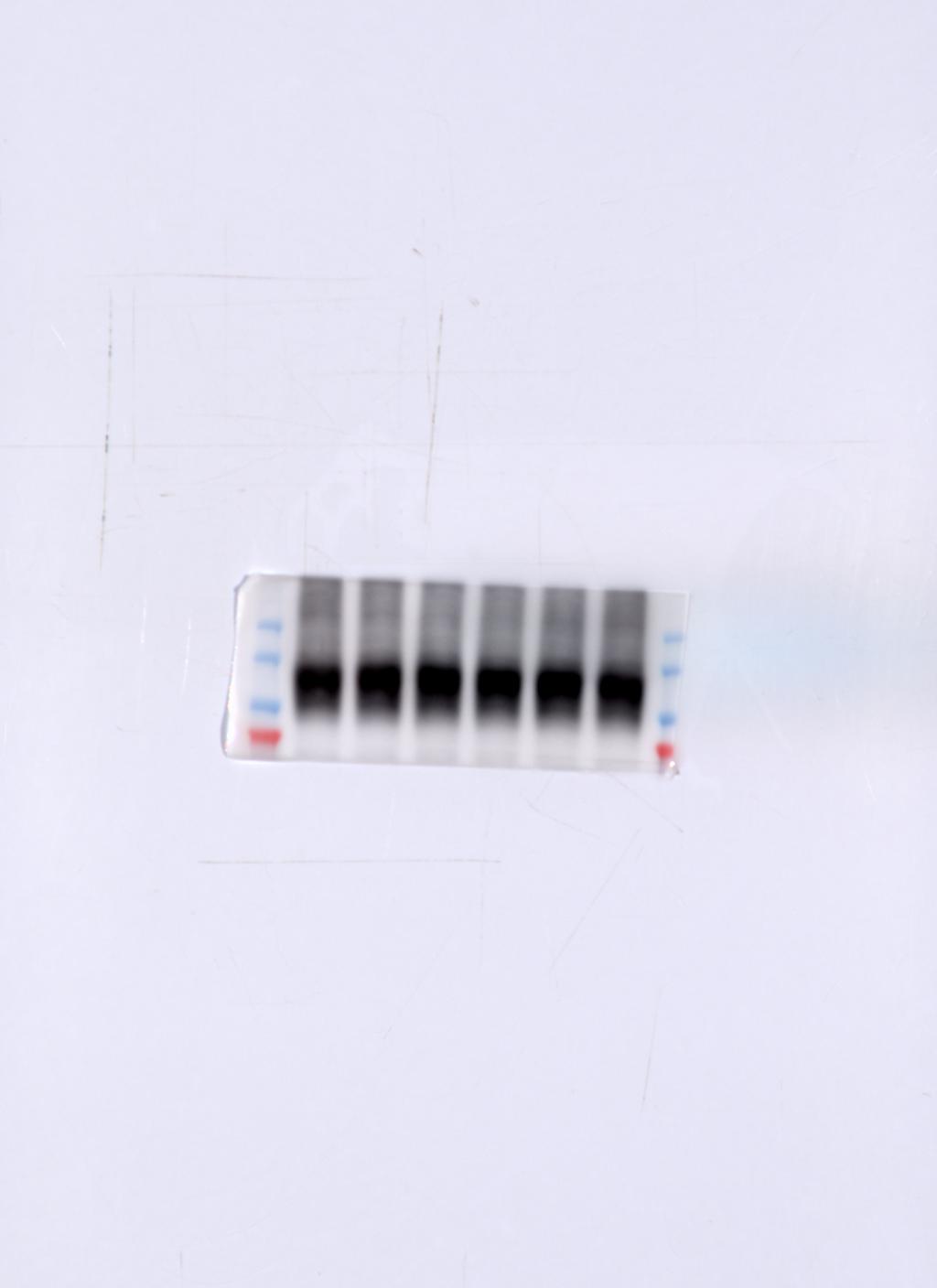

Supplement: Supplementary file 13 — Supplementary Material 13. [file 13046_2025_3438_MOESM13_ESM.zip › full uncropped Gels and Blots image/Fig3K-SCC9-NaKatpase.jpg]

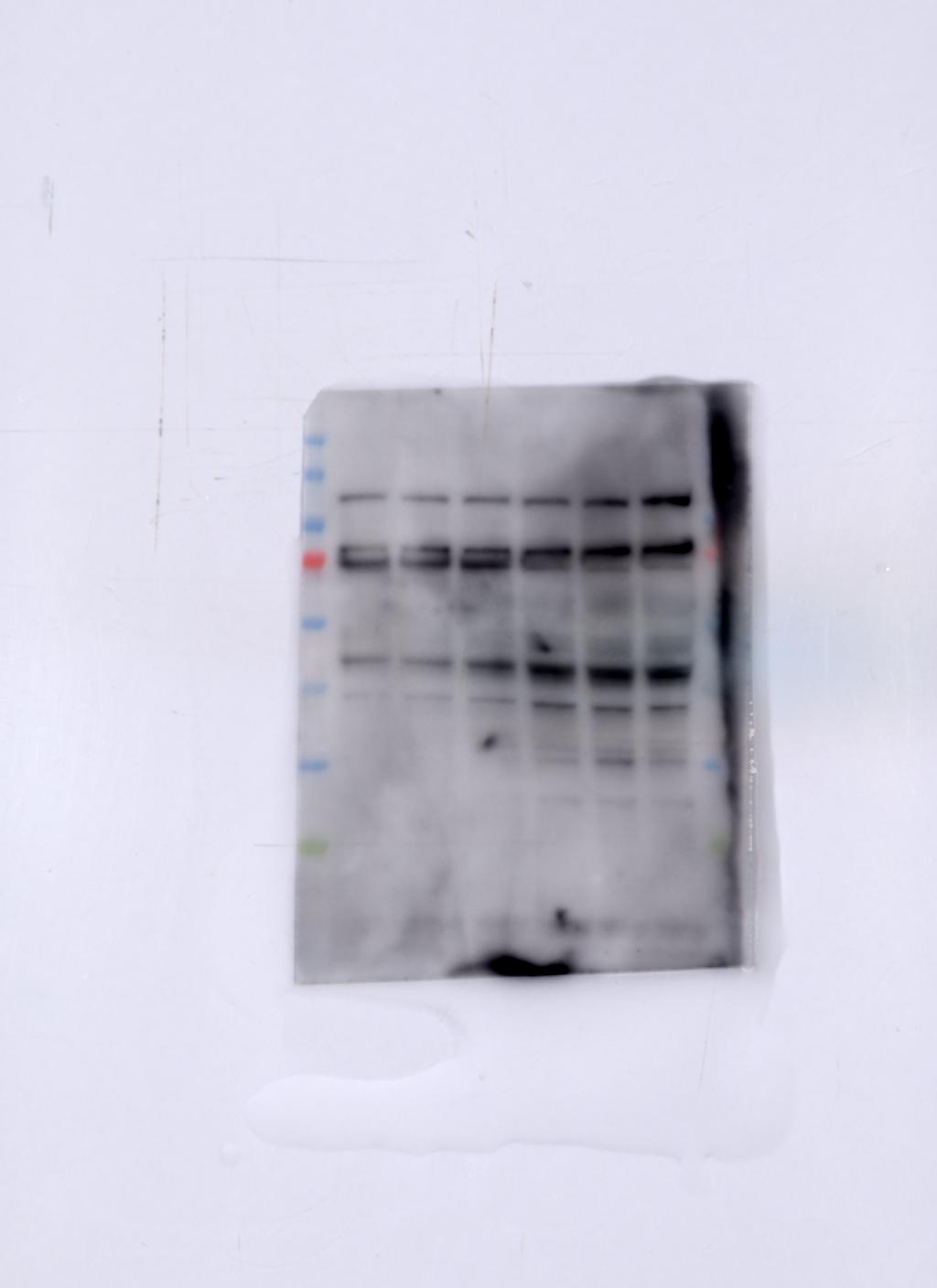

Supplement: Supplementary file 13 — Supplementary Material 13. [file 13046_2025_3438_MOESM13_ESM.zip › full uncropped Gels and Blots image/Fig3K-SCC9-PD-L1-CM.jpg]

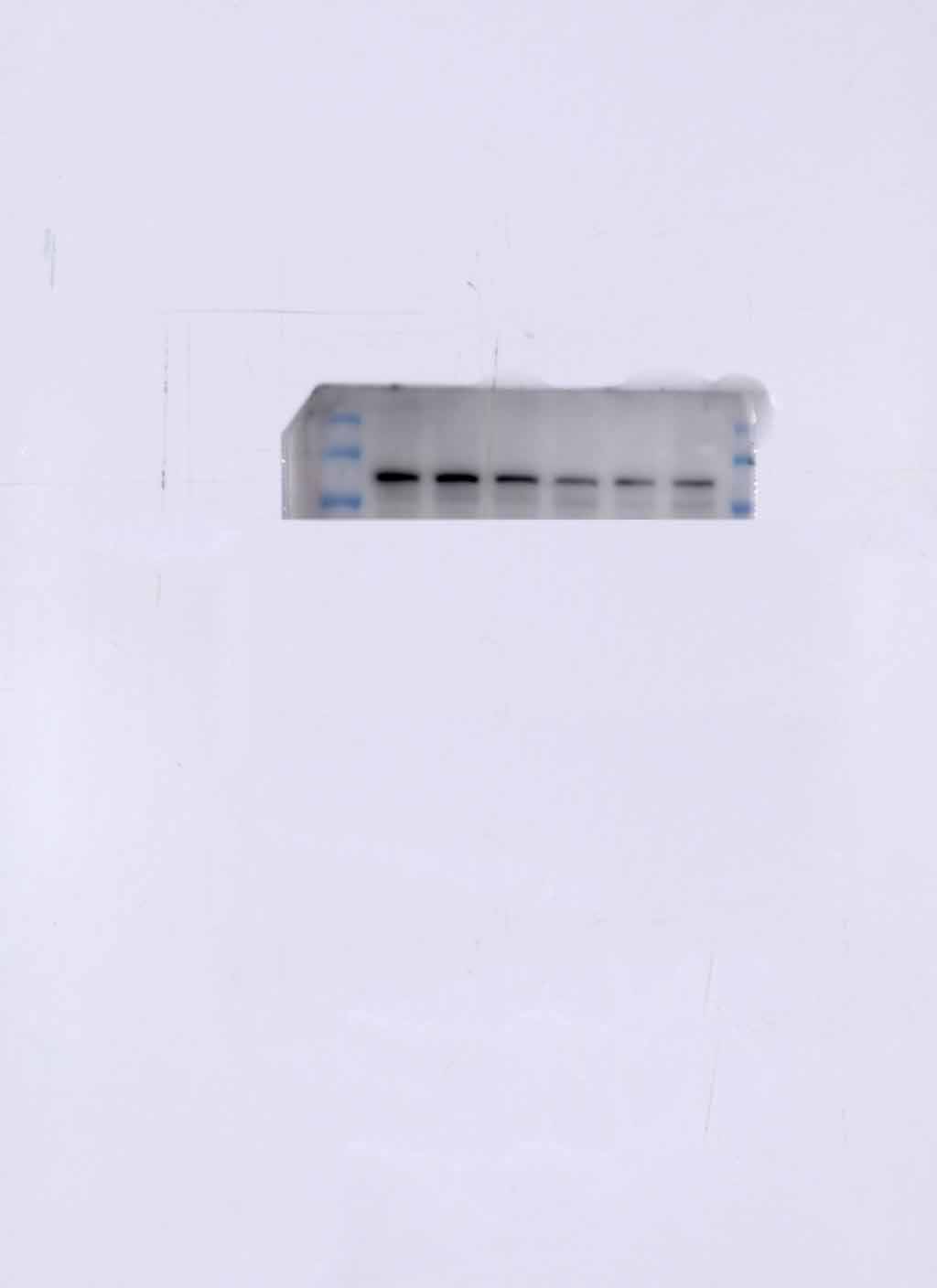

Supplement: Supplementary file 13 — Supplementary Material 13. [file 13046_2025_3438_MOESM13_ESM.zip › full uncropped Gels and Blots image/Fig3K-SCC9-PD-L1-CP.jpg]

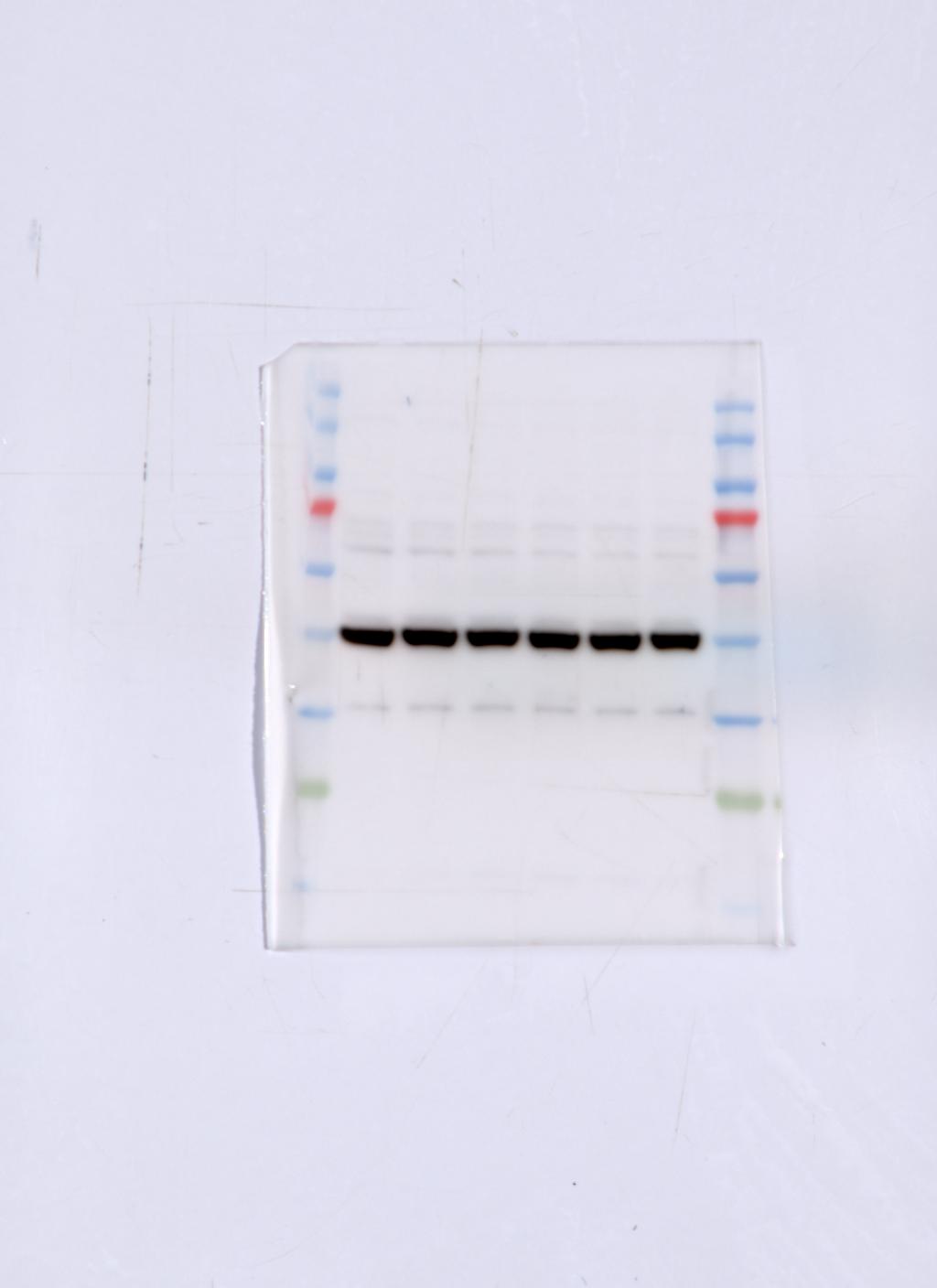

Supplement: Supplementary file 13 — Supplementary Material 13. [file 13046_2025_3438_MOESM13_ESM.zip › full uncropped Gels and Blots image/Fig3K-SCC9-actin.jpg]

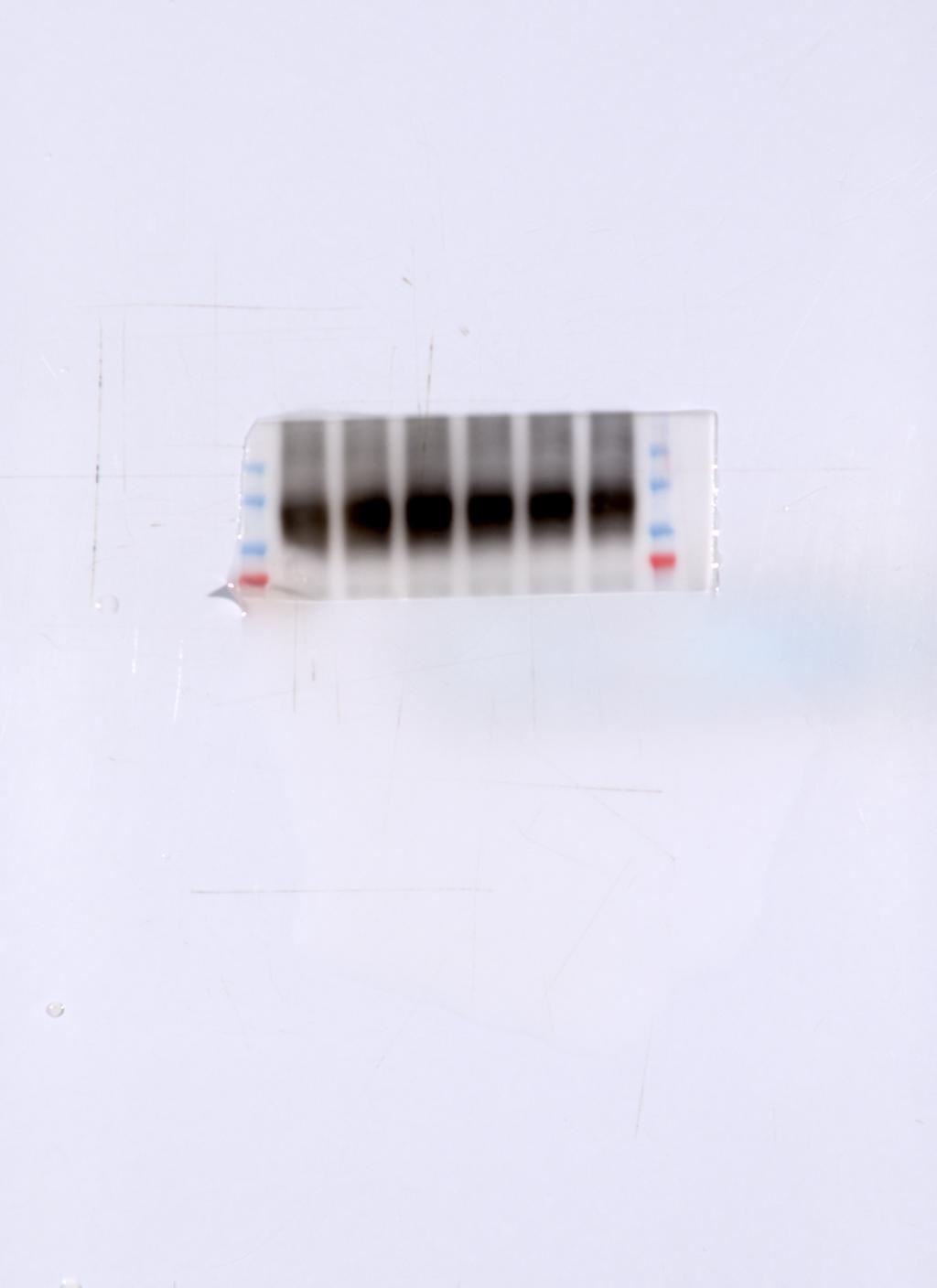

Supplement: Supplementary file 13 — Supplementary Material 13. [file 13046_2025_3438_MOESM13_ESM.zip › full uncropped Gels and Blots image/Fig3K-SIHa-NaKatpase.jpg]

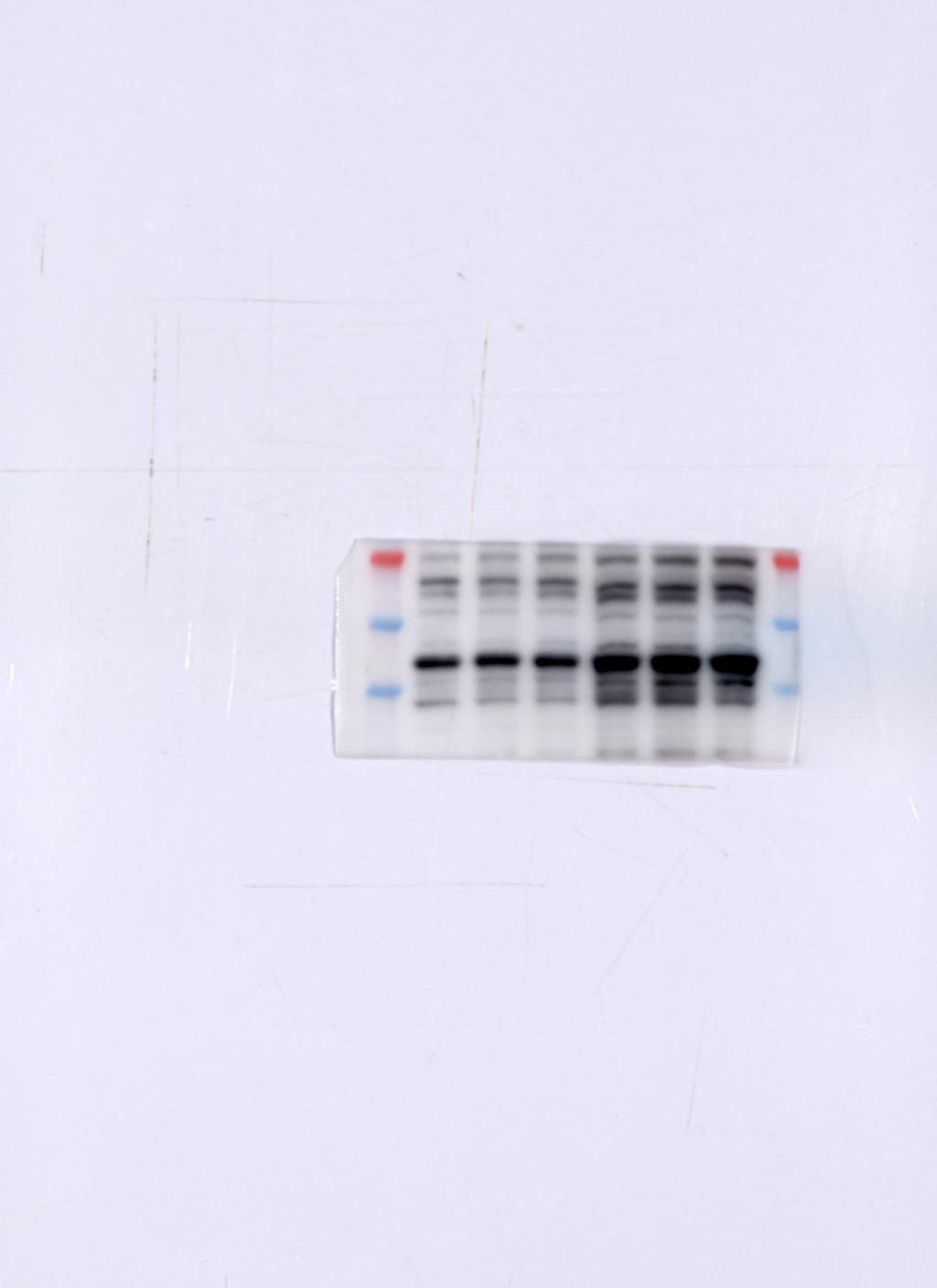

Supplement: Supplementary file 13 — Supplementary Material 13. [file 13046_2025_3438_MOESM13_ESM.zip › full uncropped Gels and Blots image/Fig3K-SIHa-PD-L1-CM.jpg]

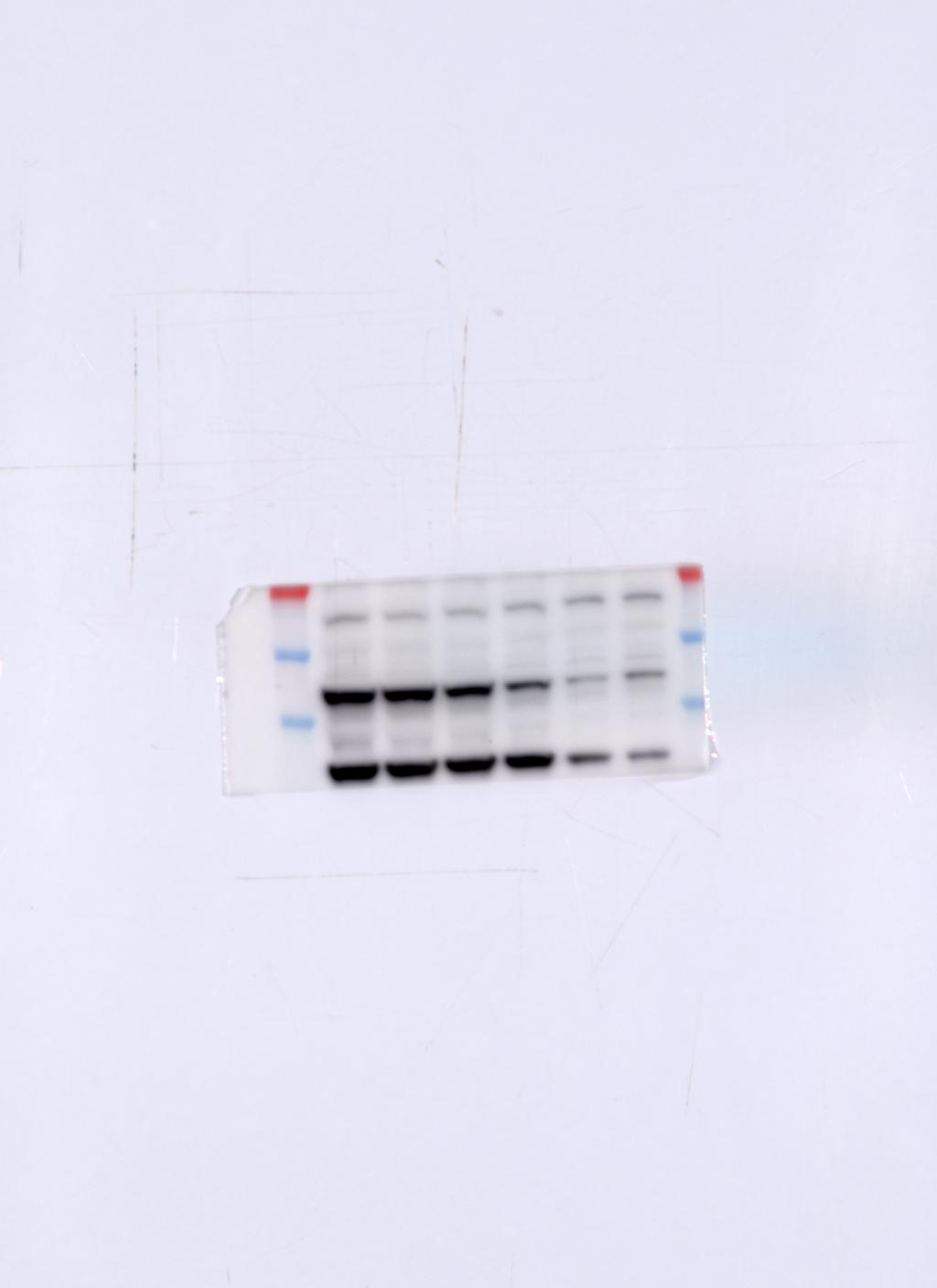

Supplement: Supplementary file 13 — Supplementary Material 13. [file 13046_2025_3438_MOESM13_ESM.zip › full uncropped Gels and Blots image/Fig3K-SIHa-PD-L1-CP.jpg]

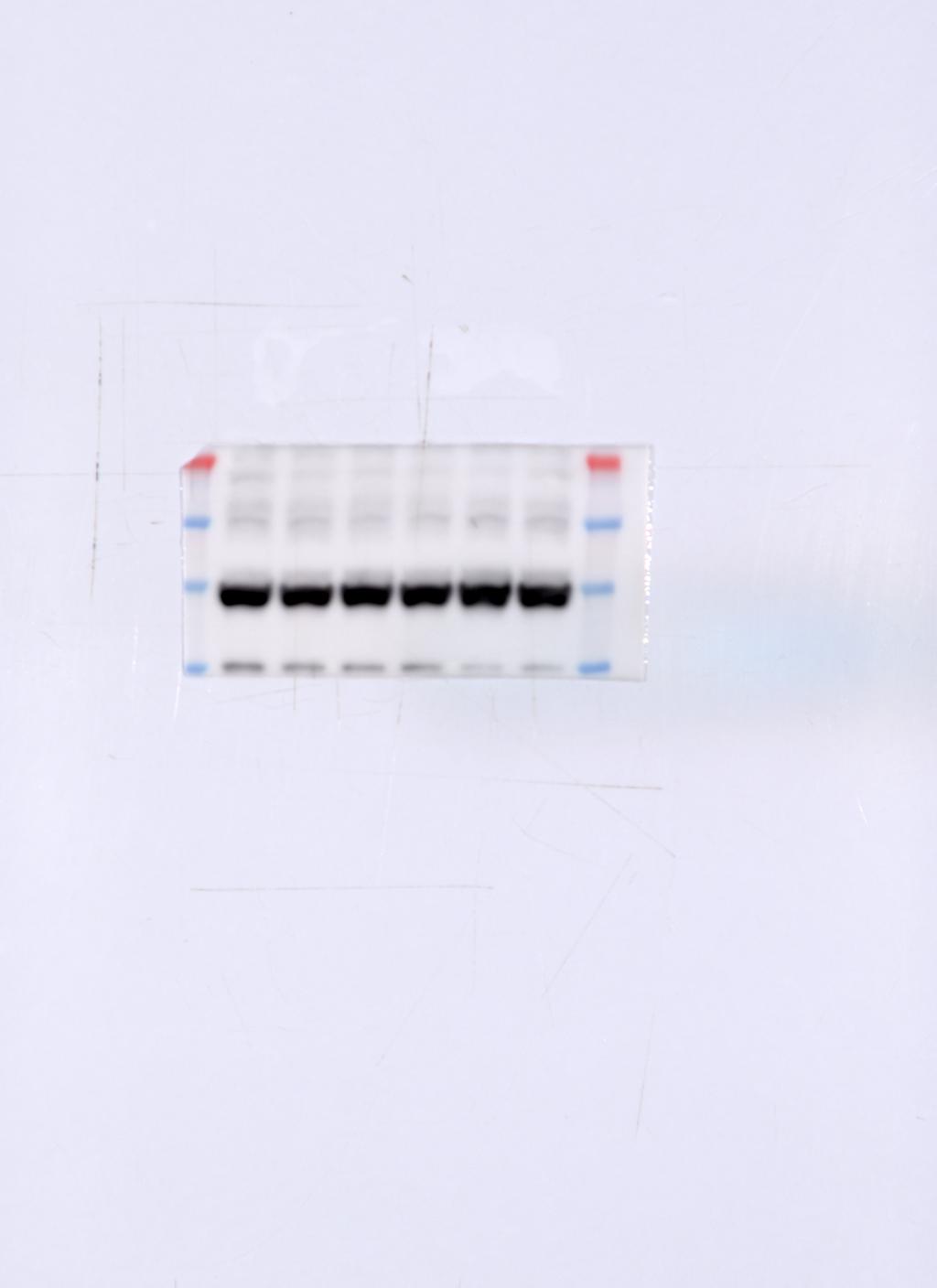

Supplement: Supplementary file 13 — Supplementary Material 13. [file 13046_2025_3438_MOESM13_ESM.zip › full uncropped Gels and Blots image/Fig3K-SIHa-actin.jpg]

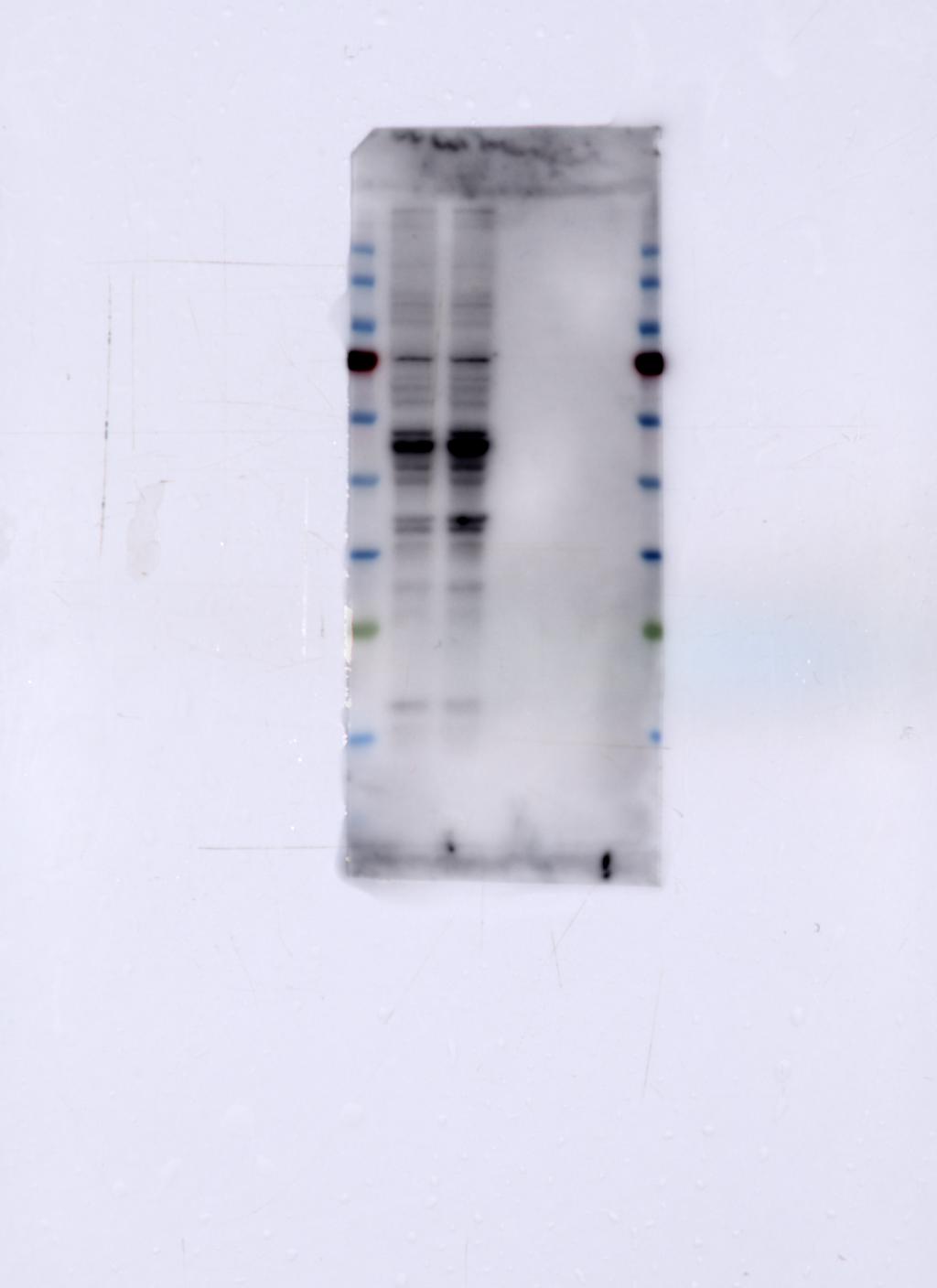

Supplement: Supplementary file 13 — Supplementary Material 13. [file 13046_2025_3438_MOESM13_ESM.zip › full uncropped Gels and Blots image/Fig3P-PD-L1-blots.jpg]

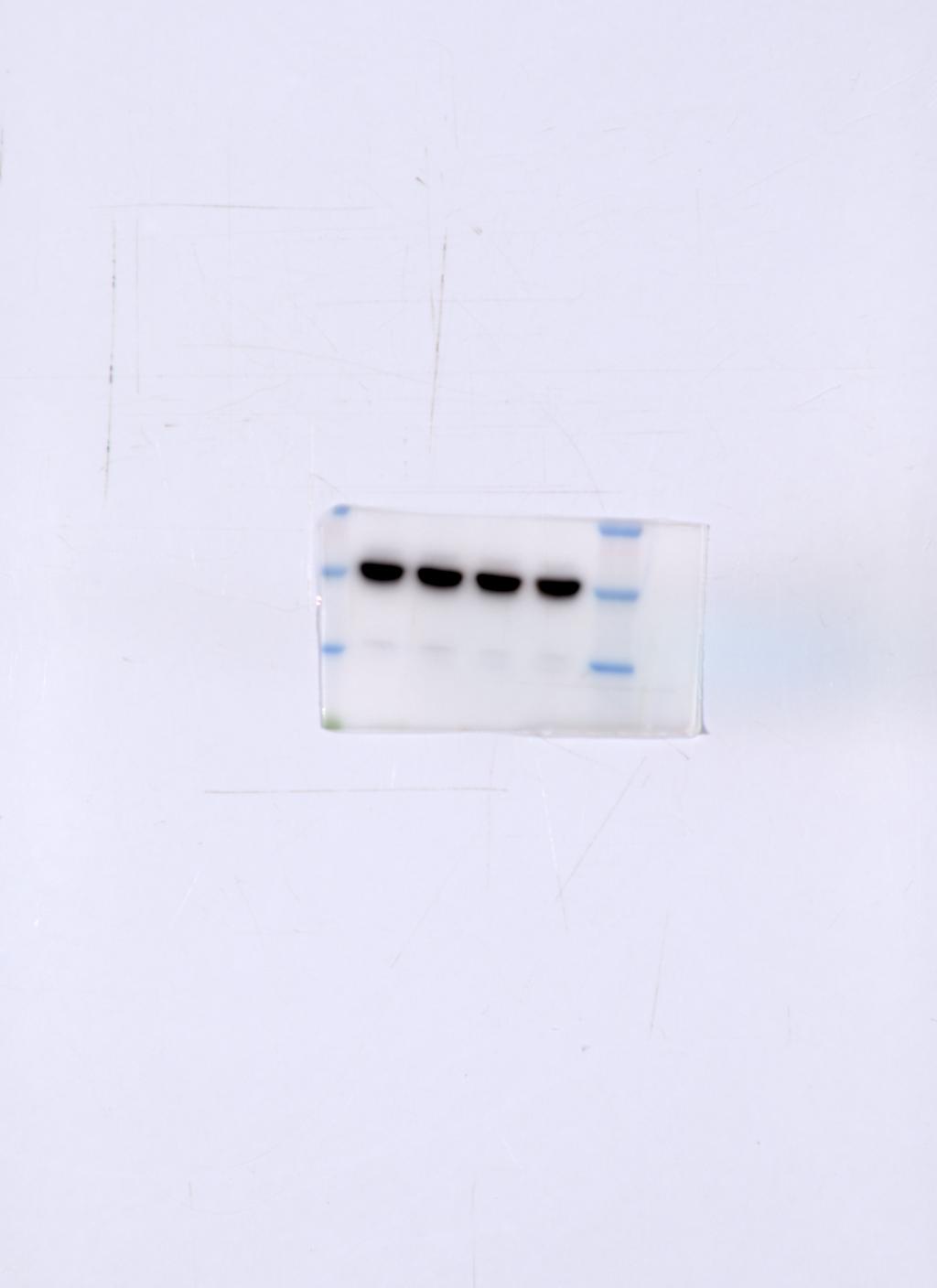

Supplement: Supplementary file 13 — Supplementary Material 13. [file 13046_2025_3438_MOESM13_ESM.zip › full uncropped Gels and Blots image/Fig3P-actin-blots.jpg]

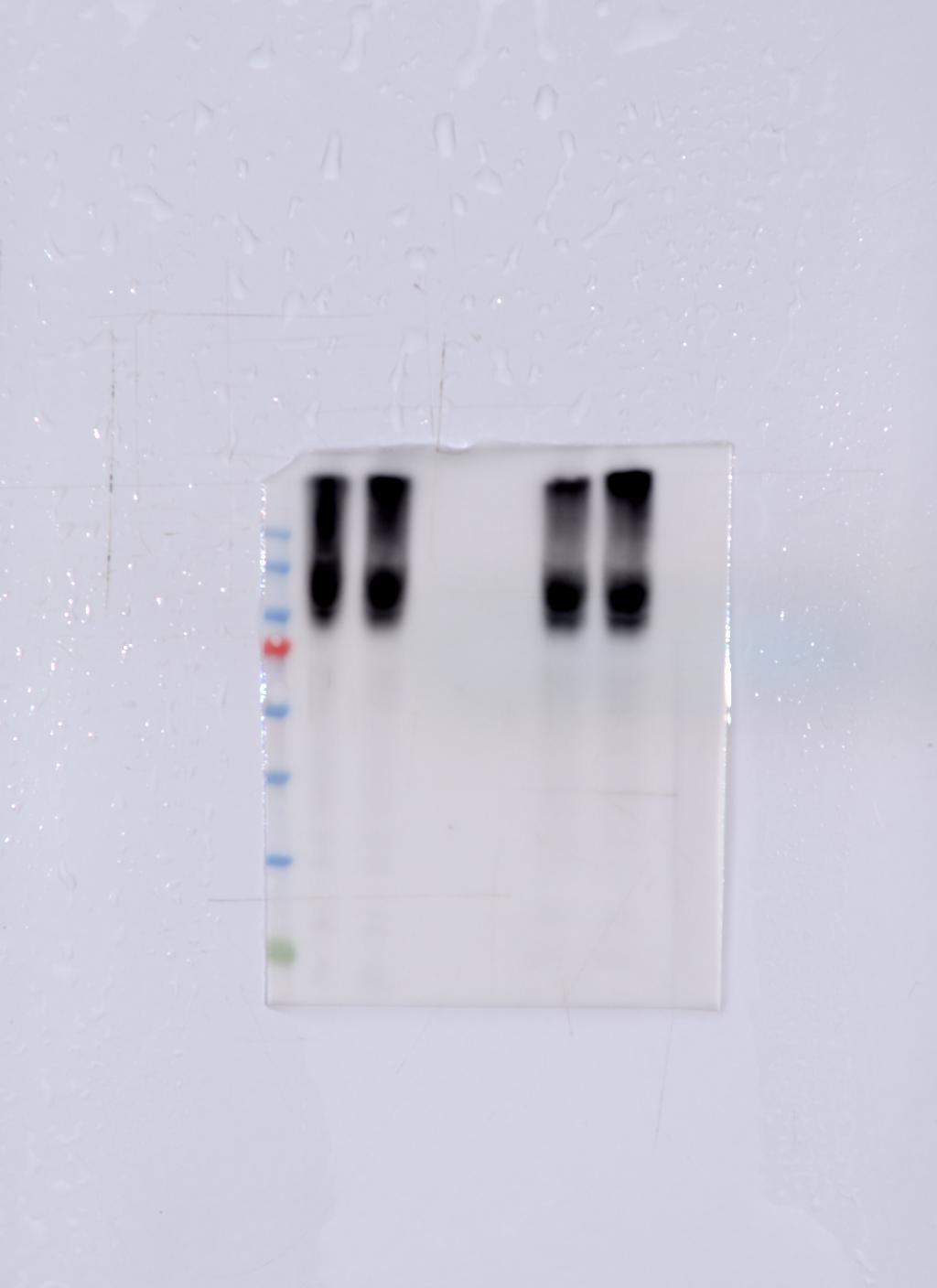

Supplement: Supplementary file 13 — Supplementary Material 13. [file 13046_2025_3438_MOESM13_ESM.zip › full uncropped Gels and Blots image/Fig4G-NaKatpase.jpg]

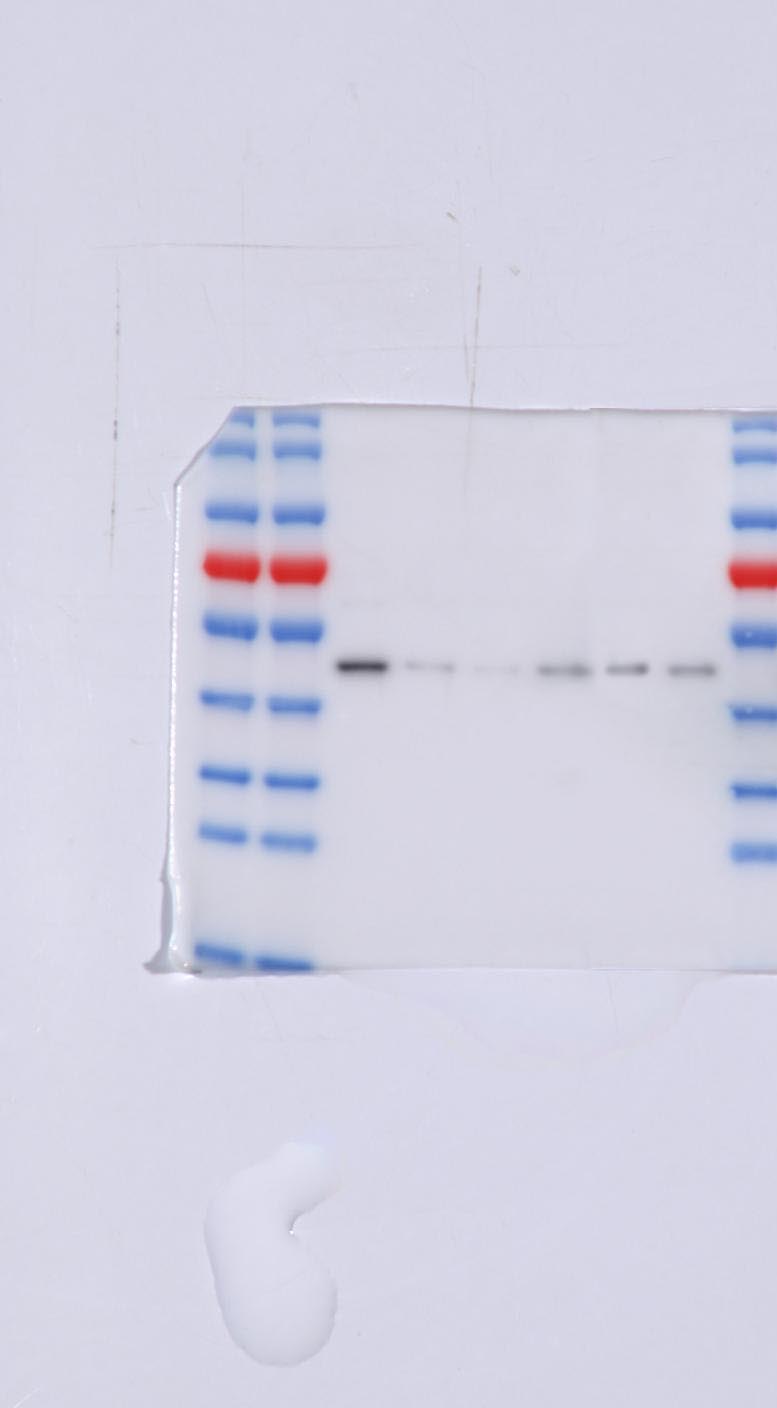

Supplement: Supplementary file 13 — Supplementary Material 13. [file 13046_2025_3438_MOESM13_ESM.zip › full uncropped Gels and Blots image/Fig4G-PD-L1-blots.jpg]

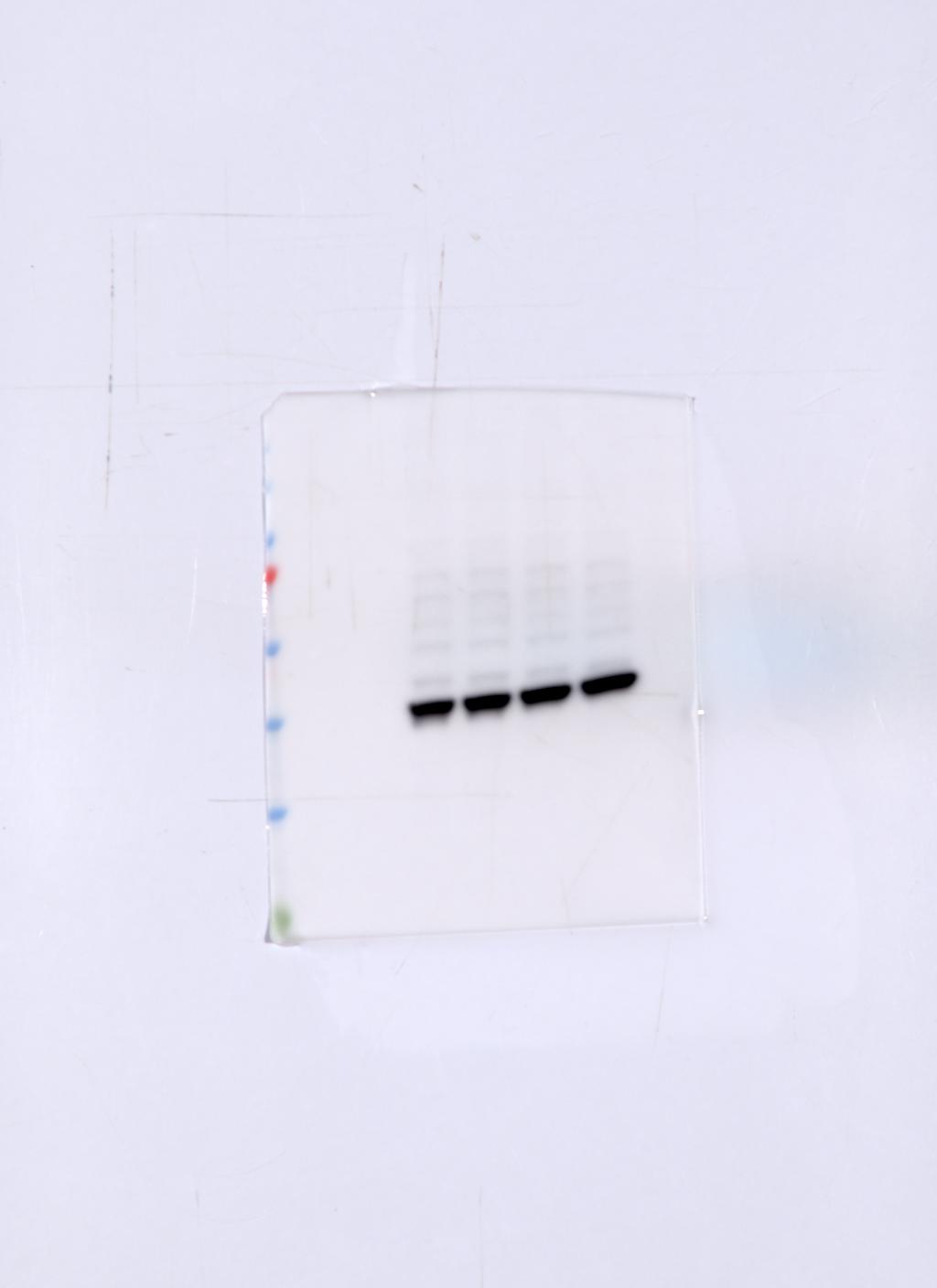

Supplement: Supplementary file 13 — Supplementary Material 13. [file 13046_2025_3438_MOESM13_ESM.zip › full uncropped Gels and Blots image/Fig4G-actin.jpg]

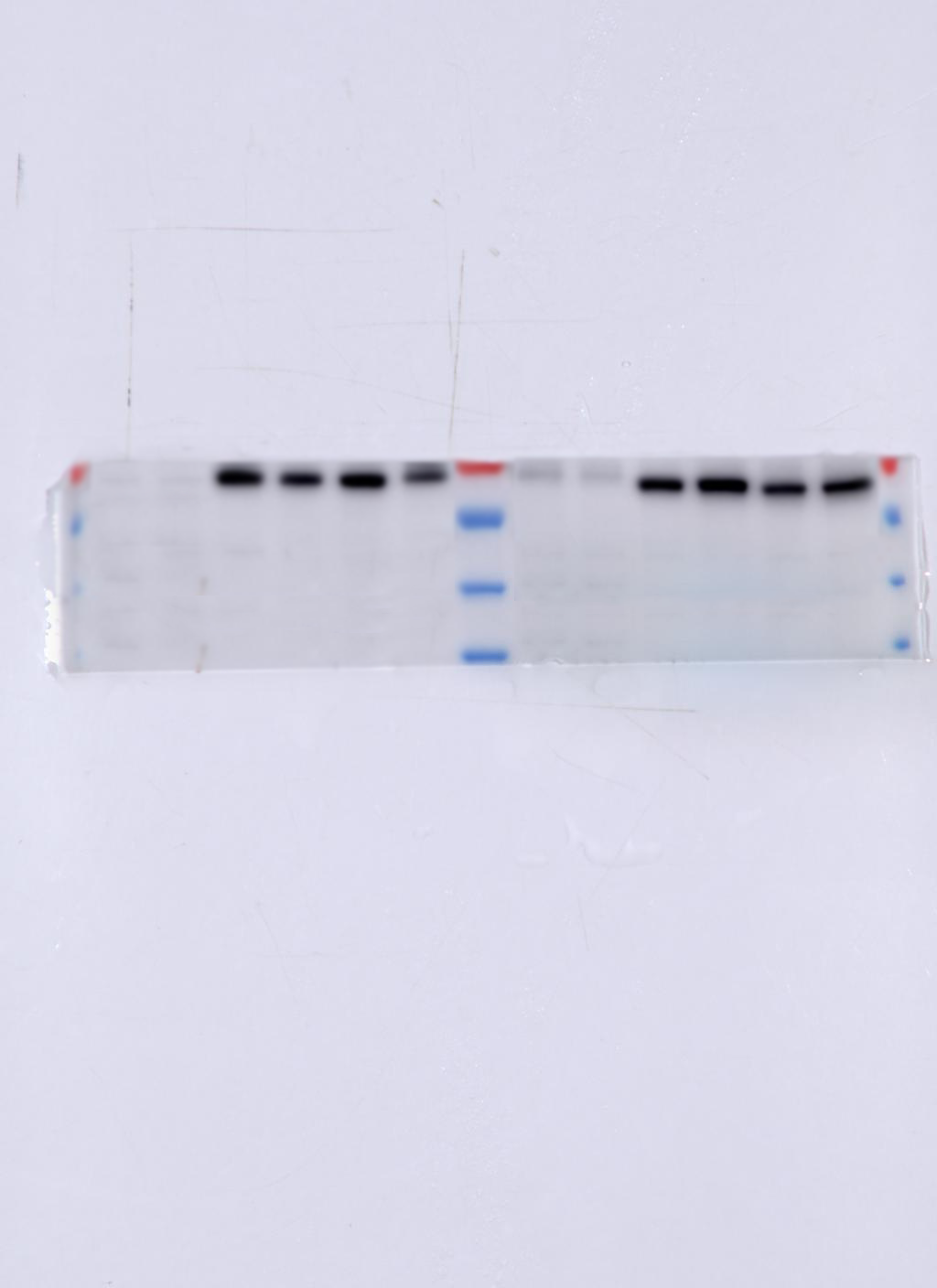

Supplement: Supplementary file 13 — Supplementary Material 13. [file 13046_2025_3438_MOESM13_ESM.zip › full uncropped Gels and Blots image/Fig4G&I-NMT1-blot.tif]

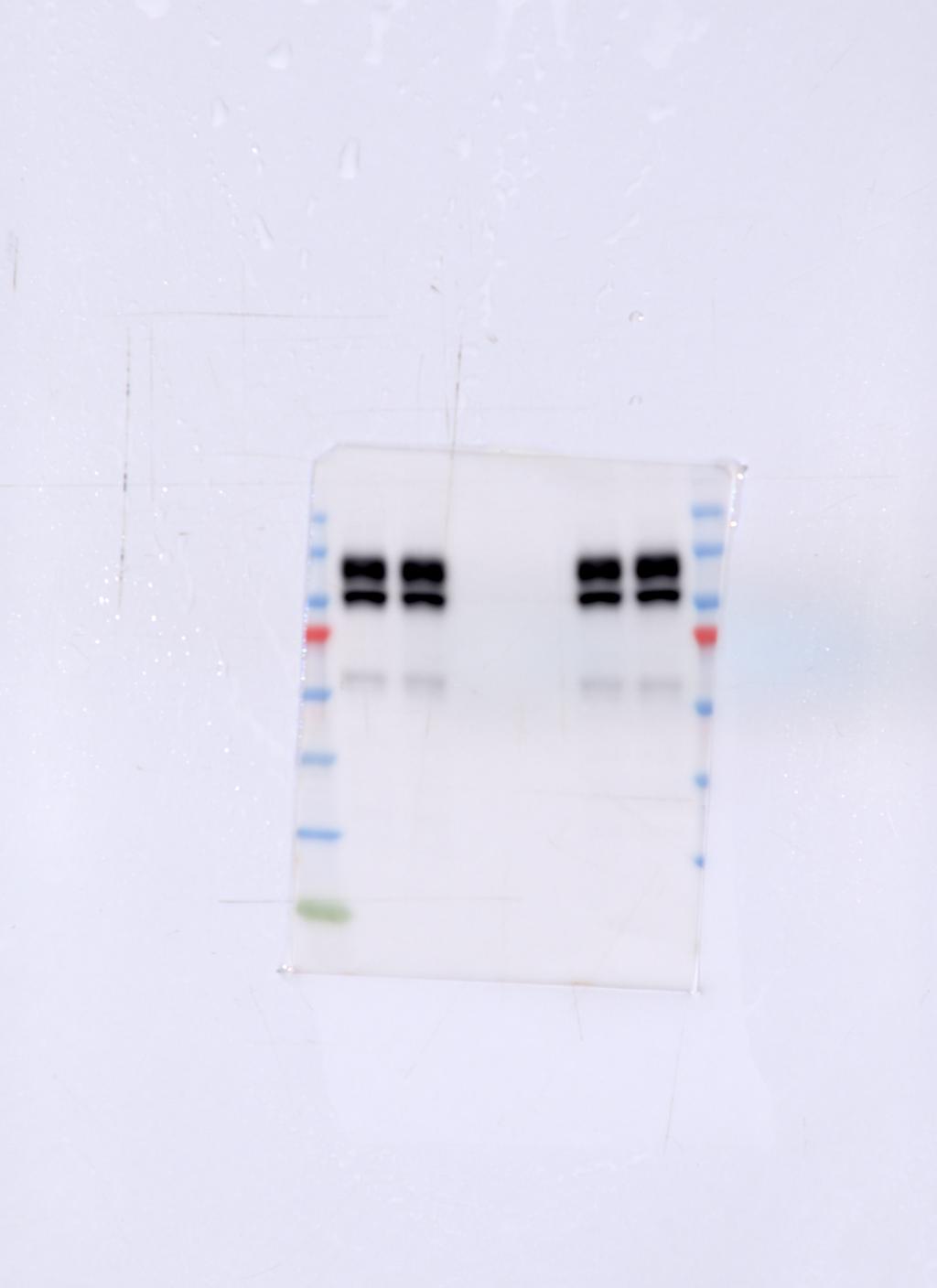

Supplement: Supplementary file 13 — Supplementary Material 13. [file 13046_2025_3438_MOESM13_ESM.zip › full uncropped Gels and Blots image/Fig4I-NaKatpase-blot.jpg]

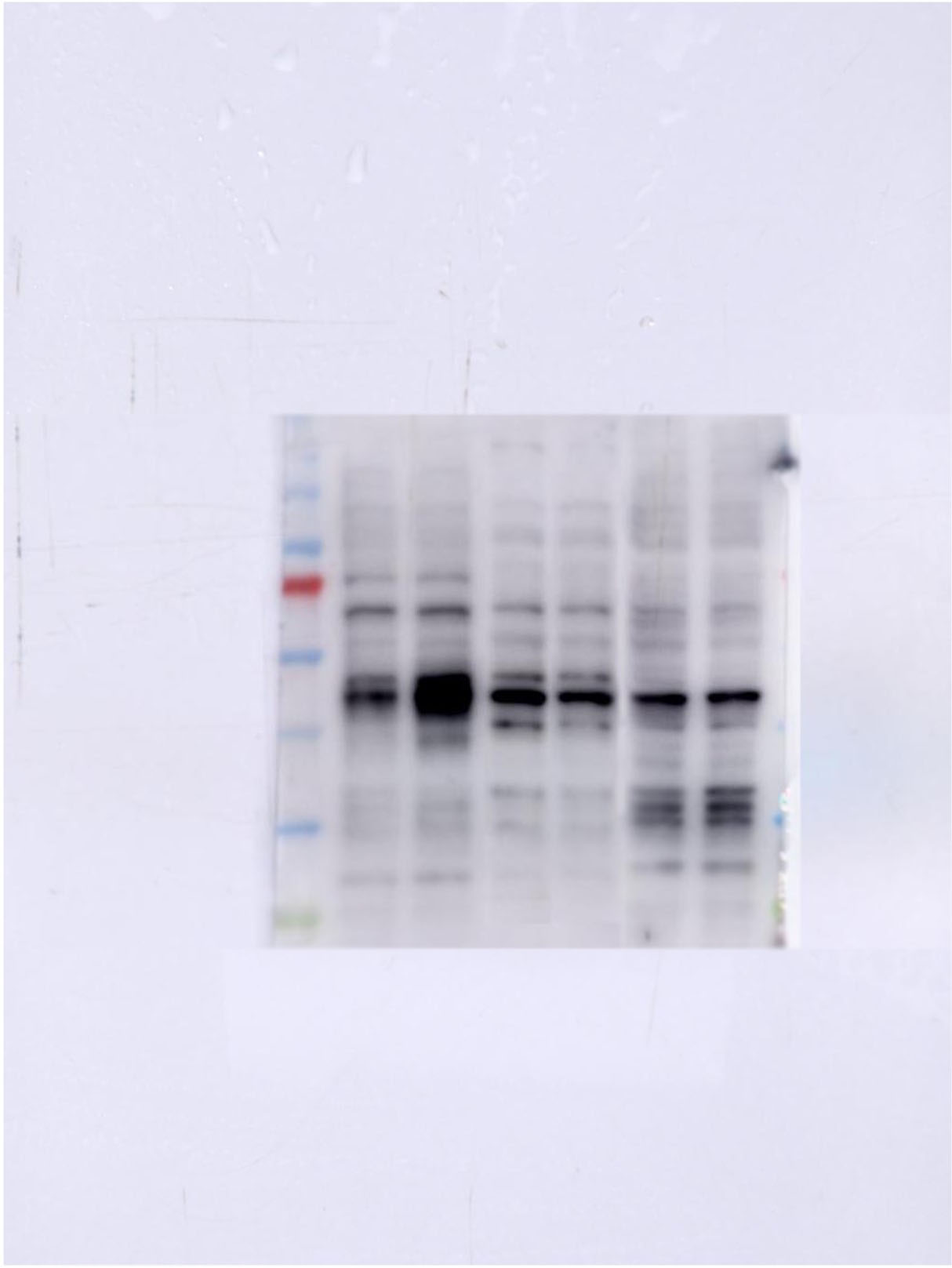

Supplement: Supplementary file 13 — Supplementary Material 13. [file 13046_2025_3438_MOESM13_ESM.zip › full uncropped Gels and Blots image/Fig4I-PD-L1-blots.jpg]

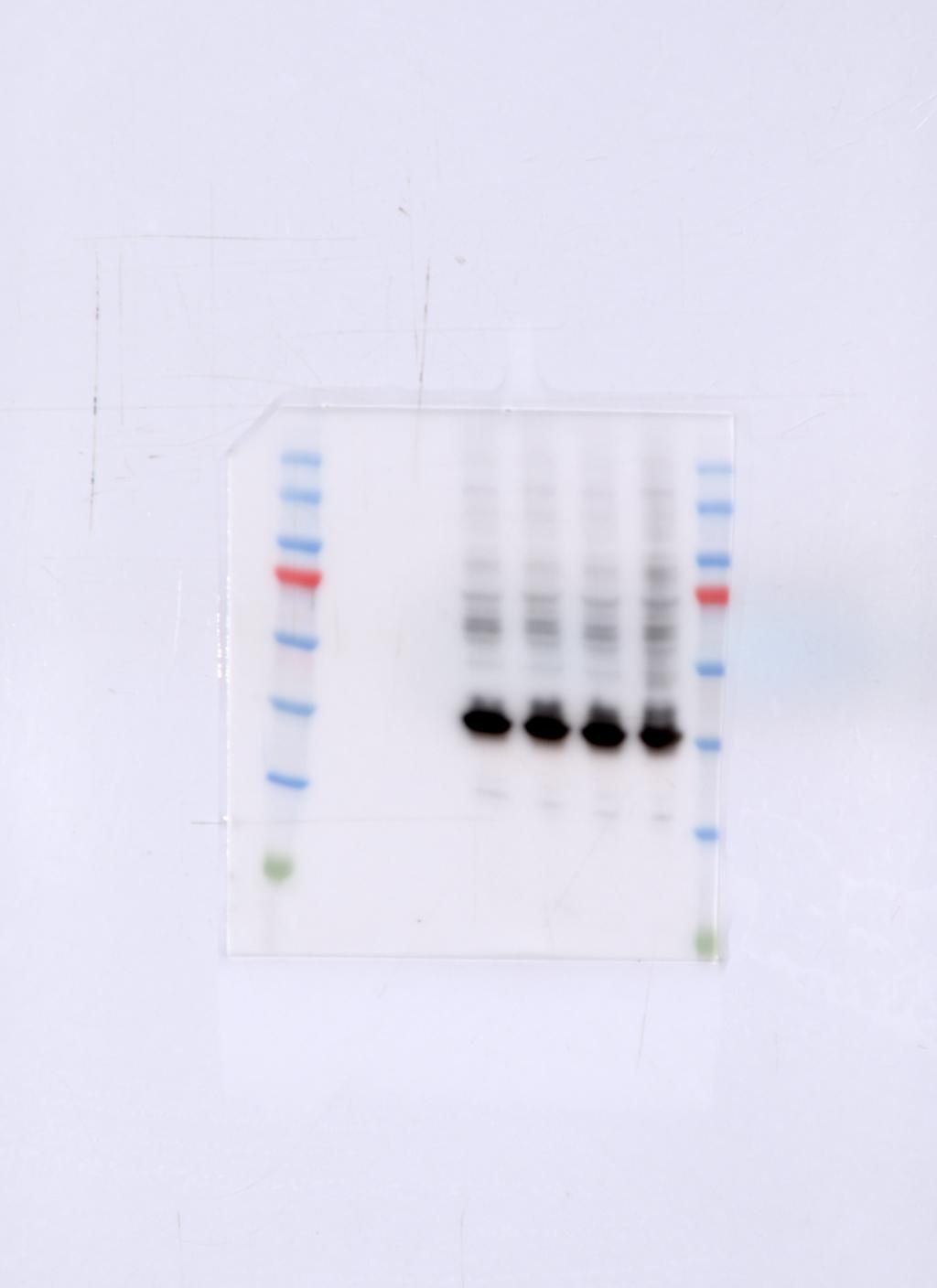

Supplement: Supplementary file 13 — Supplementary Material 13. [file 13046_2025_3438_MOESM13_ESM.zip › full uncropped Gels and Blots image/Fig4I-actin-blots.jpg]

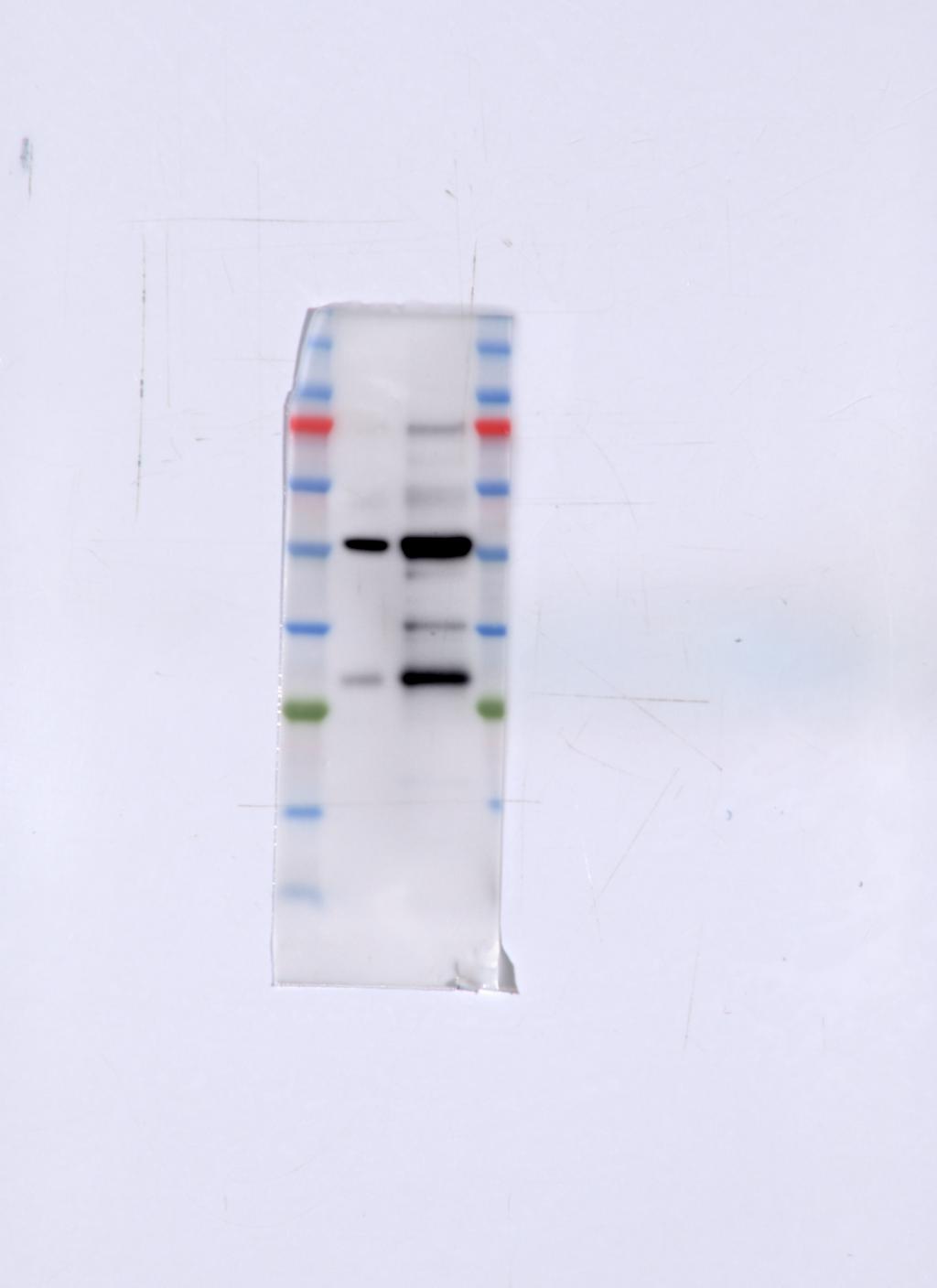

Supplement: Supplementary file 13 — Supplementary Material 13. [file 13046_2025_3438_MOESM13_ESM.zip › full uncropped Gels and Blots image/Fig5B-CHP-1-IP-blots.jpg]

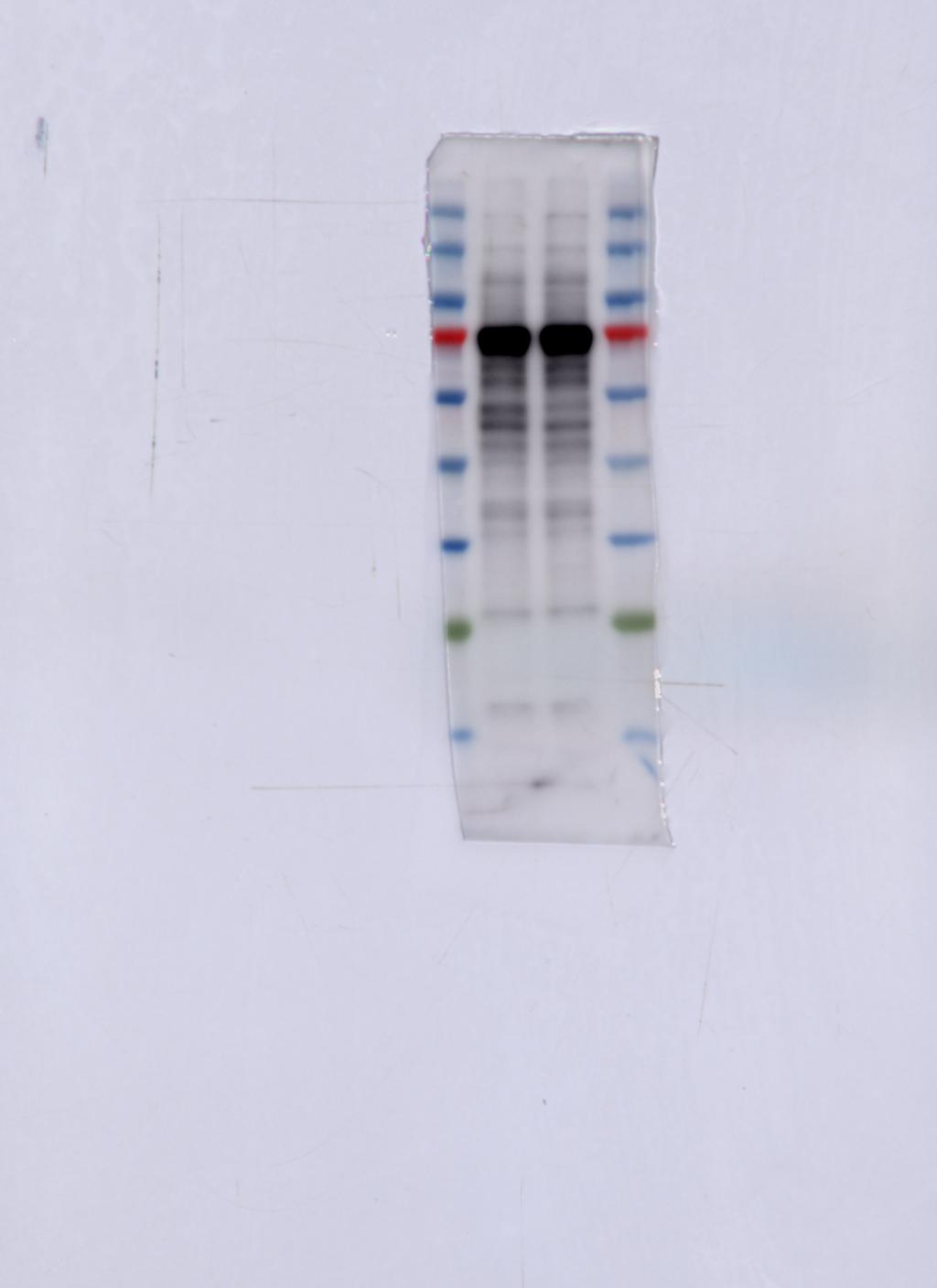

Supplement: Supplementary file 13 — Supplementary Material 13. [file 13046_2025_3438_MOESM13_ESM.zip › full uncropped Gels and Blots image/Fig5B-CHP-1-input-blots.jpg]

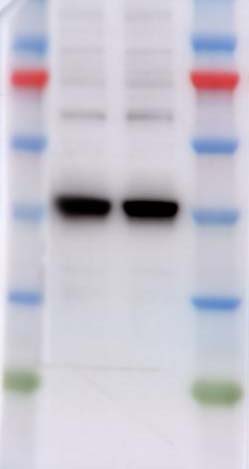

Supplement: Supplementary file 13 — Supplementary Material 13. [file 13046_2025_3438_MOESM13_ESM.zip › full uncropped Gels and Blots image/Fig5B-PD-L1-INPUT-blots.jpg]

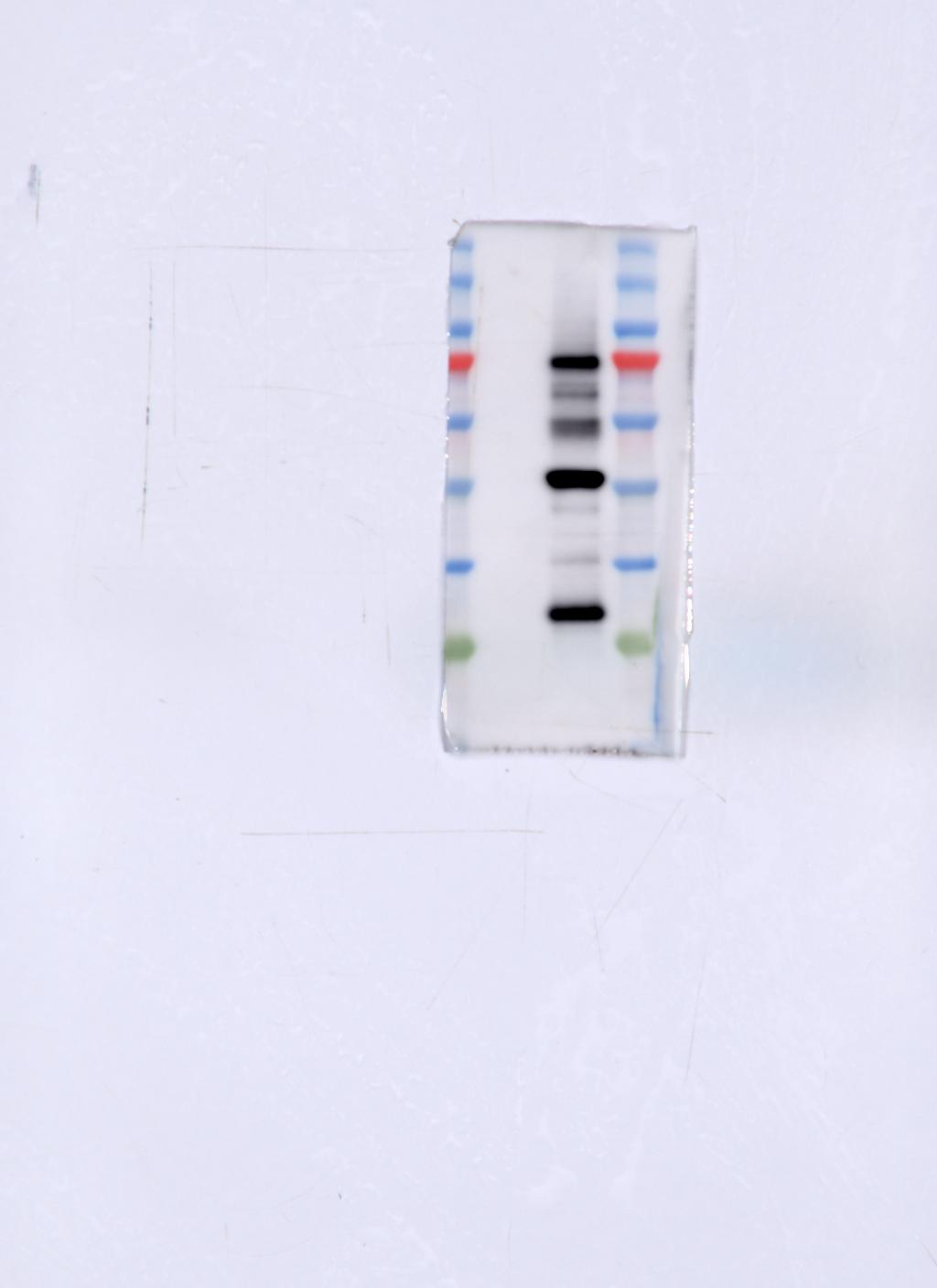

Supplement: Supplementary file 13 — Supplementary Material 13. [file 13046_2025_3438_MOESM13_ESM.zip › full uncropped Gels and Blots image/Fig5B-PD-L1-IP-blots.jpg]

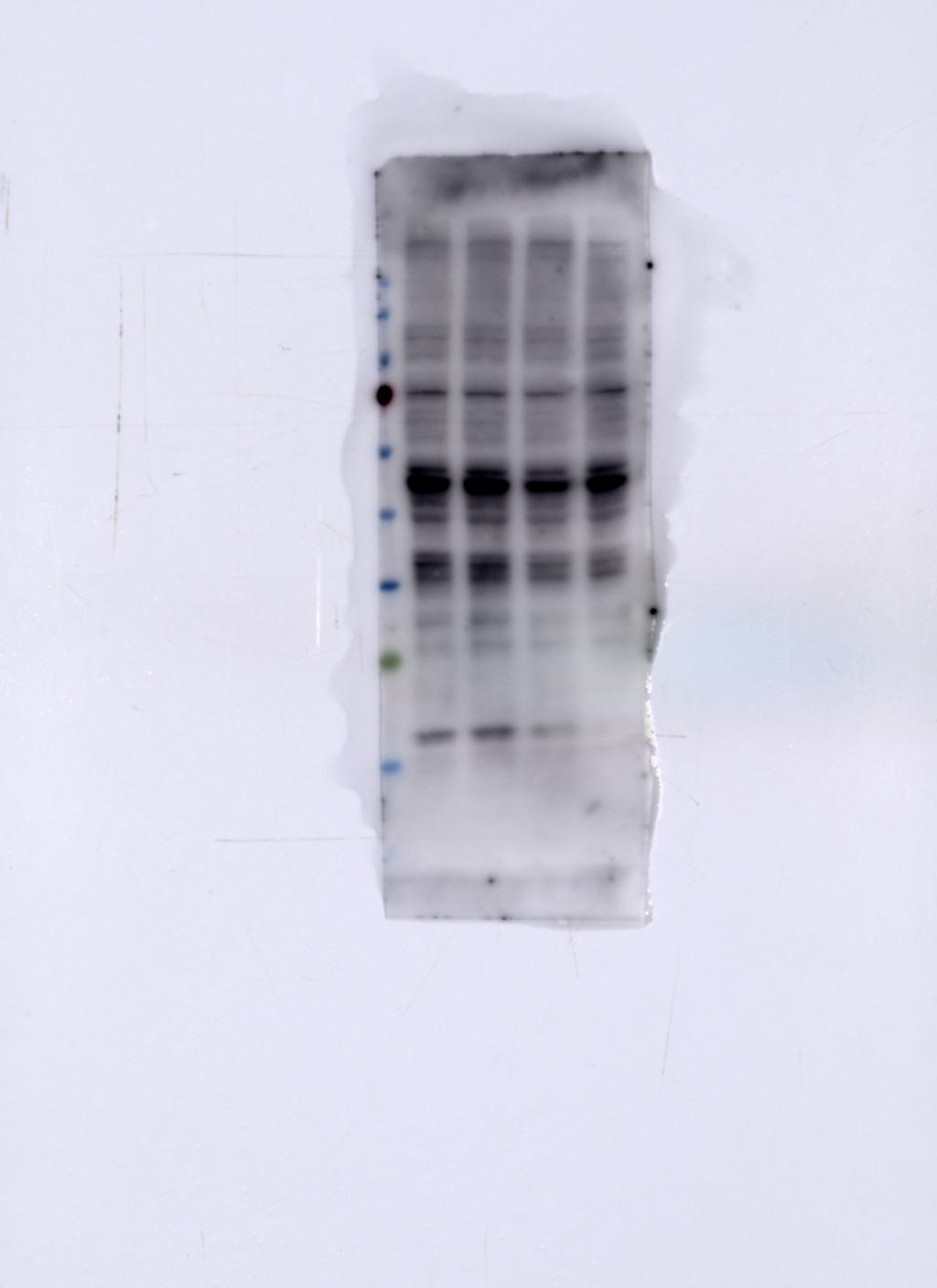

Supplement: Supplementary file 13 — Supplementary Material 13. [file 13046_2025_3438_MOESM13_ESM.zip › full uncropped Gels and Blots image/Fig5C-PD-L1-blots.jpg]

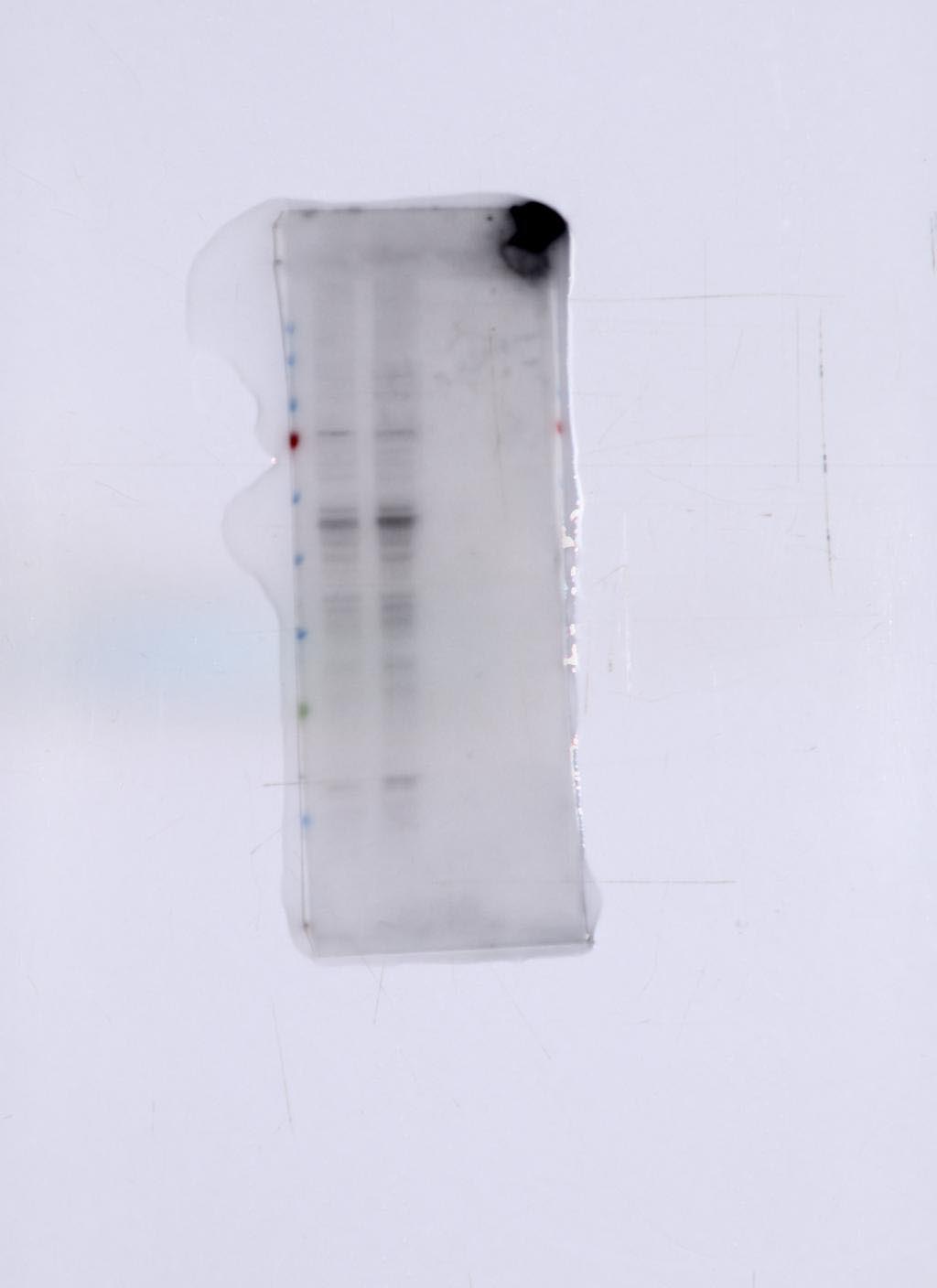

Supplement: Supplementary file 13 — Supplementary Material 13. [file 13046_2025_3438_MOESM13_ESM.zip › full uncropped Gels and Blots image/Fig5C-Ynmyr-PD-L1-blots.jpg]

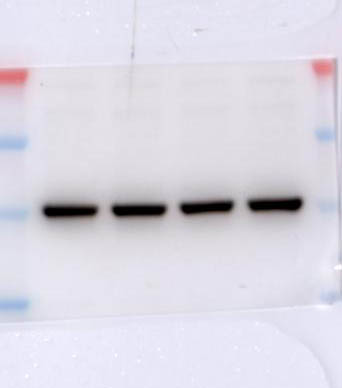

Supplement: Supplementary file 13 — Supplementary Material 13. [file 13046_2025_3438_MOESM13_ESM.zip › full uncropped Gels and Blots image/Fig5E-actin-blots.jpg]

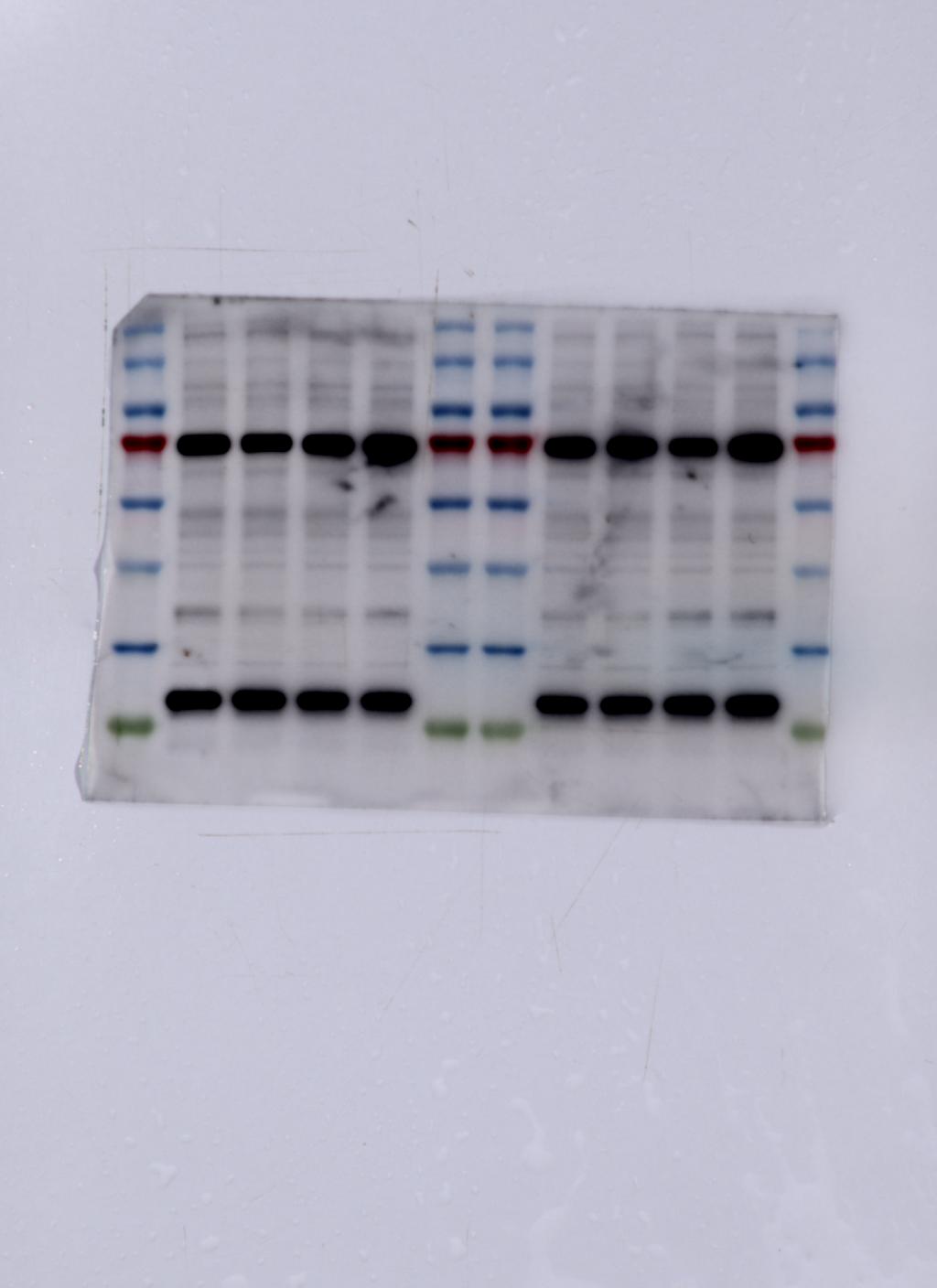

Supplement: Supplementary file 13 — Supplementary Material 13. [file 13046_2025_3438_MOESM13_ESM.zip › full uncropped Gels and Blots image/Fig5E-chp1-input(1-4GEL).jpg]

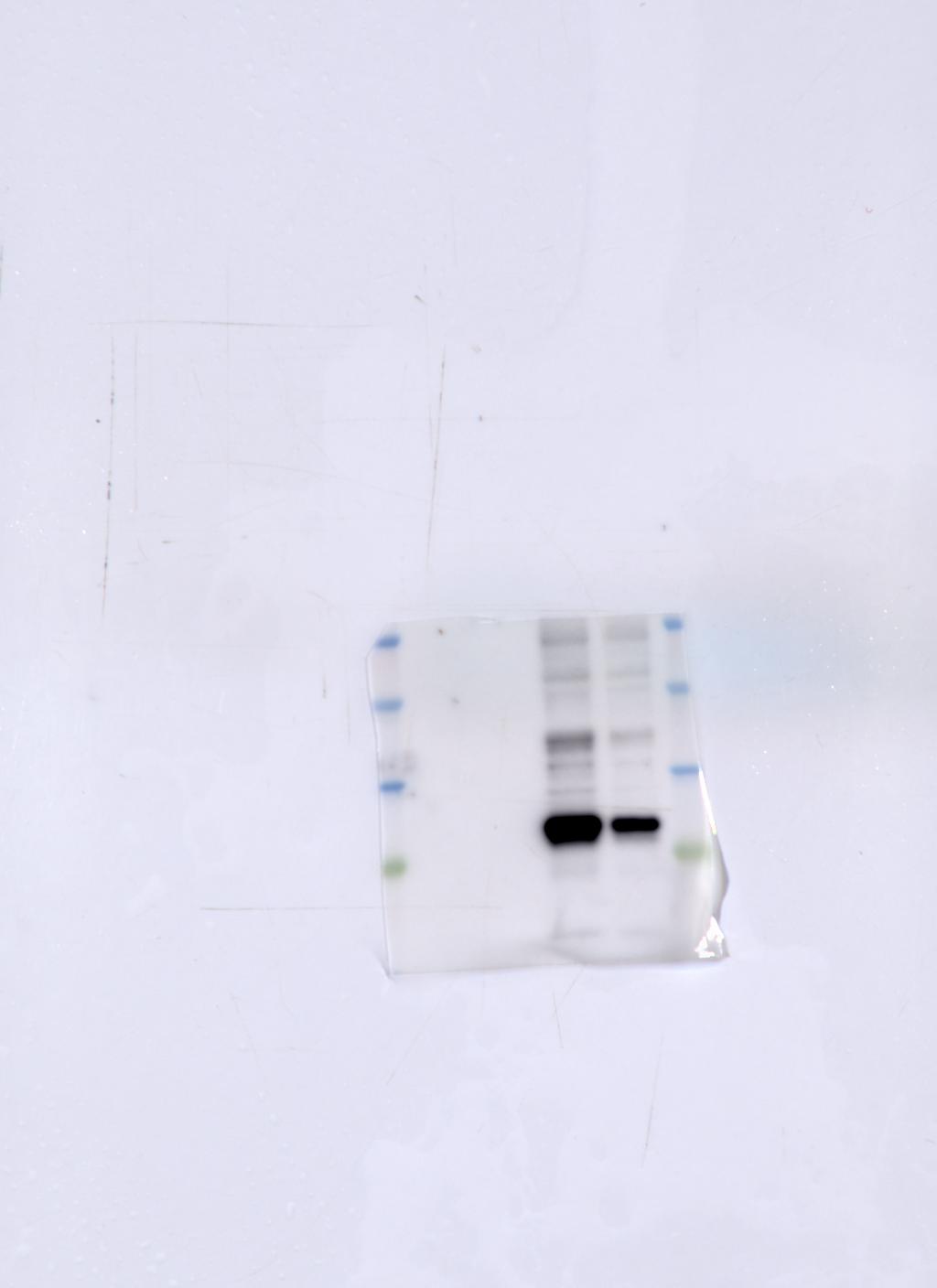

Supplement: Supplementary file 13 — Supplementary Material 13. [file 13046_2025_3438_MOESM13_ESM.zip › full uncropped Gels and Blots image/Fig5E-chp1-ip.jpg]

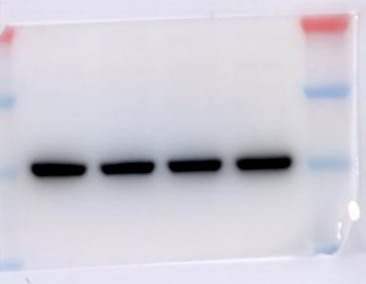

Supplement: Supplementary file 13 — Supplementary Material 13. [file 13046_2025_3438_MOESM13_ESM.zip › full uncropped Gels and Blots image/Fig5G-actin-blots.jpg]

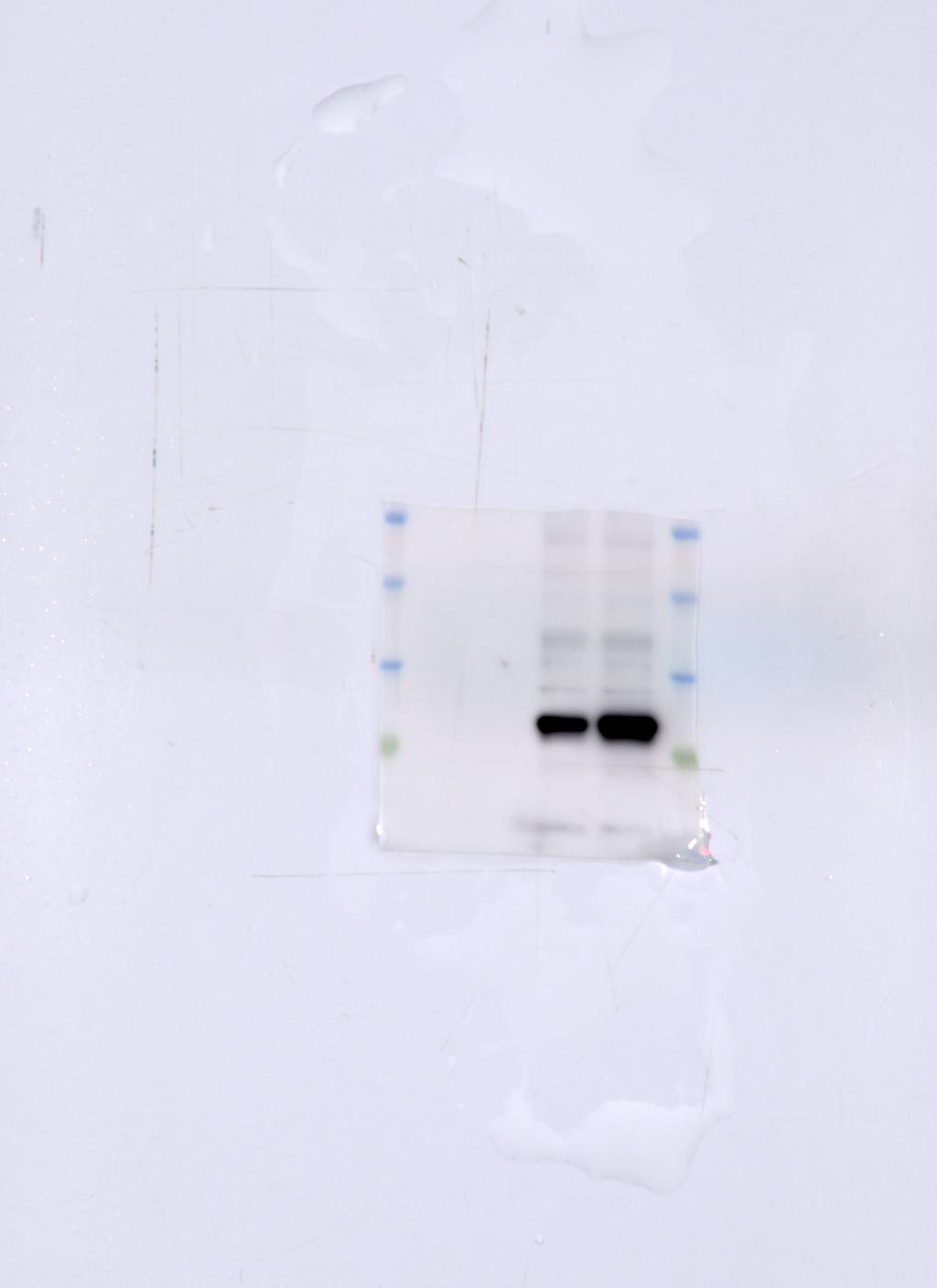

Supplement: Supplementary file 13 — Supplementary Material 13. [file 13046_2025_3438_MOESM13_ESM.zip › full uncropped Gels and Blots image/Fig5G-chp1-ip.jpg]

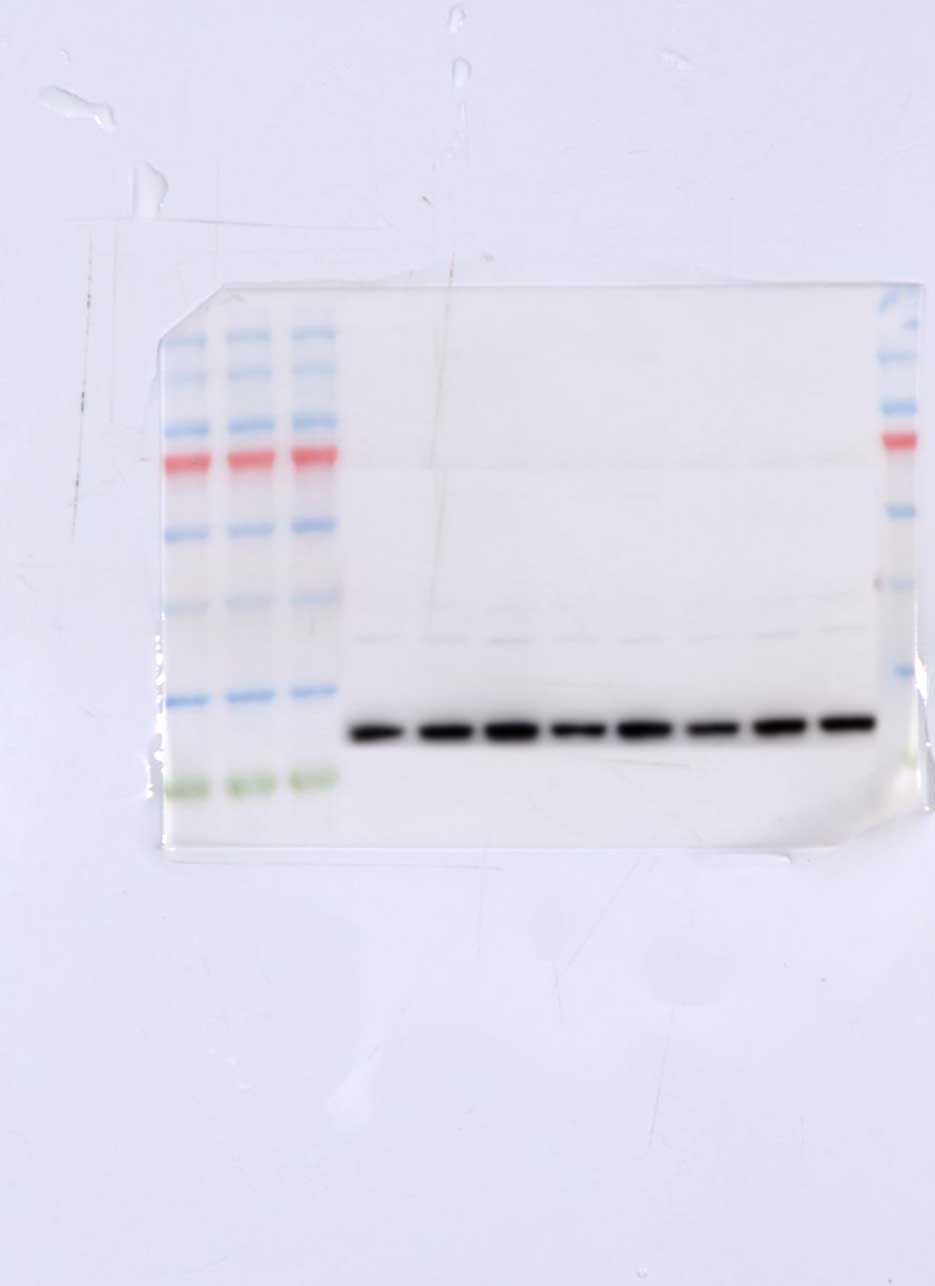

Supplement: Supplementary file 13 — Supplementary Material 13. [file 13046_2025_3438_MOESM13_ESM.zip › full uncropped Gels and Blots image/Fig5J-CHP1-input-blots.jpg]

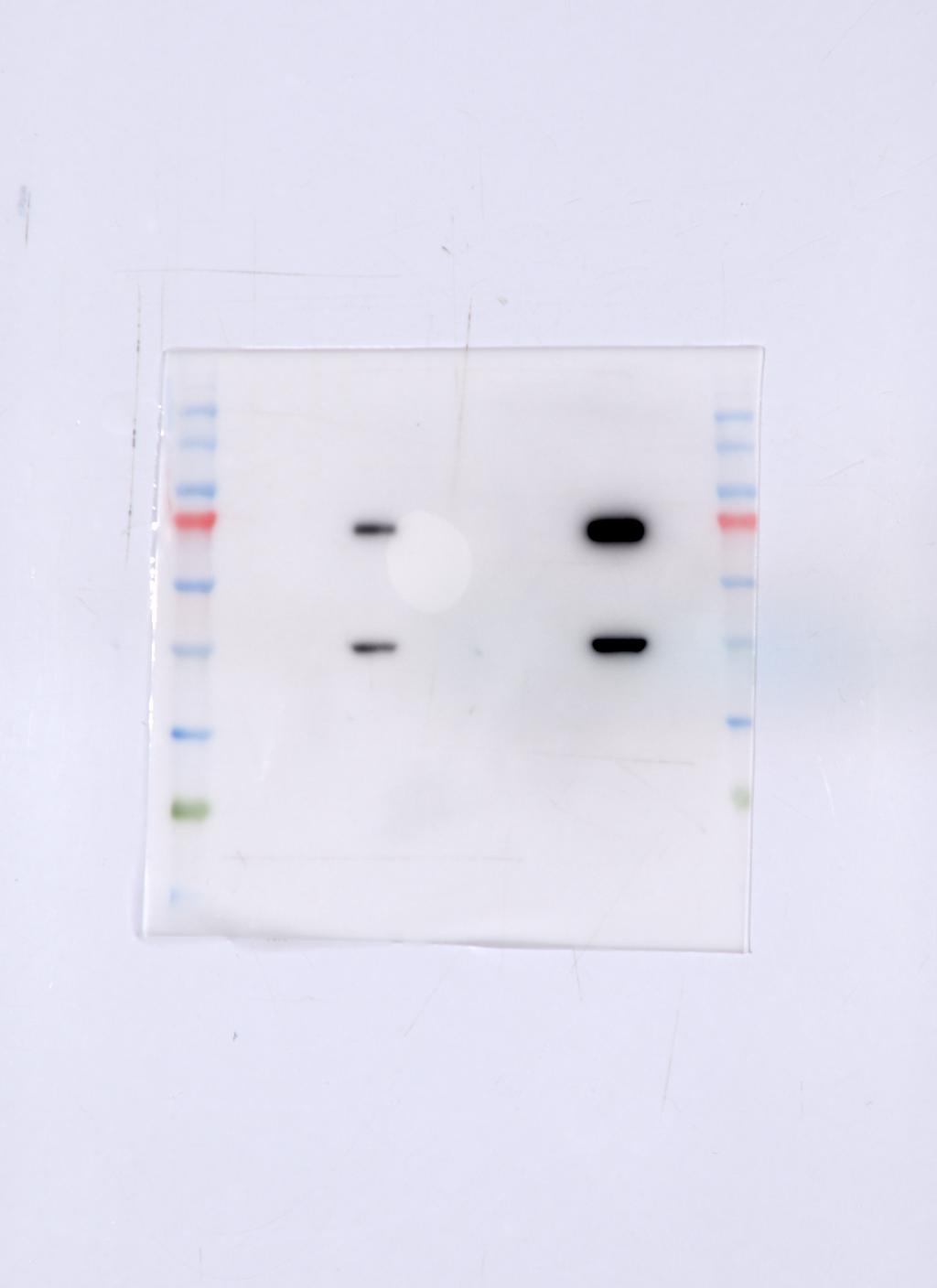

Supplement: Supplementary file 13 — Supplementary Material 13. [file 13046_2025_3438_MOESM13_ESM.zip › full uncropped Gels and Blots image/Fig5J-CHP1-ip-blots..jpg]

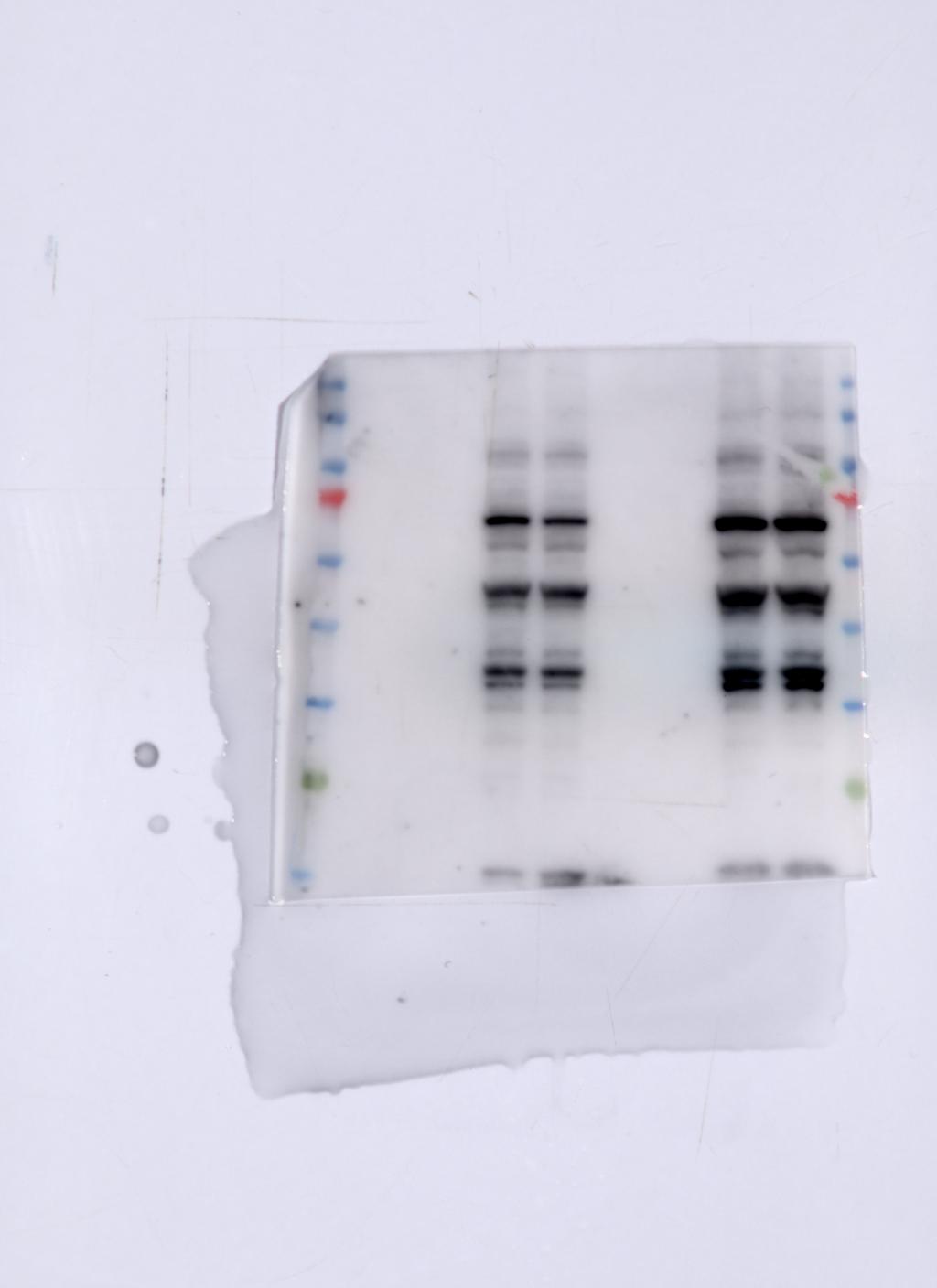

Supplement: Supplementary file 13 — Supplementary Material 13. [file 13046_2025_3438_MOESM13_ESM.zip › full uncropped Gels and Blots image/Fig5J-Pannmyr-blots.jpg]

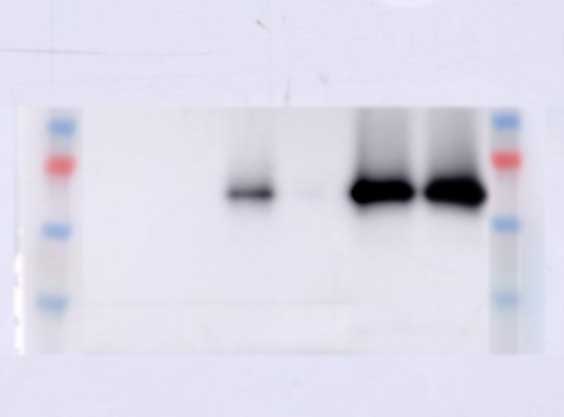

Supplement: Supplementary file 13 — Supplementary Material 13. [file 13046_2025_3438_MOESM13_ESM.zip › full uncropped Gels and Blots image/Fig5L-PD-L1-blots.jpg]

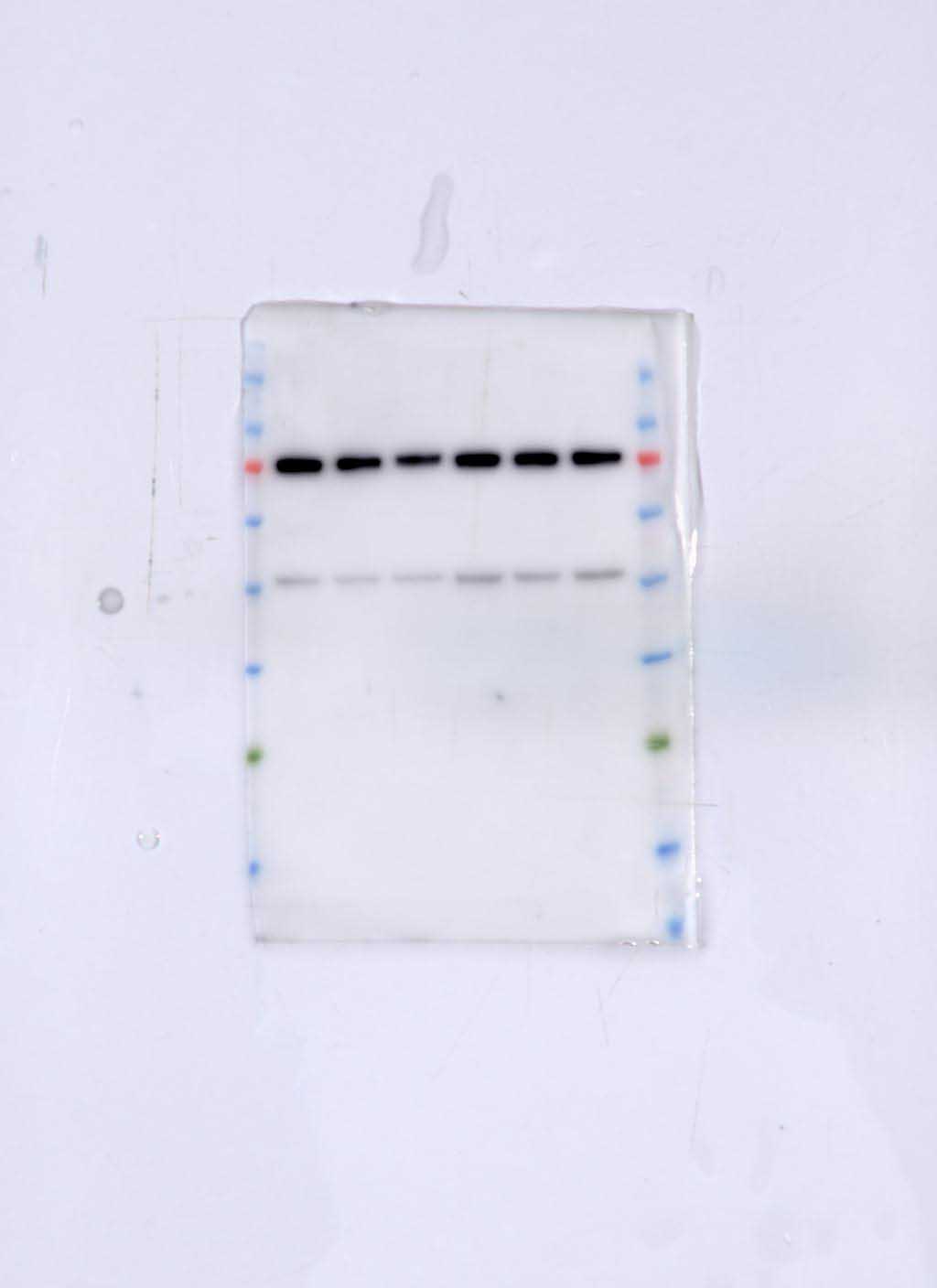

Supplement: Supplementary file 13 — Supplementary Material 13. [file 13046_2025_3438_MOESM13_ESM.zip › full uncropped Gels and Blots image/Fig5L-PD-L1-input-blots.jpg]

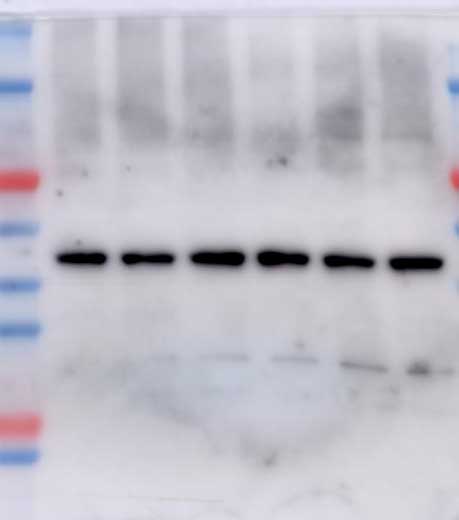

Supplement: Supplementary file 13 — Supplementary Material 13. [file 13046_2025_3438_MOESM13_ESM.zip › full uncropped Gels and Blots image/Fig5L-flag-input-blots.jpg]

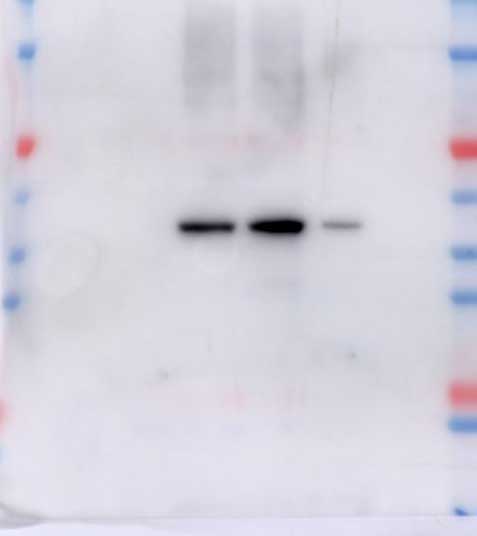

Supplement: Supplementary file 13 — Supplementary Material 13. [file 13046_2025_3438_MOESM13_ESM.zip › full uncropped Gels and Blots image/Fig5L-flag-ip-blots.jpg]

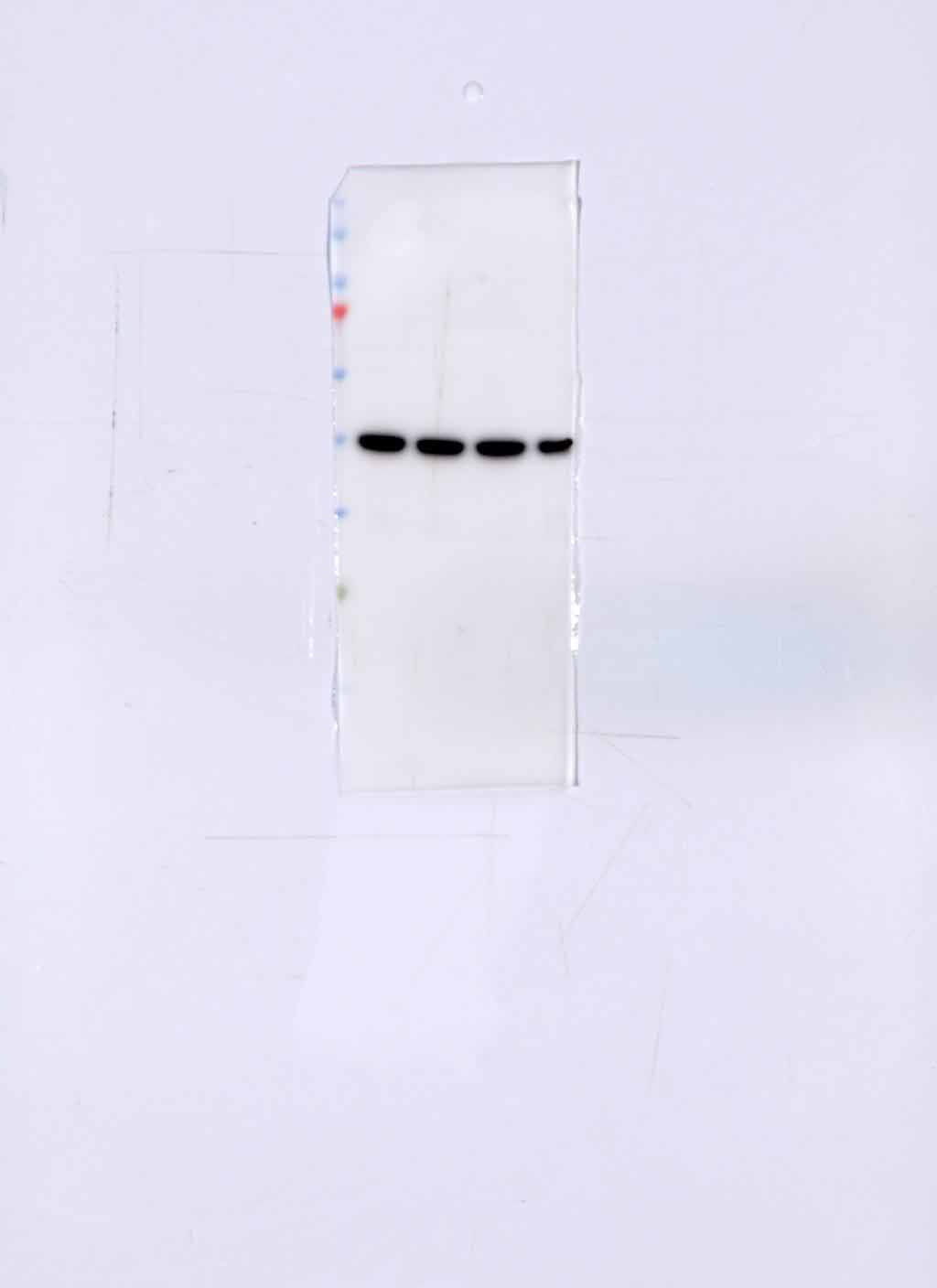

Supplement: Supplementary file 13 — Supplementary Material 13. [file 13046_2025_3438_MOESM13_ESM.zip › full uncropped Gels and Blots image/Fig5c-actin-blots.jpg]

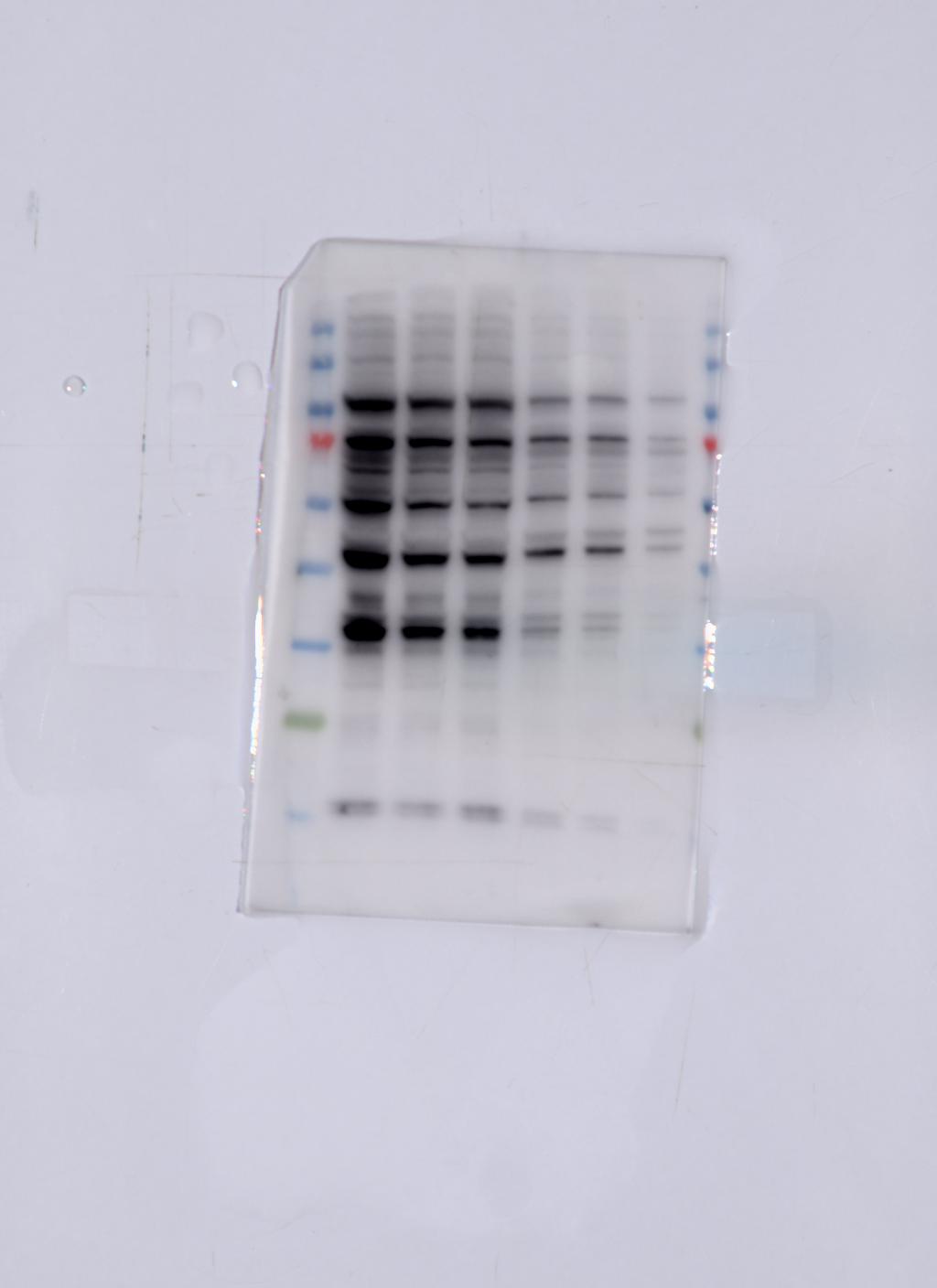

Supplement: Supplementary file 13 — Supplementary Material 13. [file 13046_2025_3438_MOESM13_ESM.zip › full uncropped Gels and Blots image/Fig6A-Pannmyr-blots.jpg]

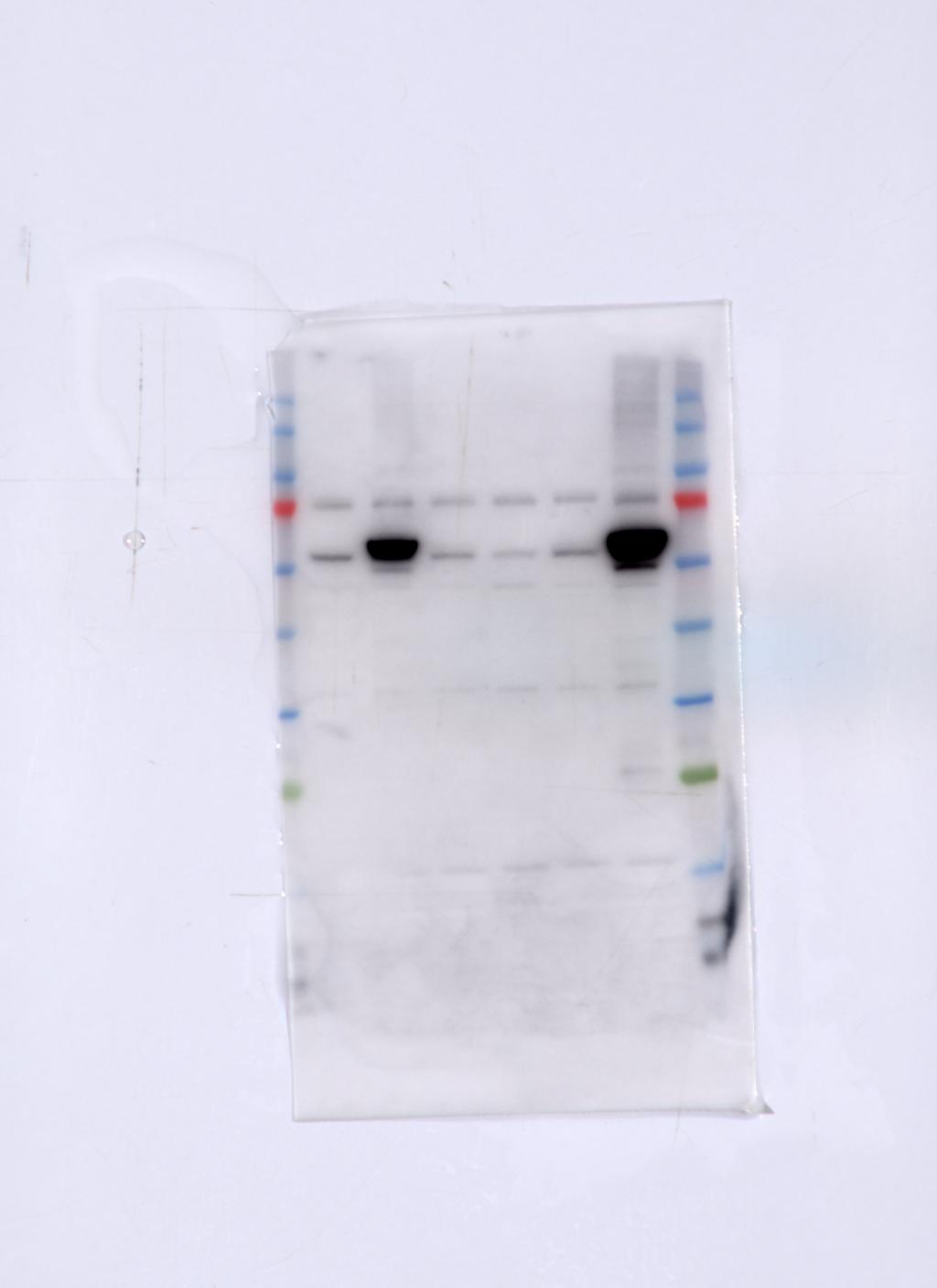

Supplement: Supplementary file 13 — Supplementary Material 13. [file 13046_2025_3438_MOESM13_ESM.zip › full uncropped Gels and Blots image/FigS1-OENMT1-blots(1-2gel).jpg]

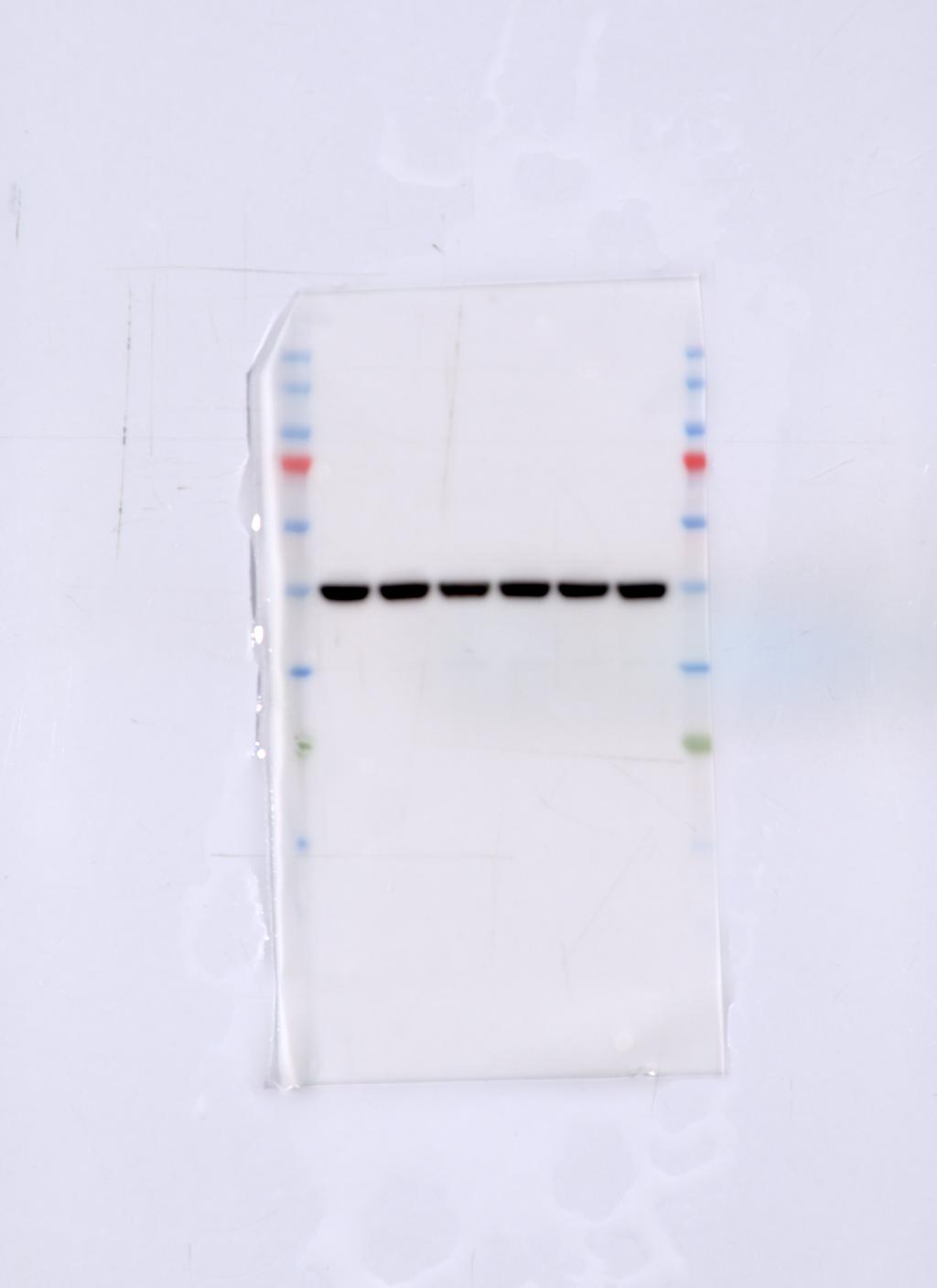

Supplement: Supplementary file 13 — Supplementary Material 13. [file 13046_2025_3438_MOESM13_ESM.zip › full uncropped Gels and Blots image/FigS1-actin(OENMT1)-blots(1-2gel).jpg]

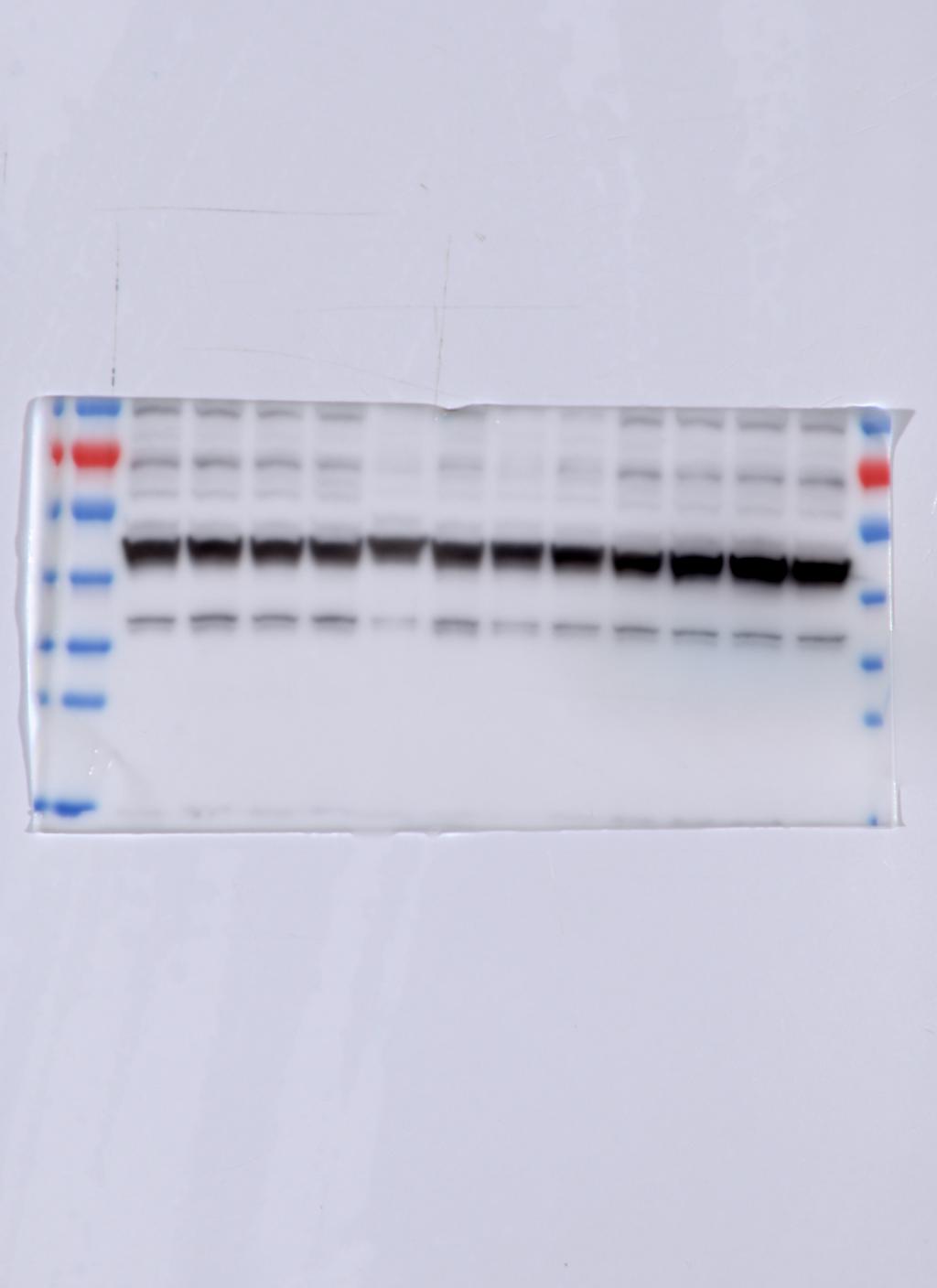

Supplement: Supplementary file 13 — Supplementary Material 13. [file 13046_2025_3438_MOESM13_ESM.zip › full uncropped Gels and Blots image/FigS1B-actin(shNMT1)-blots(1-8gel).jpg]

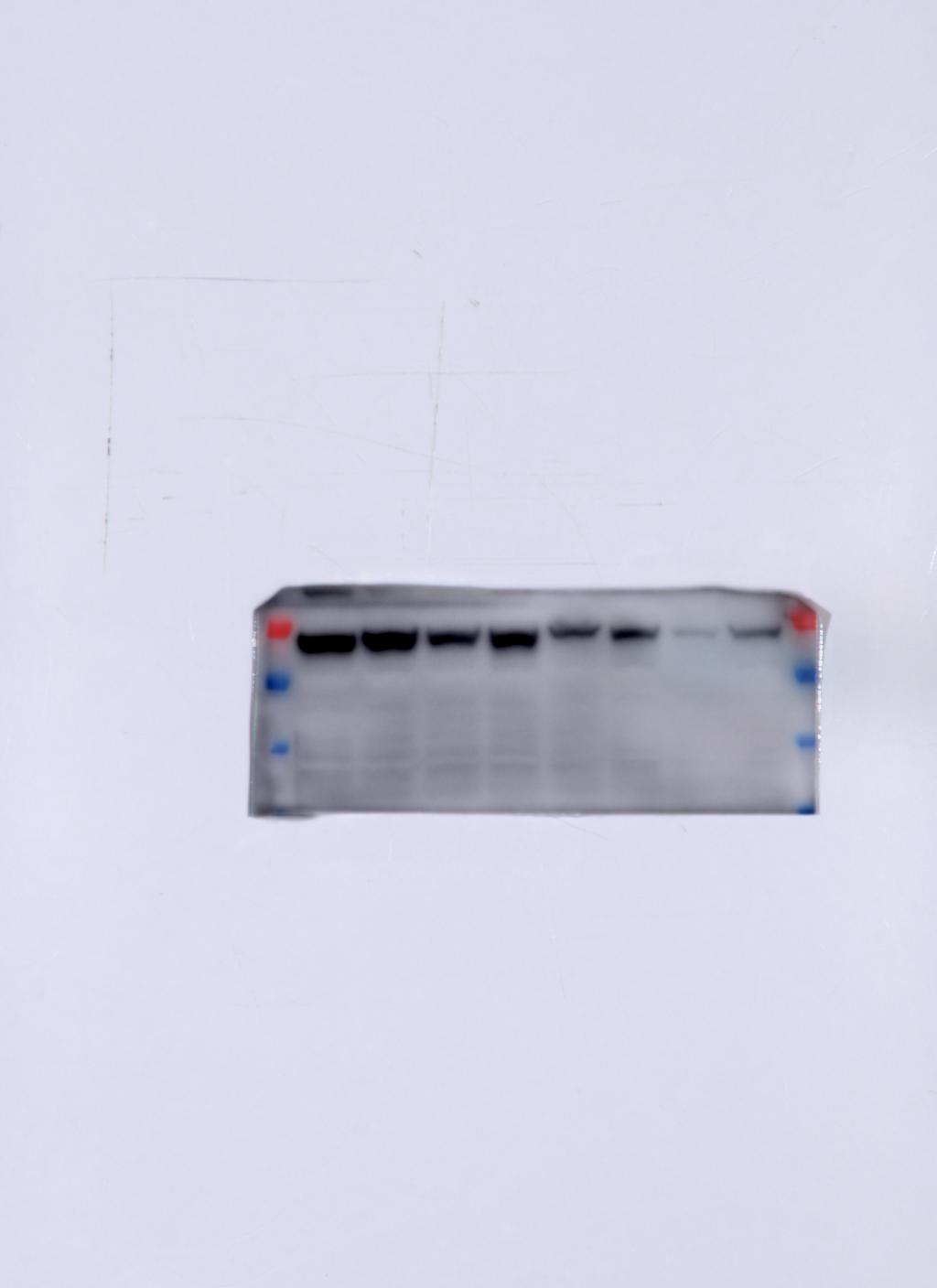

Supplement: Supplementary file 13 — Supplementary Material 13. [file 13046_2025_3438_MOESM13_ESM.zip › full uncropped Gels and Blots image/FigS1B-shNMT1-blots.jpg]

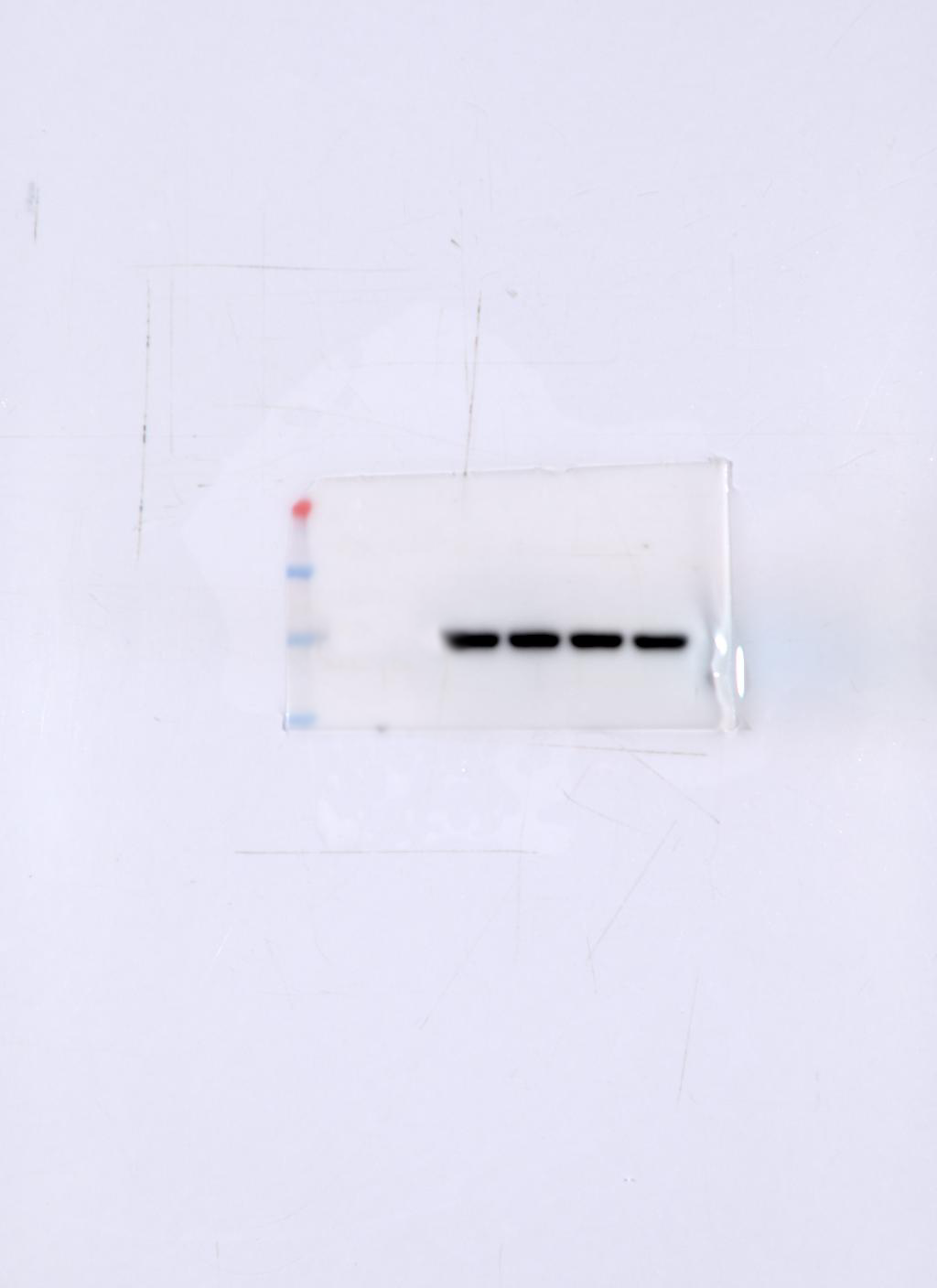

Supplement: Supplementary file 13 — Supplementary Material 13. [file 13046_2025_3438_MOESM13_ESM.zip › full uncropped Gels and Blots image/SupplementaryFig1A-actin.tif]

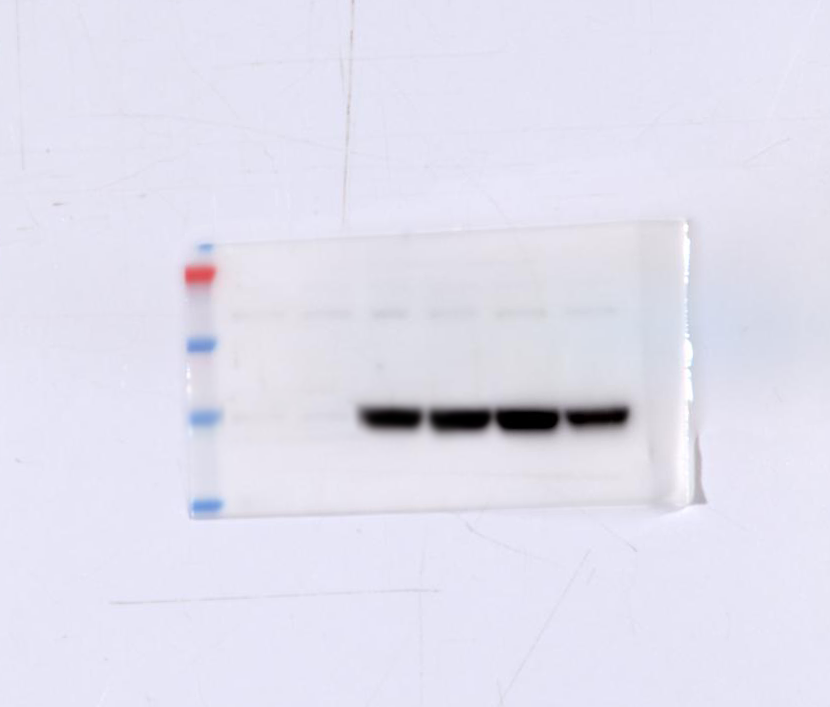

Supplement: Supplementary file 13 — Supplementary Material 13. [file 13046_2025_3438_MOESM13_ESM.zip › full uncropped Gels and Blots image/SupplementaryFig1B-actin.tif]
